# Stable, Highly Conductive, and Strain‐Insensitive Supramolecular Elastomer Composite for Printable Self‐Healing Soft Electronics

**DOI:** 10.1002/advs.202505011

**Published:** 2025-06-25

**Authors:** Ahmed Albeltagi, Tiia Tyystälä, Mikko Nelo, Tuomo Siponkoski, Aldeliane M. da Silva, Mari Rocham, Jari Hannu, Heli Jantunen, Jari Juuti, Jarkko Tolvanen

**Affiliations:** ^1^ Microelectronics Research Unit Faculty of Information Technology and Electrical Engineering University of Oulu P.O. Box 4500 Oulu FIN‐90014 Finland

**Keywords:** conductive inks, liquid metals, soft electronics, stretchable displays, underwater electronics

## Abstract

Stretchable and self‐healing soft conductive materials are essential for soft electronics, robotics, wearables, and bioelectronics. However, achieving a single material that simultaneously offers high and stable conductivity, minimal resistance changes under extreme stretching, high‐resolution universal printability, autonomous self‐healing, and pressure‐sensitive adhesive properties for direct bonding of surface‐mountable components remains challenging. Here, a printable ink composed of liquid metal microparticles and carboxylic acid‐functionalized carbon nanotubes, blended into a bimodal supramolecular elastomer matrix is introduced. After photothermal activation, the material is capable of reorganizing conductive pathways and achieves a high conductivity (> 20000 S·cm^−1^ under strain), exceptional strain insensitivity (*R/R_0_* < 3.95 up to 500%), and an elastic working range >700%. The reversible oxygen‐boron and hydrogen bonding enable both effective autonomous self‐healing and direct assembly of self‐healing hybrid electronic circuits and systems through self‐adhesiveness. To showcase the high performance and functionality, a highly stretchable, self‐healing, and waterproof 3 × 5 pixel display is fabricated.

## Graphical abstract

A printable, self‐healing, stretchable, and highly conductive ink with pressure‐activated adhesive properties is designed by incorporation of liquid metal microparticles and carboxyl‐functionalized carbon nanotubes into a bimodal supramolecular elastomer. After photothermal activation, it achieves conductivity of >20000 S·cm⁻¹, *R/R₀* < 3.95 at < 500% strain, and >700% elastic working range. Demonstrated in a stretchable, self‐healing 3 × 5 pixel display with waterproofing.

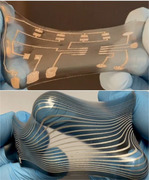

## 1 Introduction

Soft and stretchable electronics materials are becoming a key technological advancement for the next generation of electronics across a diverse range of emerging fields, including soft robotics,^[^ ^1^ ^]^ wearable electronics,^[^ ^2^, ^3^ ^]^ soft electronics,^[^ ^4^, ^5^, ^6^ ^]^ bioelectronics,^[^ ^2^, ^7^ ^]^ healthcare monitoring,^[^ ^8^, ^9^, ^10^ ^]^ and human‐machine interfaces.^[^ ^11^, ^12^, ^13^ ^]^ The demand for high‐performance electrically conductive soft materials has grown exponentially with advancements in materials technologies used for on‐skin and implantable wearable electronics electronic devices. However, the harsher and more dynamic use conditions pose a greater risk for rapid electrical or mechanical failure of the devices. In many cases, repair or replacement of broken, worn, or implantable devices is challenging, or even infeasible. Thus, having a built‐in autonomous material‐level self‐healing functionality is crucial for maintaining the device's performance and functionality for the long term.^[^ ^14^, ^15^, ^16^, ^17^, ^18^, ^19^ ^]^

The ongoing challenge with the design of stretchable conductors not only relates to achieving a stable and high conductivity (> 1000 S·cm^−1^) but simultaneously acquiring a large elastic working range (> 400%) with a minimal change of resistance (*R/R_0_* < 5) despite large stretch.^[^ ^20^, ^21^ ^]^ Despite remarkable advances in soft conductive materials, the morphology of intrinsically conductive polymers is easily disrupted at extreme stretches. Similarly, low‐dimensional nanofillers embedded in elastomers tend to become dislocated at material interfaces, resulting in significant conductivity loss under stretch. For isotropic conductors with constant conductivity (such as liquid metal inks without elastomer matrix) the resistance increases by *(1+ε)^2^* according to Pouillet's law.^[^ ^22^, ^23^ ^]^ To effectively decouple the large geometrical changes and improve conductivity pathways under deformation, dynamically rearranging self‐healing networks could offer potential solutions while allowing complete spontaneous volumetric self‐healing, which is important for addressing the vulnerability of structures in soft functional electronics.^[^ ^24^, ^25^ ^]^

For strain‐insensitive and highly conductive dynamically rearranging self‐healing networks, liquid metal represents one of the most promising electrical fillers as evidenced by the recent advances in stretchable conductors.^[^ ^26^, ^27^, ^28^, ^29^, ^30^ ^]^ However, direct blending of bulk liquid metal with polymer phases results in poor interfacial compatibility, inhomogeneous particle distribution, and limited controllability in printed electronics manufacturing. To address this, liquid metal microparticles (LMMPs) with thin, uniform, and electrically insulative oxide skin are used to achieve good dispersion within soft matrices and enable processability.^[^ ^26^, ^27^, ^28^ ^]^ To render materials highly conductive, mechanical, photothermal, or chemical activation is needed to disrupt, or completely remove, the oxide layer following the material deposition.^[^ ^29^, ^30^, ^31^, ^32^ ^]^ However, mechanical activation is difficult to apply to autonomous self‐healing polymer matrices due to their limited elasticity and resilience under large strain.^[^ ^18^ ^]^ Typically, compressive stresses of > 1 MPa or tensile strain > 100% are needed to rupture the oxide skin^[^ ^27^, ^28^, ^33^ ^]^ which can induce irreversible deformation or viscous flow in the self‐healing polymer networks. Similarly, chemical or photothermal activation can deteriorate the self‐healing matrix by interfering with reversible bonding or plasticizing the networks, even when thermal decomposition is avoided. Beyond electrical and electro‐mechanical performance, incorporating self‐healing functionality and built‐in adhesive properties for bonding surface‐mountable components directly into conductors remains a largely underexplored yet critical technical challenge for enabling printable self‐healing soft electronics (Table S1, Supporting Information).^[^ ^14^, ^34^, ^35^ ^]^ Achieving robust mechanical electrical bonding to both rigid components and soft polymer phases would require strong interfacial adhesion by the liquid metal. This may necessitate the formation of a porous oxide or solid particle‐rich interfacial network that enables mechanical interlocking and wetting toward rigid surfaces, while additional surface modification strategies–such as functionalized nanomaterials–may be required to improve the compatibility and adhesion with the surrounding self‐healing polymer matrix. However, viable material design strategies for achieving an all‐in‐one, multifunctional, autonomously self‐healing conductor with stable, high conductivity and excellent electro‐mechanical performance have yet to be realized.

Herein, to fully address all the above‐mentioned challenges, we present a novel design strategy by blending liquid metal microparticles (LMMPs) dispersion with carboxylic acid‐functionalized multiwalled carbon nanotubes (MWCNT‐COOH) into self‐healing supramolecular elastomer. This forms a printable electrically conductive liquid metal elastomer (ECLME). The printing resolution of ECLME ink was less than 50 µm with blade coating using polymeric shadow masks. To achieve a highly stable conductive network with ECLME, we use photothermal activation on the printed, low‐temperature curable (≤ 70 °C) ECLME ink. This results in the formation of heterogeneous LMMP‐rich regions within the 3D supramolecular elastomer network originating from the homogenously distributed and isolated LMMPs at the initial stage. The photothermally activated ECLMEs achieved a maximum zero strain conductivity up to > 9600 S·cm^−1^, approaching the bulk conductivity of gallium‐based liquid metals (≈35000 S·cm^−1^). Under stretching, dynamic reorganization of the conductive pathways resulted in minimal resistance changes (*R/R_0_* < 3.95 at *ε* ≤ 500%). Furthermore, we showed the compatibility of ECLME ink with different soft substrate materials by using a self‐healing intermediate layer (made of the same supramolecular elastomer). The use of a self‐healing intermediate layer with ECLME ink resulted in significant performance improvement on non‐self‐healing substrates. We further demonstrated that the ECLME can spontaneously self‐heal and recover its original electrical properties and functionality without any need for external intervention or energy‐input triggers. The built‐in self‐healing functionality enables also direct pressure‐activated self‐bonding of rigid and semi‐rigid components to the surface of ECLME without any need for adhesives. ECLME was highly stable across a wide range of temperatures and relative humidity, under static and dynamic loading conditions, with negligible change of resistance. We highlight the exceptional properties and functionality of the printable self‐healing soft electronics by fabricating a highly stretchable and self‐healing display with waterproof functionality. The ECLME‐based 3 × 5 pixel display was stretchable up to > 200% and maintained the function even when immersed underwater for > 2 months.

## 2 Results and Discussion

### 2.1 ECLME Design Concept

**Figure 1:**
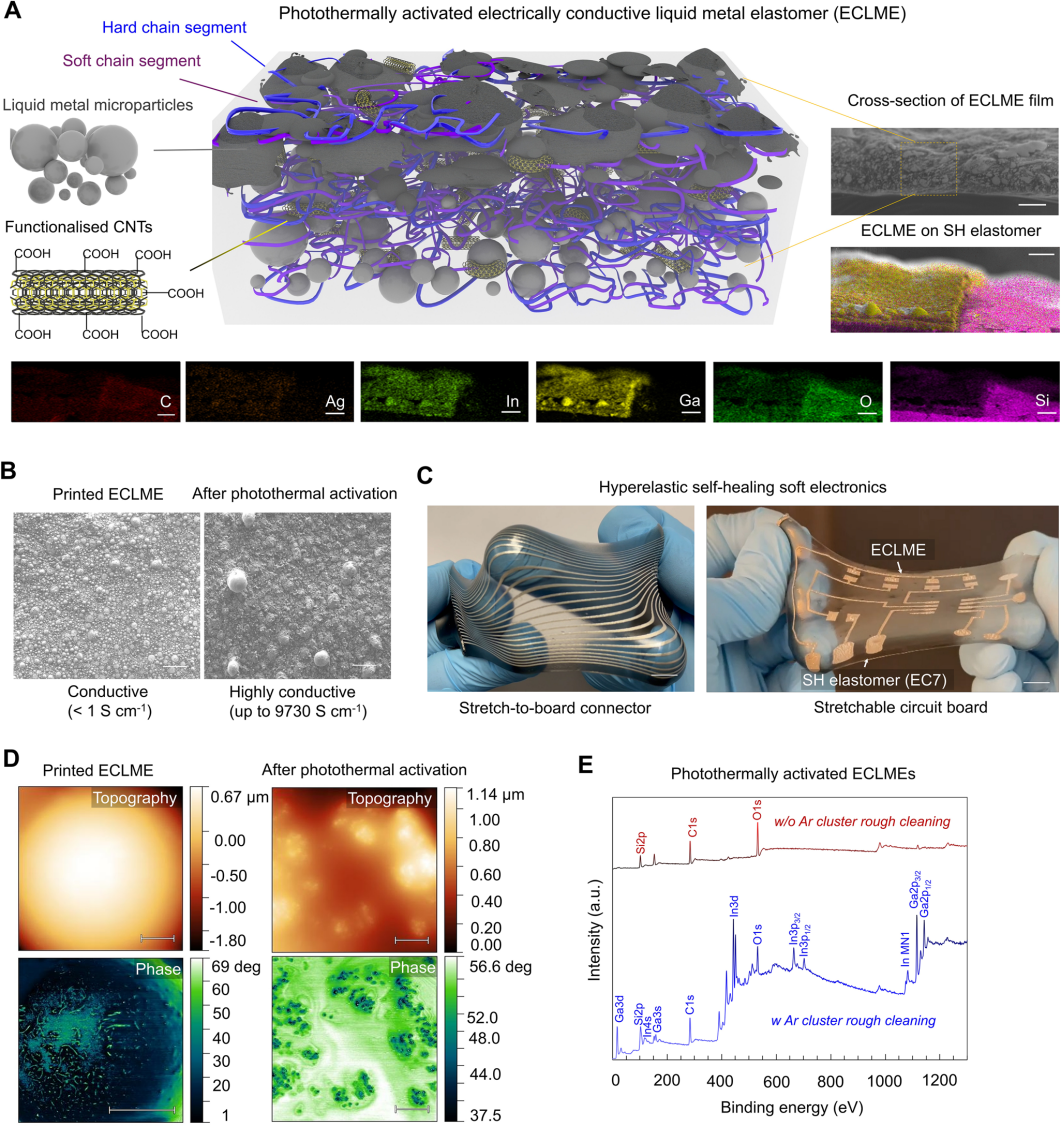
Electrically conductive liquid metal elastomer. a) Schematic illustration of the ECLME, cross‐sectional FESEM, and EDS of the ECLME film. Scale bars 50 and 100 µm, respectively. b) FESEM images of printed films before and after photothermal activation. Scale bars 100 µm. c) Printed ECLME stretch‐to‐board connector and circuit board on self‐healing elastomer. Scale bars 10 mm. d) Topography and phase AFM images of as printed and photothermally activated ECLME films. Scale bars 1 um. e) XPS analysis of photothermally activated ECLMEs with (w) and without (w/o) Ar cluster rough cleaning.

The schematic illustration of the hyperelastic, self‐healing, and electrically conductive liquid metal elastomer (ECLME) is illustrated in **Figure** **1**a. The proposed material design concept enables an extraordinary combination of properties, including stable and high conductivity (> 9600 S·cm^−1^; further increasing with strain), strain‐insensitivity (*R/R_0_* < 3.95 at *ε* ≤ 500%), autonomous self‐healing in ambient conditions, and pressure‐activated adhesiveness allowing direct bonding of both rigid and semi‐rigid surface‐mountable components to the conductor's surface. This combination of properties has been difficult to achieve simultaneously. Liquid metal‐based stretchable conductors typically lack complete autonomous self‐healing functionality—particularly in terms of electro‐mechanical properties—and/or built‐in adhesive properties for the direct bonding of components. Conversely, autonomous self‐healing conductors have so far not successfully integrated LMMPs in a way that achieves high conductivity, strain insensitivity (Tables S1 and S2, Supporting Information), and efficient electrical and electro‐mechanical self‐healing functionality.

To achieve a highly stable, dynamically reconstructing, and reversible conductive network with both strong interfacial bonding and adhesion, we used our previously reported self‐healing supramolecular elastomer^[^ ^18^ ^]^ having a unique combination of weak hydrogen bonding and strong dynamic covalent bonds. Our previously reported supramolecular elastomer consists of a bimodal interpenetrated network made by cross‐linked poly(borosiloxane)‐dimethylvinyl‐terminated dimethylsiloxane‐based elastomer. In this work, we used specific elastomer composition with the optimized tensile and self‐healing properties (denoted as EC7; which refers to the elastomer composition 7 developed in our previous work).^[^ ^18^ ^]^

To enhance the tensile properties, microstructural stability, and creep resistance without compromising the inclusion of liquid metal microparticles (LMMPs), we incorporated carboxylic acid‐functionalized multiwalled carbon nanotubes (MWCNT‐COOH) into the elastomer. The high aspect ratio MWCNT‐COOH facilitates the formation of a strong, tough, and highly elastic network with improved interfacial bonding between the CNTs and the elastomer. The carboxylic groups (─COOH) enable strong interactions with the bimodal polymer chains potentially through hydrogen bonding and van der Waals‐forces which improves the dispersion of CNTs, and load transfer at the material interfaces. These interactions also promote the formation of a highly entangled and mechanically interlocked network, efficiently stabilizing the cross‐links, and thereby reducing the macromolecular interdiffusion in a controllable manner. The complex entangled 3D elastomer network formed with CNTs also creates torturous paths for the diffusion of moisture and oxygen, mitigating the impact of environmental conditions. Consequently, slowing down the reversible condensation/hydrolysis of Si:O‐B bonds significantly enhances the microstructural stability of the elastomer network.^[^ ^18^ ^]^

To achieve a stable and highly conductive network using EC7 matrix with MWCNT‐COOH, we first prepared dispersion of liquid metal microparticles (LMMPs) through a short probe ultrasonication process (5–10 min) in a water bath without active cooling (Figures S1 and S2, Supporting Information). Then, the LMMP dispersion could be physically blended with the supramolecular elastomer components to form stretchable, self‐healing, and highly conductive ECLME ink. Instead of adding MWCNT‐COOH directly to the base elastomer, we formed the LMMP dispersion with CNTs by probe ultrasonicating eutectic gallium‐indium (eGa_75.5_In_24.5_), MWCNT‐COOH, and isopropanol (Figure S2, Supporting Information). The addition of MWCNT‐COOH both stabilized the dispersion and, at the later stages, enabled the homogenous dispersion of LMMPs to the 3D elastomer network (Figure S3, Supporting Information).^[^ ^36^, ^37^ ^]^

In ECLME, a large number of hydrogen bonds (with dissociation energy of ≈10–50 kJ mol^−1^) are formed due to hydrogen bonding between the possible formed surface oxide hydroxyl groups, oxygen‐boron (O─B) and oxygen‐hydrogen bonds (O─H).^[^ ^38^, ^39^, ^40^, ^41^, ^42^, ^43^ ^]^ More importantly, the electron deficiency of O─B linkages favors the formation of more stable coordination bonding (≈100–300 kJ mol^−1^)^[^ ^38^, ^39^, ^40^, ^41^, ^42^, ^43^ ^]^ with the electron‐rich oxygen sites on the metal oxides (e.g., Ga_2_O_3_, In_2_O_3_). This can strengthen the interaction at the polymer‐oxide interface. The presence of O─B coordination bonding and hydrogen bonding significantly enhances the adhesion and cohesion of the network, while the latter enables the dynamic rearrangement of the conductive pathways. The reversible O─B bonds (≈ 806 kJ mol^−1^) and B‐O─C linkages (≈200–300 kJ mol^−1^),^[^ ^38^, ^39^, ^40^, ^41^, ^42^, ^43^ ^]^ formed with the oxygen rich siloxane‐network, provide structural integrity for the elastomer network, while also allowing efficient self‐healing to take place. The carboxyl group (─COOH) in functionalized MWCNT may form a coordination bond with surface oxides, for example, when the electron‐rich oxygen atoms in carboxylate (─COO─) group donate electron density to Ga^2+^ and In^2+^ metal centers.^[^ ^36^ ^]^ The coordination bonding could enable strong anchoring to the surface oxides and dispersion of CNTs within the polymer. Considering that the CNTs also form a highly entangled and mechanically interlocked network with the supramolecular elastomer, they contribute to both adhesion and cohesion within the material. These interactions and bonding prevent the cracking of oxide layers in the heterogeneous LMMP regions, even under large stretches. Thus, achieving highly stable conductive pathways and minimal resistance change at extreme deformations (*ε* > 500%) was possible due to the efficient conduction across the material interfaces without blocking the electron transport.

After the blade coating of ECLME ink to the substrate and low temperature curing (at 50–70 °C for >30 min), LMMPs were homogenously distributed throughout the supramolecular elastomer network (Figures S3–S8, Supporting Information). Even at high volume fractions of liquid metal (*φ*_f‐LM_ > 55%), the LMMPs remained electrically isolated from each other due to surface oxide layers and the thin polymer coating surrounding them. To render the printed ECLME layer highly electrically conductivity (> 1000 S·cm^−1^), we used a photothermal activation with a laser processing system operating at a wavelength of 355 nm. During photothermal activation, the localized heating causes the coalescence of isolated LMMPs. This results in the formation of heterogeneous LMMP‐rich regions within the ECLME (Figure 1b; Figure S5, Supporting Information), along with a drastic increase of electrical conductivity (by a factor up to ≈10^8^) as continuous conductive pathways form with the LMMP aggregation. The efficient photothermal activation of ECLME was enabled by excellent thermal stability and dynamic chemical interaction within the supramolecular elastomer. During photothermal activation, the boranol groups (─B(OH)_2_) at the polymer chain ends undergo a reversible condensation reaction, leading to the formation of boraxane linkages (─B‐O─B‐). This transition results in a temporary, and thermally stable rubbery‐like boroxane soft phase which reduces the macromolecular interdiffusion; crucial for ensuring the permanent photothermal activation of ECLME.

The ECLME ink is directly compatible with a wide range of scalable printed electronics manufacturing methods, including but not limited to screen printing, blade, and bar coating. In this work, we particularly focused on the blade coating by hand with the ECLME ink. As shown (Figure S4, Supporting Information), the formation of tens‐of‐micrometer‐thin printed ECLME layers was possible after photothermal activation, achieved at high resolution without the need to further optimize the rheological properties. The printing of ECLME was possible even by hand to any soft substrate at high resolution (minimum line widths up to ≈39.83 ± 4.75 µm) using high‐quality laser‐cut polymeric shadow masks (Figures S5–S6 and S9–S11, Supporting Information). To improve the compatibility of ECLME ink with otherwise incompatible soft substrates, we also demonstrated the use of an intermediate layer made of the same supramolecular elastomer (used as the base material for ECLMEs). The excellent printability of ECLME allowed the fabrication of self‐healing circuit‐board interconnections and electronic circuits for soft electronics devices of various sizes and at high resolution (Figure 1c; Figure S12, Supporting Information), using effortless hand‐performed blade coating with polymeric shadow masks. The functional structures were capable of withstanding reversible extreme uni‐ and multiaxial deformations even exceeding 2200% strain (Figure S12, Supporting Information).

### 2.2 Material Characterization

Dynamic light scattering with Zetasizer Nano ZS reveals particle size distributions showing three distinct peaks (Figures S1 and S3, Supporting Information). The sizes of LMMPs ranged from ≈58 to 5560 nm for the diluted LMMP dispersions. A median particle (*d_50_*) of 178.6 ± 37.7 nm was achieved only after ≈5–10 min of probe ultrasonication (at 100 W, 30 kHz, 0.5 cycles, 100% amplitude) in a water bath without the need for active cooling (further verified by multiple samples and consecutive measurements). Considering that the blade‐coated ECLME layers were thin (≈25–75 µm, depending on the composition; Figures S3–S8, Supporting Information), the size of LMMPs provides a good balance between the required filler volume fraction, dispersibility in the elastomer, printability, and the electrical, mechanical, and self‐healing properties of the ECLMEs. If the size of the LMMPs were further decreased, achieving good dispersion and formation continuous of the LMMP regions after photothermal activation of the ECLME ink would become difficult.

Due to the addition of carboxylic acid‐functionalized multiwalled carbon nanotubes (MWCNT‐COOH), the LMMP‐dispersion required longer probe ultrasonication time (up to 10 min in a water bath (at 100 W, 30 kHz, 0.5 cycle, 100% amplitude)) to efficiently break up the agglomerates and achieve desirable *d_50_* for LMMPs (Figure S3, Supporting Information). Due to the high aspect ratio, CNTs are prone to entangling and formation of bundles. In this case, the presence of COOH‐functional groups likely increases the inter‐tube attraction (via hydrogen bonding) which reduces dispersion. The presence of ‐COOH results in steric hindrance and forms spatial barriers, while the development of surface charge in isopropanol promotes electrostatic attraction and repulsion. These effects may further increase the van der Waals forces, leading to enhanced entanglement and aggregation, which require longer acoustic cavitation.

The field electron scanning electron microscopy (FESEM) images reveal the presence of MWCNT‐COOH in the LMMP dispersion (Figure S3, Supporting Information), and their polar interactions with the liquid metal. Both of these have an important role in achieving a stable dispersion (Figure S2, Supporting Information),^[^ ^37^, ^44^, ^45^, ^46^ ^]^ and by promoting the homogenous distribution of individual LMMPs throughout the surface and volume of the supramolecular elastomer network (Figures 1a,b; Figures S3, S4, and S7, S8, Supporting Information). We hypothesize that the increased stability of dispersion was related to the formation of metal‐carboxylate bonds involving both ligand interaction and coordination bonding. For example, carboxylate ions (RCOO─) likely act as the ligand, thus negatively charged oxygen atom of RCOO‐donates electron pair(s) to the metal ion(s) forming a coordination complex. ‐COOH group can also dissociate when dissolved in polar solvents, and form carboxylate ions (─COO─) in coordination with the metal atoms positioned on the oxygen‐containing functional groups on the surface oxide of LMMPs (i.e., forming the metal‐carboxylate coordination bonds).^[^ ^37^, ^44^, ^45^, ^46^ ^]^

Without MWCNT‐COOH, the formation of well‐percolated LMMPs and a highly conductive network was not possible (Figure S3, Supporting Information) due to shear force‐induced preferential alignment and direction‐dependent heterogeneous distribution of LMMPs during blade coating. The addition of MWCNT‐COOH serves another functionality in the ECLME by improving the volumetric connectivity of the LMMP network after photothermal activation, as the CNT volume loading (*φ*_f‐CNT_) increases from 0.25 to 0.75% (Figure S4). We found that the composition of the self‐healing substrate does not affect the microstructure of the blade‐coated ECLME layer—for example, when prepared with or without surfactant (Triton X‐100) (Figure S8, Supporting Information).

The as‐printed ECLMEs were poorly electrically conductive (< 1 S·cm^−1^) without the photothermal activation due to the formation of homogenously distributed and isolated LMMPs with uniformly formed surface oxide layers within the 3D elastomer network. Photothermal activation results in localized heating that breaks and reforms the oxide after the coalescence of the individual particles (Figure S13, Supporting Information). More specifically, upon laser irradiation, the absorbed energy is rapidly converted into heat as the laser's excitation rate is insufficient to trigger photochemical reactions. This localized heating causes rapid thermal expansion of the liquid metal core within the core–shell structure of LMMPs (Figure S13, Supporting Information). Due to mismatches in thermal expansion coefficients and thermal conductivities between the liquid metal core and its surrounding oxide shell, the core expands more rapidly than the shell can accommodate. As the core expands outward, the brittle, non‐ductile shell experiences increasing circumferential tensile stress, ultimately leading to mechanical failure once the stress exceeds its fracture strength. Hence, the liquid metal flows outward due to internal pressure and surface tension. As a result, coalesced neighboring LMMPs form heterogeneous and continuous LMMP‐rich regions throughout the elastomer network (Figure S13, Supporting Information). Exposure to ambient oxygen causes the newly formed heterogeneous LMMP regions to re‐oxidize, forming a secondary oxide layer that is not only less uniform and more porous but also potentially thicker—especially at particle junctions. The excellent thermal stability, low thermal conductivity, and low thermal expansion coefficient of the self‐healing elastomer matrix ensure that it can withstand localized heating without thermal damage. By properly controlling the laser parameters, it is possible to precisely control the heat delivery to the LMMPs while avoiding thermally damaging the surrounding elastomer matrix (e.g., 1 repetition at 0.8 W, 50 kHz, and 400 mm s^−1^).

As shown (Figures S14 and S15, Supporting Information), the elastomer network likely undergoes quick mechanical or diffusion‐related relaxation after photothermal activation leading to a rearrangement of conductive pathways as the resistance stabilizes in ≈500 s. After more than 41 days (i.e., over 1000 h), the resistance remained completely stable across different substrates, with no degradation in the conductivity pathway or microstructure (Figures S14 and S15, Supporting Information). Furthermore, the resistance stability of photothermally activated and unencapsulated ECLMEs and/or ECLME‐HPC on Dragon Skin and EC7‐CNT substrates were evaluated after aging for 6–14 months at room temperature (Figure S16, Supporting Information). In all cases, the resistance remained stable with no signs of degradation over time (Figure S16, Supporting Information).

The resulting photothermally activated ECLME film surface changes its color as the light scatters differently (Figure S5, Supporting Information). The change of the surface morphology after photothermal activation results in higher refractive index contrast and anisotropic light scattering at the interfaces. During photothermal activation, the oxide layer may undergo changes in the surface oxide composition, thickness, or even in terms of the uniformity of the layer.^[^ ^30^, ^31^ ^]^

The morphology and microstructure of ECLME‐CNT and ECLME‐CNT prepared with hydroxypropyl cellulose (HPC) added to the LMMP dispersion differ significantly. Film surface and cross‐sectional FESEM images of ECLMEs reveal that the LMMP regions were efficiently encapsulated within the cellulose‐matrix (Figures S3 and S4, Supporting Information). As the hydroxyl group (─OH) on the cellulose backbone is polar, the interaction with the LMMPs causes additional agglomeration, thus the formation of dense LMMP‐rich regions inside the films.

The thermal stability of the elastomers and ECLMEs was evaluated using thermogravimetric analysis (TGA) and differential scanning calorimetry (DSC) over a temperature range of 30–800 °C (Figure S17, Supporting Information). The decomposition started at temperatures exceeding 384.6 and 429.9 °C for the substrate and ECLMEs as indicated by the insignificant weight loss of ≤ 3%. At temperatures exceeding 500–550 °C, the total weight loss increased to ≈7–10% due to the loss of organic components, corresponding to irreversible polymer chain scission and thermal decomposition of the siloxane backbone. However, as shown (Figure S17, Supporting Information), the addition of LMMP reduces the degradation of the elastomer matrix as the liquid metal can influence the heat distribution by acting as a thermal buffer.

In addition to the FESEM and EDS analysis (Figures S3, S4 and S7, S8, Supporting Information) and optical micrographs (Figures S5, S6, Supporting Information), surface chemistry and morphology of ECLMEs were further examined with X‐ray photoelectron (XPS) spectroscopy, atomic force microscopy (AFM) (Figure 1d,e), and attenuated total reflection Fourier transform infrared (ATR‐FTIR) spectroscopy.

As shown in the surface topography AFM images (Figure 1d), the surface roughness increased after photothermal activation. For six different regions in photothermally activated ECLMEs (prepared on AFM specimen steel disk), the surface roughnesses for the two samples were 2.268 ± 0.899 µm and 3.088 ± 1.794 µm. In comparison, for the non‐photothermally activated ECLMEs, the values were 2.902 ± 0.977 µm and 2.133 ± 0.569 µm based on two samples. The phase AFM images support the XPS analysis observations that a thin self‐healing coating forms on the coalesced LMMP regions after photothermal activation, as indicated by the decreased phase contrast (Figures S18 and S19, Supporting Information). The surface roughness of the printed ECLME layer on the self‐healing elastomer increased, which can improve adhesion by enhancing the capillary forces interacting over larger areas. Overall, the surface roughness was small enough to ensure smooth and high‐quality interfaces for soft electrical wiring and interconnections.

ATR‐FTIR spectras show that self‐healing elastomers were successfully synthesized (Figures S20–S22, Supporting Information), as evidenced by the disappearance O─H and Si‐O:B dative bonded absorption peaks at 3700, 3290, and 1340 cm^−1^, respectively, and presence of characteristic absorption peaks of Si(CH_3_)_2_‐ at 1260 cm^−1^ and Si(CH_3_)_2_‐O‐Si(CH_3_)_2_‐ at 1020–1090 cm^−1^.^[^ ^18^ ^]^ Upon mechanical damage, the elastomer matrix exhibits changes in absorption peak intensity due to supramolecular interactions (via Si‐O:B dative bonding) at 865 and 700 cm^−1^ (Figure S20, Supporting Information). As expected, FTIR spectras for all ECLMEs on EC7‐CNT were nearly featureless due to the presence of LMMP surface layer that suppresses infrared detection from the polymer phases. In contrast, distinct absorption peaks were visible in Dragon Skin elastomer possibly due to less uniform LMMP coverage. Without photothermal activation, the ECLME‐HPC on EC7‐CNT shows similar FTIR spectra in comparison to the self‐healing elastomer matrix (Figure S22, Supporting Information) due to the encapsulation of LMMPs by the cellulose matrix. After cutting, the FTIR spectra were featureless for both ECLME and ECLME‐HPC, suggesting that the liquid metal can recover and reorganize the electrically percolated network (Figures S21 and S22, Supporting Information).

The relatively thin elastomer layer covering LMMPs positioned near the surface of ECLME (Figures S18 and S19, Supporting Information) prevented the detection of metal and oxide elements (Figure 1e) due to the high surface‐sensitivity and shallow penetration depth (≈1 to 10 nm) of XPS. Therefore, we used an argon (Ar) cluster rough cleaning to remove the organic contaminants from the surface of photothermally activated ECLMEs, and to analyze metallic and oxidized phases along with surface chemical bonding (Figure 1e). The binding energies suggested significant oxidation, as evidenced by the absence of purely metallic features and the dominance of the oxide‐related peaks. The high intensities of In 3d (≈460 eV), Ga 2p_3/2_ (≈1125 eV), and Ga 2p_1/2_ (at ≈1152 eV), along with the significant atomic percentages (at.%) for mixed and oxidation states of In MN1 (10.33 at.%), Ga 3d (13.46 at.%), and Ga 3s (10.60 at.%), support the hypothesis of thick In_2_O_3_/Ga_2_O_3_ surface oxide layers present in the photothermally activated ECLMEs. The low at.% for Ga LM peaks further indicates the minimal presence of metallic Ga, reinforcing the conclusion that oxidation has occurred extensively. The hypothesis that the O─B linkages form strong coordination bonding with metal oxides was supported by the observation of possible Ga‐O‐Si bonding in the XPS analysis. For example, the O1s peak shifted to an unusually high binding energy of ≈544.5 eV, suggesting a distinct oxygen environment likely associated with Ga‐O‐Si bonding at the material interfaces. This was further supported by the observed shifts in the Ga 3d and Si 2p binding energies from the typical ranges of ≈19–20 eV and ≈99–104 eV to ≈23.0 eV and ≈107.5 eV, respectively. This indicates the presence of highly oxidized states, and the possible interaction between the surface oxides and the supramolecular elastomer matrix, facilitating the formation of stable coalesced LMMP regions with a durable surface oxide layer.

### 2.3 Electrical Conductivities on Conventional Soft Substrate

**Figure 2:**
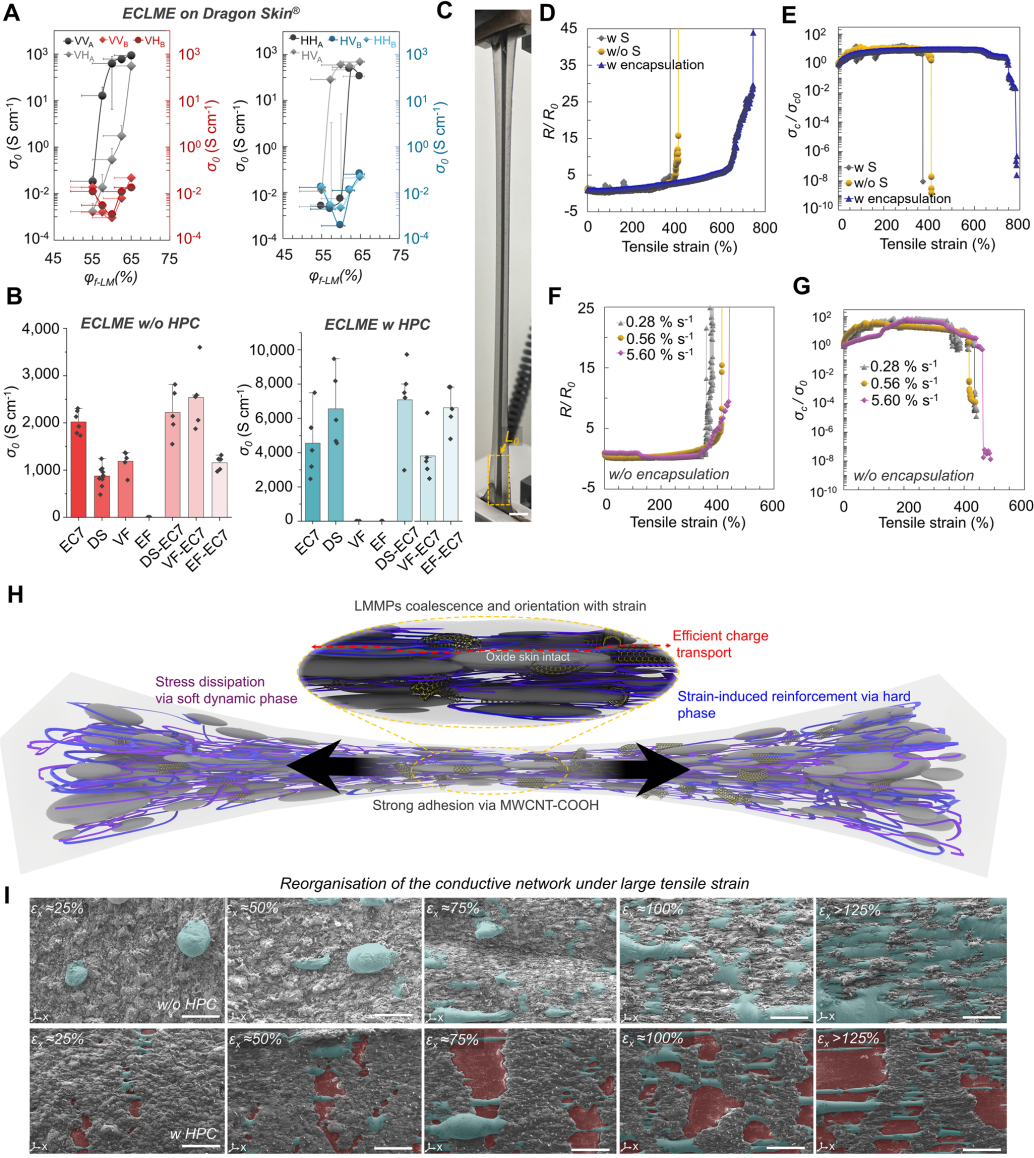
Electrical and electro‐mechanical properties. a) Electrical conductivities plotted as a function of liquid metal loading before and after photothermal activation on Dragon skin. b) Electrical conductivities of ECLME‐CNT (left) and ECLME‐HPC (right) on different substrates with and without intermediate layer (self‐healing substrate (EC7), Dragon Skin (DS), Vytaflex (VF), Ecoflex (EF)). c) Photograph of ECLME on EC7‐CNT under stretch. Scale bar 10 mm. d) Resistance ratio (i.e., *R/R_0_*) as a function of strain for ECLME on EC7‐CNT with and without surfactant (no encapsulation), and with encapsulation. e) Conductivity ratio (i.e*., σ_c_/σ_0_*) plotted as a function of tensile strain for ECLME on EC7‐CNT with and without surfactant (no encapsulation), and with encapsulation. f) *R/R_0_* as a function of tensile strain for ECLME on EC7‐CNT without encapsulation at different strain rates. g) *σ_c_/σ_0_* as a function of tensile strain at different strain rates. h) Schematic illustration of the ECLME under tensile strain. i) FESEM images of ECLME with and without hydroxypropyl cellulose under tensile strain. Scale bars 100 and 500 µm, respectively.

As the self‐healing substrate can introduce additional complexity for understanding the electrical properties of ECLMEs, we first measured the zero‐strain electrical conductivities (*σ_0_*) on the Smooth‐On Dragon Skin substrate. The *σ_0_* of ECLME‐CNTs was measured as a function of liquid metal (*φ*_f‐LM_) and carbon nanotube filler volume loadings (*φ*_f‐CNT_) over ranges of 55–65%, and 0.25–2.25%, respectively (**Figure** **2**a; Figure S24, Supporting Information). The increase of *σ_0_* as a function of *φ*_f‐LM_ can be explained by the percolation theory; *σ_0_* = *σ_LM_(p‐p_c_)^n^*, where *σ_LM_*, *p*, *p_c,_* and n are the conductivity of bulk liquid metal (≈34000 S·cm^−1^), the volume fraction of the filler, the percolation threshold, and the critical exponent, respectively.^[^ ^47^ ^]^ With *n* = 1.2–1.6, the theoretical prediction of *p_c_* ≈ 40–55% for photothermally activated ECLMEs (depending on the substrate selection) fits well with the experimental data, based on common percolation theory applicable to a wide range of electrically conductive composites. As shown (Figure S25, Supporting Information), without photothermal activation *σ_0_* gradually increases from 10^−3^ to 10^−1^ S·cm^−1^ with the LMMP loading increasing from 30 to 65 vol.% on both EC7‐CNT and Dragon Skin substrates. Interestingly, substrate selection plays a key role in photothermal activation and in the achievable percolation transition region (Figure S25, Supporting Information). The self‐healing matrix likely provides a more uniform and conformal interface with self‐leveling behavior (absent in conventional elastomers), which promotes closer inter‐droplet contact and facilitates connectivity at lower LMMP loadings after photothermal activation.

Interestingly, regardless of the *φ*_f‐LM_ (which can vary approximately in the range of 0–80%) in ECLME‐CNTs or ECLME‐HPCs, the homogenously distributed and isolated LMMPs fail to form a highly conductive network. This is mainly due to the core‐shell structure of LMMPs (with the oxide shell) and the presence of a thin self‐healing elastomer coating on their surfaces. As shown (Figure 2a), after the photothermal activation of ECLME‐CNTs on Dragon Skin substrates, *σ_0_* increased by a factor of ≈10^8^–10^11^ up to 876.8 ± 209.1 S·cm^−1^ (for *φ*_f‐CNT_ = 0.75%). For ECLME‐HPC, the increase in conductivity after photothermal activation was even greater (Figure 2b), as the HPC matrix forms an additional electrically insulating barrier coating on the surface, resulting in *σ_0_ <* 10^−6^ S·cm^−1^ before activation. Photothermally activated ECLMEs with larger *φ*_f‐CNT_ had lower *σ_0_* (< 437.9 ± 104.6 S·cm^−1^) as CNTs form high resistance interfaces. The formed secondary conductive network by CNTs can disrupt the connectivity of heterogeneous LMMP networks as *φ*_f‐CNT_ increases > 1%.

### 2.4 Directional Effects of Coating and Photothermal Activation on Performance

The blade coating and photothermal activation directions must be considered relative to uni‐ and multiaxial stretching directions, also depending on the choice of substrate (Figures S26–S32, Supporting Information). Thus, we first plotted *σ_0_* as a function of *φ*_f‐LM_ and *φ*_f‐CNT_, as well as the relative resistance (*R/R_0_*) and conductivity ratios (*σ_c_/σ_0_*) under stretching for ECLME‐CNTs on Dragon Skin substrate (Figures S26 and S27, Supporting Information). The blade coating and photothermal activation directions were chosen along the length and the width of the printed ECLME wire (as shown in Figure S23, Supporting Information).

Although no strong overall orientation of LMMPs was observed in optical and SEM imaging (Figures S3–S8, Supporting Information), blade coating induces shear forces that can lead to local density variations in LMMP‐rich regions near the substrate surface—especially seen without CNTs (Figure S3, Supporting Information). The extent of this effect appears to be substrate‐dependent. This variation significantly influences the final electrical and electro‐mechanical properties under stretching after photothermal activation, particularly when conventional, non‐self‐healing soft substrates (e.g., Dragon Skin) are used. For instance, ECLME‐CNT printed on Dragon Skin exhibited pronounced directional dependence only under stretching. When the blade‐coating direction (0°) was aligned with the strain direction, stretch‐induced microcracks more readily disrupted the LMMP domains, sharply reducing both conductivity and mechanical durability. In contrast, such direction‐dependent failure modes were not observed when using the self‐healing EC7 substrate, highlighting the importance of substrate compatibility with LMMP network reformation.

For ECLME‐HPC, better performance was achieved on Dragon Skin as LMMP regions could flow over the microcracks by filling the gaps and dynamically reconnecting the conductive pathways as HPC coating ruptured (Figures S30 and S32, S33, Supporting Information). The local density variations caused an increase in heterogeneity as LMMPs can be more densely packed in some regions. Depending on the direction of blade coating, relative to photothermal activation, the local density variations resulted in directional dependence of the electrical and electro‐mechanical properties of ECLMEs under stretching on conventional substrates. As shown (Figures S24, and S26–S29, Supporting Information), generally, when blade coating and photothermal activation directions were matched, better *σ_0_*, *R/R_0,_* and *σ_c_/σ_0_* under tensile strain were achieved for ECLME‐based conductor structures on Dragon Skin substrate. Interestingly, as *φ*_f‐CNT_ increases from 0.75% to 2.25% for ECLME‐CNTs (Figure S28, Supporting Information), the dependence of properties on directional effects from blade coating and photothermal activation decreases, possibly due to improved interfacial adhesion. For ECLMEs on self‐healing substrates, the directional dependency was not significant (Figure S29, Supporting Information) considering possible sample‐to‐sample variation.

### 2.5 Performance Enhancement by the Substrate Selection

In addition to the effect of blade coating and photothermal activation directions, the substrate material plays a significant role in achieving high performance for ECLME‐based conductor structures (Figure 2b). To study the universal applicability and printability of the ECLMEs, the performance of different soft substrate material combinations was investigated. After the photothermal activation of ECLME‐CNT on EC7, Dragon Skin, VytaFlex, and EcoFlex substrates, *σ_0_* were 2020 ± 243, 875 ± 209, 1187 ± 237, 0.014 ± 0.020 S·cm^−1^, respectively. ECLME‐CNT blade coated on EcoFlex showed poor electrical conductivity. We hypothesize this was due to the low surface energy that prevents proper wetting and adhesion at the interface. EcoFlex had also higher surface roughness which increased the heterogeneity of the blade coated ECLME‐CNTs layer (as observed in FESEM images without the EC7 intermediate layer (Figure S7, Supporting Information)).

To improve the electrical performance of ECLME, we added EC7 intermediate layer between the substrate and the blade coated ECLME‐layer. With the use of EC7 intermediate layer on Dragon Skin, VytaFlex, and Ecoflex, *σ_0_* increased to 2220 ± 508, 2530 ± 671, 1154 ± 150 S·cm^−1^, respectively. For ECLME‐HPC blade coated onto EC7, and Dragon Skin, VytaFlex and EcoFlex (with EC7 intermediate layers), *σ_0_* were ≈4549 ± 1993, 6558 ± 2186, 7081 ± 2495, 3811 ± 1477, and 6620 ± 1274 S·cm^−1^, respectively. On VytaFlex and EcoFlex substrates it was not possible to achieve highly conductive films with ECLME‐HPC without EC7 intermediate layer (Figure 2b). With EC7 intermediate layer, a highly conductive ECLME‐CNT layer could be achieved even with EcoFlex substrate (Figure 2b). FESEM images of ECLME‐CNTs with and without EC7 intermediate layers show that the film surface microstructure changes significantly (Figure S7, Supporting Information). The EC7 intermediate layer decreases the surface roughness of the printed ECLME‐CNT layers. This was particularly important for efficient photothermal activation requiring a uniform blade coated layer.

Interestingly, the use of EC7 intermediate layer improves the insensitivity to the blade coating and photothermal activation directions with ECLMEs (Figure S29, Supporting Information). As the commercial substrates (e.g., Dragon Skin and EcoFlex series platinum‐cured silicones, and VytaFlex urethane rubber) lack self‐healing functionality, any microstructural defect or discontinuity in the conductive LMMP network remains unrepaired. This influences the achievable level of conductivity with the photothermal activation and the overall working range of the conductor. As shown in (Figure S30, Supporting Information), the ECLME‐CNT on Dragon Skin easily develops microcracks at relatively small uniaxial tensile strain (< 100%), possibly due to strong strain‐induced reinforcement by the polymer chain alignment.^[^ ^48^, ^49^ ^]^ With EC7 intermediate layer on Dragon Skin (Figure S29, Supporting Information), the working range of ECLME improved up to *ε* = 170–238% (by a factor of ≈1.6 to 6.1). Similarly, the maximum conductivities (*σ_c_*) under *ε* on Dragon Skin/EC7 increased to 2860–17150 S·cm^−1^ at *ε* = 45–95% (< 2800 S·cm^−1^ without the EC7 intermediate layer) (Figure 2b; Figure S27 and S29, Supporting Information). With the use of EC7 intermediate layer, we found no microcracks in the printed ECLME‐CNT layer. This was because EC7 can both reversible break and reform bonds to dissipate stress and maintain robust adhesion to both conventional elastomer substrate and the ECLME.^[^ ^18^, ^50^ ^]^ For VytaFlex, we observed no significant differences with the addition of EC7 intermediate layer (Figure S29, Supporting Information) possibly due to a lack of strain‐induced stiffening of the substrate polymer network (Figure S33, Supporting Information), and partly due to difficulties in completely removing the air inside the VytaFlex substrate. The entrapped air limited stretching to ≈300% before the substrate fractured due to crack propagation. The maximum conductivities on VytaFlex/EC7 were approximately in the range of 3850–16320 S·cm^−1^ at *ε* ≈280%.

The self‐healing EC7 intermediate layer (with and without CNT) ensures proper wetting and adhesion while having favorable surface energy, and chemical and mechanical compatibility with ECLMEs. This facilitates the formation of continuous LMMP‐rich regions after photothermal activation. Thus, there is a reduced probability of the formation of isolated conductive regions that disrupt the conductive pathways. The hydrogen bonding, O─B bonds, and B─O─C linkages enable the intermediate layer also to act as a self‐healing buffer layer. This prevents the formation of localized stress concentrations and enhances the dynamic rearrangement of the conductive pathways, thereby delaying the potential crack formation and propagation.^[^ ^18^ ^]^

### 2.6 Stretch‐Response on Self‐Healing Substrate and Microstructural Evolution under Strain

We plotted *R/R_0_* and *σ_c_/σ_0_* for ECLME‐CNT conductor structures as a function of tensile strain on EC7 substrates with and without surfactant (Triton X‐100), and with EC7‐CNT encapsulation layer (Figure 2d,e). For the non‐encapsulated ECLMEs on EC7‐CNT, the *R/R_0_* and *σ_c_/σ_0_* increased to 1.223–1.482 and 10.731–12.452, respectively, as *ε* increased to ≈300%. The maximum conductivities in the range of ≈18000–30000 S·cm^−1^ were achieved at the *ε* = 200–300%. The maximum working range for the ECLMEs without the EC7‐CNT encapsulation was ≈*ε* = 400–450%. ECLME on EC7 or EC7‐CNT substrate with surfactant was considerably less stable. This was because the amphiphilic surfactant disrupts the adhesion of LMMPs and MWCNT‐COOH to the supramolecular elastomer matrix. For the encapsulated ECLME, the increase of *R/R_0_* was larger (1.923 at *ε* = 300%), while the *σ_c_/σ_0_* was smaller (8.174 at *ε* = 300%). The *R/R_0_* steadily increases from 1.923 to 3.962 at *ε* = 300–500%. In this case, the maximum conductivity of > 18000 S·cm^−1^ was achieved at *ε* = 500%. Under large strain, the electrical conductivities of the isotropic ECLME/EC7‐CNT elastomer conductor structures approached the bulk conductivity of gallium‐based liquid metals (≈35000 S·cm^−1^). With the use of EC7‐CNT encapsulation layer, the elastic working range of the conductor (with minimal resistance change) increased to 640%. Overall, the stretchability of the conductor with the encapsulation exceeded *ε* = 750% (*R/R_0_* < 29.468).

Near the stretch threshold of 550–650% (where resistance started to increase rapidly), visible color changes were also observed in ECLMEs (Figure S12, Supporting Information). This stretch region corresponded also to the starting point of rapid strain‐induced reinforcement of the supramolecular elastomer matrix. We hypothesized that the coalesced LMMP regions in ECLMEs start to break apart due to the rupture of the surface oxides. This results in stretch‐induced microstructural changes, where coalesced LMMP regions break into isolated LMMP droplets, influencing the refractive index contrast and producing isotropic light scattering from the material interfaces.

Stretch‐speed insensitivity is critical to ensure consistent and repeatable performance of self‐healing conductors used in soft electronic devices and circuits. Thus, the effect of stretching speed was further studied by measuring *R/R_0_* and *σ_c_/σ_0_* as a function of tensile strain at strain rates of 0.28 to 5.60% s^−1^ (Figure 2f,g). The *R/R_0_* decreased to ≈0.312–0.372 at *ε* = 200–300%. After the conductivity saturation, *σ_c_/σ_0_*, decreased to less than 0.524 at *ε* > 380%. Similarly, *R/R_0_* increased up to 15.49–35.32 at *ε* = 383–416% for 0.28–5.60% s^−1^. Overall, the electro‐mechanical behavior of ECLMEs was insensitive to strain rates measured up to 5.60% s^−1^ due to similarities of *R/R_0_* and *σ_c_/σ_0_*, and by the achievable working ranges.

The stretching‐induced microstructural change was schematically illustrated in Figure 2h. At zero strain, the tortuosity of the LMMP network limits possible achievable conductivity by forcing more scattered conductive pathways. In this case, the movement of charge carriers is restricted by the increased non‐linear paths, and the existing disorder in the conductive network. It is well known that the stretching of liquid columns would be limited by the Plateau–Rayleigh instability, where cylinder liquid bridges break apart once elongated beyond the critical length limit.^[^ ^51^ ^]^ The instability occurs when liquid columns pinch off into separated droplets once their aspect ratio exceeds the limit that the surface oxide can withstand, followed by the need to minimize the surface area.

However, unlike in freestanding liquid columns, the stable surface oxide and adhesion at the material interfaces constrain the heterogeneous LMMP regions within the supramolecular elastomer network, which can reversibly break and reform bonds to redistribute mechanical stress.^[^ ^52^ ^]^ This can significantly delay the capillary instability (Figure 2i) during the stretch‐induced alignment of polymer chains. The stable surface oxide allows the rearrangement conductive network under stretch as the LMMP‐rich regions have the freedom to align and grow along the stretching direction (Figure 2h,i). The ductility of the surface oxide and the interfacial adhesion resist the capillary‐driven contraction that would otherwise break the surface oxide and therefore result in the disruption of the dynamically reorganized conductive pathways that reversibly align with the stretching direction. The porosity of the surface oxide allows mechanical interlocking with the surrounding polymer, while still allowing sufficient flow and reorganization of LMMPs regions.

With the significant decrease in the cross‐sectional area, the conduction pathways form parallel to each other along the stretch direction due to significant polymer chain alignment (Figure 2i). This further increases the conductivity of ECLME as the elongated and parallel‐connected LMMP regions have strongly adhered to the surrounding elastomer matrix. The formation of elongated LMMP columns/regions reduces the number of particle‐to‐particle junctions resulting in lower contact resistance inside the 3D supramolecular elastomer network. Thus, electrons travel through fewer high‐resistance junctions leading to significant stretch‐induced conductivity improvement. However, as shown later, the conductivities with ECLME plateaus at the microscopic level as the bulk liquid metal sets the upper bound for the maximum achievable conductivity of the stretched ECLME layer. In the case of ECLME‐HPC, the encapsulation by the HPC matrix can irreversibly break under stretch. This allows a flow of the heterogeneous LMMP regions over the formed microcracks in the surface of the substrate resulting in the formation of a conductive network with a stronger stretch‐induced alignment (Figure 2i).

### 2.7 Tensile and Self‐Healing Properties

We measured the tensile stress–strain curves for EC7‐CNTs as a function of *φ*_f‐CNT_ (from 0.025 to 2.25% (Figure S34, Supporting Information)), and volume loading of boron oxide nanoparticles (B_2_O_3_ NPs; *φ*_f‐B2O3_) (from 0.05 to 0.25% (Figure S35, Supporting Information)) for finding the optimal balance between tensile and self‐healing properties, microstructural stability for the self‐healing substrate (Table S3 and Figures S36 and S37, Supporting Information). For pristine EC7‐CNTs (*φ*_f‐B2O3_ = 1.0%), Young's modulus (*E*), elongation at break (*ε*_break_), and toughness (*U_T_*) increases non‐linearly from 0.477 ± 0.032 MPa to 1.019 ± 0.037 MPa, from 1187 ± 161% to 1920 ± 36%, from 18.89 ± 5.22 MJ m^−3^ to 100.61 ± 6.73 MJ m^−3^, respectively, with the *φ*_f‐CNT_ increasing from 0.25 to 2.25% (Table S3 and Figure S37, Supporting Information). The maximum *ε*_break_ and *U_T_* were up to 2091 ± 122%–2156 ± 252%, and 94.58 ± 30.20 MJ m^−3^–118.02 ± 25.63 MJ m^−3^, respectively, with *φ*_f‐CNT_ = 1.25–1.75% (Table S3, Supporting Information). Although the toughness of autonomously self‐healing materials with polyurethane‐based elastomers has reached up to 282.7 MJ m^−3^, the toughness achievable with EC7‐CNT, to the best of our knowledge, is one the highest ever reported for polydimethylsiloxane‐based elastomers (Table S4, Supporting Information).

The remarkable improvement in the tensile properties can be attributed to the interaction and synergistic effect between MWCNT‐COOH and boron oxide (B_2_O_3_) nanoparticles (NPs) present in supramolecular elastomer network (Figure S38, Supporting Information). Interestingly, with 0.5 wt.% B_2_O_3_ NP content, the increase of CNT loading does not lead to any noticeable reinforcement or toughening, and the elastomer network becomes vulnerable to rupture under large stretches (Figure S38, Supporting Information). At 1 wt.% B_2_O_3_ NP content, the synergistic interaction between COOH‐CNTs and B_2_O_3_ NPs leads to the formation of a reinforced, highly entangled elastic network that can efficiently transfer load and dissipate strain at material interfaces through reversible hydrogen bonding (Figure S34, Supporting Information).

**Figure 3:**
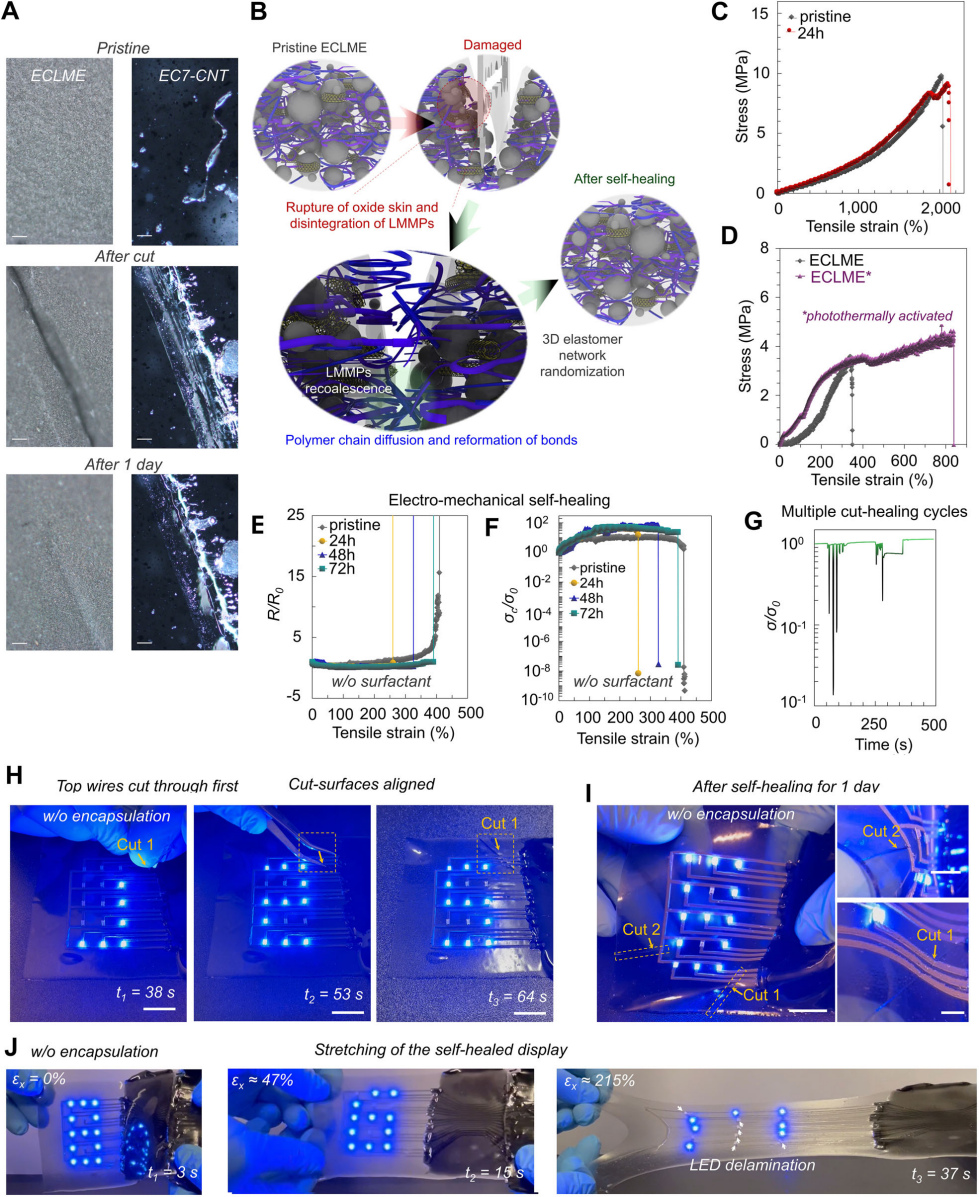
Self‐healing properties. a) Optical micrographs of the self‐healing process in ECLME (left), and EC7‐CNT (right). Scale bars 150 µm. b) Schematic illustration of the self‐healing process in ECLME. c) Tensile stress–strain curves for pristine and self‐healed EC7‐CNT substrate. d) Tensile stress–strain curves for freestanding ECLME films before and after self‐healing. e,f) *R/R_0_* and *σ_c_/σ_0_* as a function of tensile strain for ECLME on EC7‐CNT before and after self‐healing. g) Conductivity ratio measured as a function of time when ECLME is cut repeatably. h,i) Photographs of ECLME‐based self‐healing LED‐display without encapsulation while cut, aligning cut‐surfaces, and after self‐healing. Scale bars 20 mm. j) Photographs of ECLME‐based self‐healing LED‐display without encapsulation after self‐healing when uniaxially stretched.

As shown in (Table S3, Supporting Information), the self‐healing efficiencies (calculated from *U_T_*) for EC7‐CNTs after 24 h were in the range of 94.0 ± 37.6%–100.7 ± 16.5%, 97.0 ± 8.5%–89.4 ± 0.9%, and 41.4 ± 0.4%–17.4 ± 1.1% with *φ*_f‐CNT_ = 0.025–0.25%, 0.75–1.25%, and 1.75–2.25%, respectively. As shown (**Figure** **3**a), EC7‐CNT films can show a visible cut on the surface after self‐healing even when tensile properties are completely recovered. This is because the self‐healing kinetics at the surface of the film are much slower than in the bulk. At large *φ*_f‐CNT_ (> 1.5%) the complete self‐healing of EC7‐CNT takes several days in ambient conditions after the cut‐surface alignment (instead of one h with EC7). The self‐healing kinetics showed power‐law dependency (Figure S39, Supporting Information) as the physical self‐healing processes (polymer chain diffusion, chain re‐entanglement, capillary flow, etc.) are rate limited, and the surface tension‐driven flow into voids decay over time due to viscous dissipation and geometric constraints. As shown (Figures S38 and S39, Supporting Information), the self‐healing kinetics of the elastomer matrix are reduced with increasing *φ*_f‐CNT_ loading, as the restricted mobility of the polymer chains slows down their diffusion. With fixed *φ*_f‐CNT_ and even lower concentrations of B_2_O_3_ NPs, self‐healing becomes significantly slower without any improvement of microstructural stability (Table S1 and Figure S36, Supporting Information).

To measure the tensile properties of ECLMEs, freestanding films were made by blade coating ECLME‐CTNs onto VytaFlex. Then, the non‐encapsulated ECLME‐CNT/VytaFlex structures were uniaxially stretched (> *ε* = 300%). After stretch‐release and waiting for weeks (after the complete stress relaxation), the freestanding ECLME‐CNT films could be achieved by peeling the printed layer from the VytaFlex substrate with tweezers. We found that the non‐photothermally patterned ECLME films had *E*, *ε*_break_, and *U_T_* of 1.099 ± 0.100 MPa, 296.2 ± 33.0%, and 3.466 ± 0.823 MJ m^−3^ (Table S5, Supporting Information). With the photothermal activation of ECLME, *ε*_break_ and *U_T_* remarkably increase to ≈837% and 25.88 MJ m^−3^, respectively (Figure 3d). The *ε*_break_ with photothermally activated ECLMEs further supports the electro‐mechanical measurement, where the resistance increased significantly at *ε* ≈800%. Overall, the heterogeneous LMMP‐rich regions, along with potentially thicker and more uniform surface oxide layer prevent the crack propagation by efficiently redistributing stress within ECLME.

### 2.8 Self‐Healing Mechanisms in ECLMEs

The self‐healing in ECLME‐CNTs and ECLME‐HPCs were closely related to a surface‐tension driven self‐healing and the entropic recovery with consideration of also the high surface tension (*γ*) of gallium‐based liquid metals (in the range of ≈533–713 mN m^−1^).^[^ ^53^, ^54^, ^55^ ^]^ Upon mechanically induced damage, the confined LMMPs within the 3D supramolecular elastomer network became exposed at the cut‐surface. As the induced mechanical force overcomes the surface tension, the exposed LMMP regions are disintegrated into smaller droplets (Figure S6, Supporting Information), and the polymer chains reversibly dissociate at the damage site. After the initial damage, the material system tends to minimize the free energy of the high‐order state by surface tension driven self‐healing after the wetting by the polymer occurs. As the Laplace pressure (*ΔP*) across particle interface is smallest with larger particles (*ΔP = 2γ/r*, where *r* is a droplet radius),^[^ ^56^ ^]^ the surface tension drives the re‐coalescence of smaller LMMPs in ECLME layer to form larger droplets. This provides the smallest surface area (*A*) for the given volume and allows reducing the surface energy (as *E_s_ = γA*). As the surface tension reduces with the size of the damaged area decreasing, a transition to entropy‐driven self‐healing may occur.^[^ ^18^, ^57^, ^58^ ^]^ During the shape recovery of ECLME, the surface oxide reforms at the re‐coalesced LMMP regions, while the dynamic bonds diffuse over time to their equilibrium distances. This reduces the total energy of the system and allows transition into a more disordered state that is the thermodynamically favorable equilibrium.

We hypothesize that the addition of CNT to the ECLMEs enables the growth of thicker oxide that strengthens the LMMP regions (confined inside the 3D elastomer network). This oxide layer, while porous, may play a key role in enabling exceptional strain‐insensitivity. The COOH‐ group can chemically interact with the surface oxides (Ga_2_O_3_, In_2_O_3_) through coordination bonding resulting in an anchoring‐effect. Thus, allowing the oxides to grow more densely without external disruptions. A thicker, porous oxide shell formed by the photothermal activation of ECLME likely contributes to a mechanically stabilized interfacial network and improved electrical connectivity under strain. Additionally, the porous surface oxide layer may facilitate improved wetting and controllable flow of LMMPs, which are crucial for self‐healing and adhesive performance, and for minimizing LMMP leakage under deformation. Although the surface oxide is known to be critical for the self‐healing process, altogether it is still a physical barrier for direct particle‐to‐particle coalescence and prevents the free flow of liquid metal if thick and insufficiently porous. Thus, the overall performance of dynamically reorganizing self‐healing networks utilizing LMMPs as electric fillers is likely closely tied to achieving an optimal oxide thickness—sufficiently thick and porous enough to promote strong bonding and electrical continuity, yet not so dense to hinder the wetting dynamics or intrinsic flow of LMMP regions required for self‐healing and reconfiguration. Hence, depending on how the cut was induced, it may be difficult to overcome the oxide barrier without external activation (such as cut‐surface alignment). We further hypothesized that thicker oxide results in a stronger confinement within the 3D elastomer network, thereby more easily disrupting the reorganization of LMMP network near the cut‐surface during spontaneous self‐healing in multilayered assemblies having at least three layers.

In ECLME‐HPC, the addition of HPC forms a soft coating onto the heterogeneous LMMP regions which reduces both the surface oxide cohesion and adhesion at the material interfaces. When mechanically damaged, the soft coating is easily permanently ruptured. The rupture of HPC‐based coating is followed by an effective distribution and capillary‐driven reorganization of the network as LMMPs can easily flow, coalescence, and form larger LMMP regions near the cut‐surfaces. As HPC acts as a semi‐permeable membrane it can also modulate the oxygen permeability, and limit the oxide growth and cohesion that would otherwise disrupt the mobility of liquid metal.

### 2.9 Self‐Healing of ECLME‐Based Functional Structures

The self‐healing properties of ECLME‐based functional structures on EC7‐CNT substrates (Figure 3e,j; Figures S9–S11 and Movies S1–S3, Supporting Information) were investigated. For ECLME‐based conductor structures on EC7‐CNT substrates, we also measured both *R/R_0_* and *σ_c_/σ_0_* before and after the cut‐surface self‐healing, and with and without uniaxial stretching.

Self‐healing of coated ECLME‐CNT‐based spaced line‐patterns is shown in Figure S6, and Figures S9–S11, Supporting Information. As shown, cut surface alignment is not necessarily required for initiating self‐healing, but longer self‐healing times may be required to achieve complete recovery. After self‐healing for 24 h (without cut‐surface alignment), the spaced line‐patterns can be already stretched to > 300% without fracture (Figure S11, Supporting Information). The photographs and optical micrographs for the spaced line‐patterns at different line widths (50–1000 µm) and gaps (100–1000 µm) indicate that photothermal activation significantly improves the binding of LMMPs to the ECLMEs and leakage resistance due to improved oxide cohesion and possibly formation of thicker surface oxide layer (Figures S5,  S6, and S9–S11, Supporting Information). For non‐photothermally activated ECLMEs, we found that the degree of leaking was dependent on the physical footprint of the printed pattern and the surface area of the damage. As the printed non‐photothermally activated ECLMEs were bisected with a razor, LMMPs were squeezed out near the cut‐location due to induced pressure gradient. The squeezed LMMPs tended to form larger droplets on the surface of the film near the cut‐location rather than fully detaching out to the sides. In comparison, with the photothermally activated ECLMEs, the heterogeneous LMMP‐rich regions were strongly adhered to the supramolecular elastomer resulting in no visible leaking which would otherwise be an issue in soft electronic circuits due to possible electrical shorting.

We measured *R/R_0_* and *σ_c_/σ_0_* as a function of tensile strain for pristine and self‐healed ECLME‐CNT/EC7‐CNT conductor structures without encapsulation (Figure 3e,f). With pristine conductor *R/R_0_* increased from 0.438 to 1.434 at *ε* = 0–300%, while the working range was up to 406.8% with *R/R_0_* of 8.09. The maximum *σ_c_* of >23600 S·cm^−1^ was achieved at *ε* ≈ 200% before saturated and followed by a slow decay. Then, we tested the self‐healing of ECLME‐based interconnections on EC7‐CNT substrate by completely bisecting the sample with a razor after which it was left to self‐heal without any cut‐surface alignment. After self‐healing for 24, 48, and 72 h in ambient conditions, the ECLME‐based interconnections fractured at *ε* = 259.0, 323.7, and 389.7%, respectively. Interestingly, after self‐healing, we found a significant strain‐induced conductivity improvement resulting only in negative resistance change at the complete working range of the conductor. With the self‐healed conductors, *R/R_0_* decreased to 0.138–0.205 at *ε* = 100%, before further increasing up to < 0.993 at the breaking strain (*ε* ≈ 259–390%). Without cut‐surface alignment, the *σ_0_* remained approximately at a range of 700–1100 S·cm^−1^ in the relaxed state. Hence, the application of any stretching resulted in immediate dynamic reconstruction of the conductive pathways and an even more drastic improvement of the *σ_c_*. At *ε* ≈ 200%, the conductivities of self‐healed conductors were near the bulk conductivity of pure eGaIn, and significantly better than with pristine conductors. The decrease of the area under *R/R_0_* and *σ_c_* / *σ_0_* versus *ε* curve and similar *ε_b_* implies that the self‐healed conductor performs better than the pristine one (Figure 3e,f). Thus, the calculated electro‐mechanical self‐healing efficiency of ECLME after self‐healing for 72 h was more than 100%.

To also demonstrate the excellent self‐healing of ECLME‐HPC, *σ_c_/σ_0_* was also measured as a function of time when the conductor was cut repeatedly 12 times without any cut‐surface alignment (Figure 3g). During cutting, *σ_c_/σ_0_* decreased to 0.138, and after the removal of the razor *σ_c_/σ_0_* recovered immediately to ≈1.0. The first three cuts were followed by nine more cuts (after 94 s since starting the measurement). After twelve cuts along the length of the ECLME‐HPC, the conductor was completely mechanically disconnected from one location. After the cut‐surface alignment on the disconnect location, the healed conductivity (*σ*) surpassed the original conductivity (*σ_0_*) of a pristine conductor (*σ/σ_0_* ≈ 1.14). As shown (Figure 3g), the self‐healing efficiency for the electrical conductivity exceeded 100% after multiple cutting‐healing cycles completed in under 400 s.

The self‐healing functionality was further demonstrated in practice with an ECLME‐CNT‐based pixel‐driven LED‐display (Figure 3h,j). Two transverse cuts were made through the ECLME‐based interactions and the EC7‐CNT substrate. First, by completely cutting through three wires (Figure 3h) at *t_1_* = 38 s (Movie S1, Supporting Information). The second cut was made through another single wire (at *t_4_* = 125 s), followed shortly after the alignment of the first cut‐surfaces (at *t_3_* = 64 s). During the cut‐surface alignment, the sharp tip can puncture the film surface, but we found no significant leaking of LMMPs (Figure 3i; Movie S2, Supporting Information). The electrical conductivity of ECLME interconnection recovers immediately with the cut‐surface alignment (Figure 3h; Movie S1, Supporting Information). After the cut‐surface alignment and self‐healing for 24 h in ambient conditions, the cuts were still visible on the surface of the ECLME‐interconnection and substrate (Figure 3i; Movie S2, Supporting Information).

After self‐healing for 24 h, the ECLME‐CNT‐based LED display structure was uniaxially stretched (Figure 3j; Movie S3, Supporting Information). Without the EC7 or EC7‐CNT encapsulation layers, the display structure was completely functional up to *ε* > 47% (at *t_1_* = 38 s). At significantly larger tensile strain, the directly bonded SMD LEDs on the surface of ECLME started to partially delaminate due to stretch‐induced rotation. When the self‐healed display structure was stretched up to 215% at *t_3_* = 37 s (Movie S3, Supporting Information), 8 of the 15 LEDs were still completely functional. Some of the LEDs were partially dislocated without debonding by the stretch‐induced rotation due to the absence of an encapsulation layer (Figure 3i). As shown in Figure 3j, one of the three LEDs connected to the wire that was cut was still completely functional at *ε* ≈ 215%. Thus, the display's functionality loss under the stretching is mainly related to the dislocation of SMD‐LEDs without additional design considerations to improve localized bonding or the use of an encapsulation layer (as illustrated in Figure S41, Supporting Information). Since the LEDs were manually placed onto the ECLME interconnections using tweezers, the bonding strength varied between components. As a result, some LEDs were inadequately bonded, leading to partial loss of display functionality under larger strains. Given the pressure‐sensitive adhesive nature of the material, both the applied force during initial bonding and the waiting time afterward are critical to be optimized in future work. Allowing sufficient time to pass after placement of the component could enhance interfacial bonding through the material's self‐healing properties, thereby significantly improving the durability of the stretchable device.

### 2.10 ECLME‐Based Display and Battery‐Integrated LED Demonstrator

**Figure 4:**
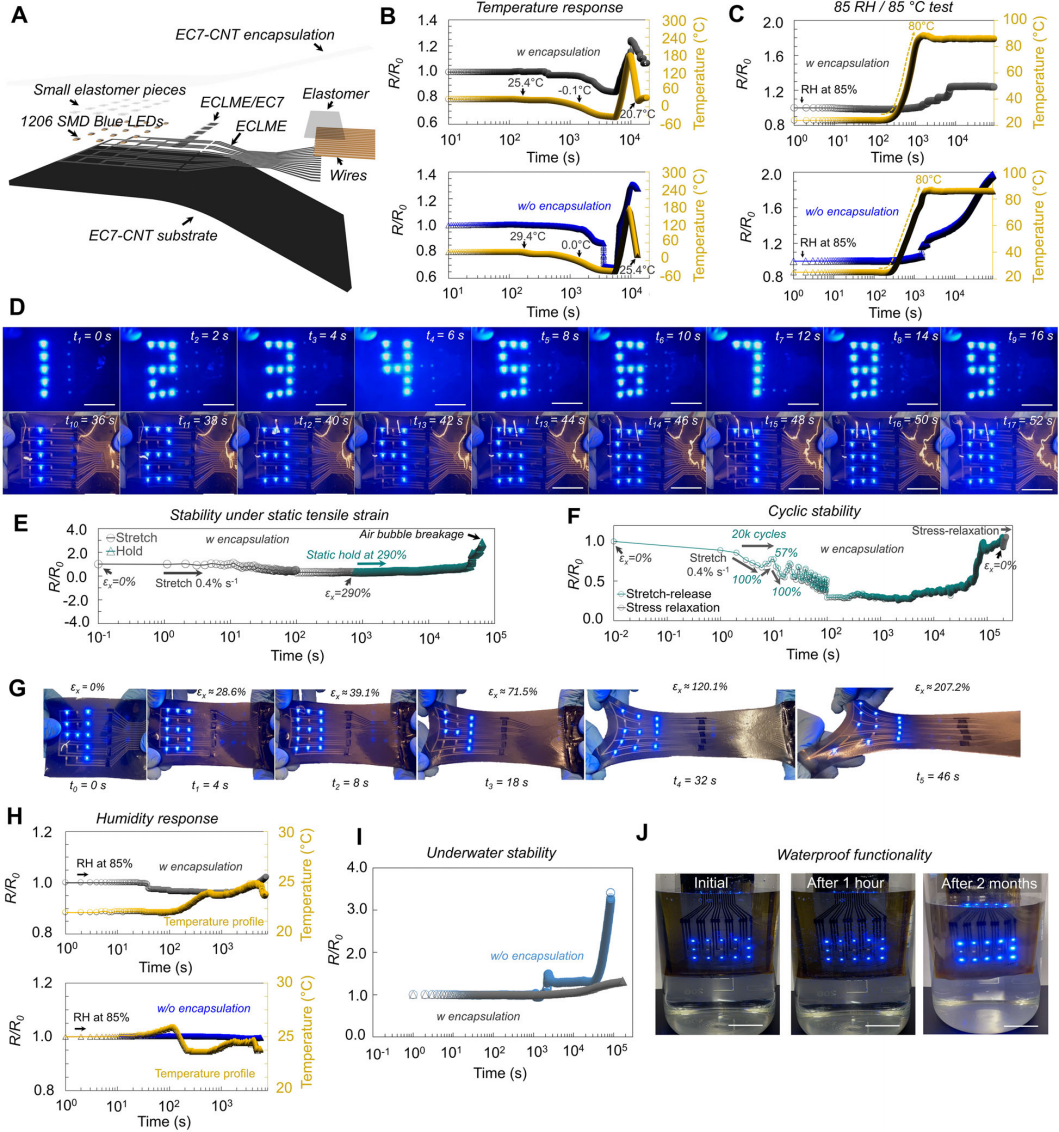
ECLME interconnections and display. a) Schematic illustration of the display. b) *R/R_0_* plotted as a function of temperature for ECLME on EC7‐CNT with and without encapsulation. c) 85% RH/85 °C test for the ECLME on EC7‐CNT with and without encapsulation. d) Photographs of ECLME‐based self‐healing LED‐display with number counting in dark and with ambient light. Scale bars 30 mm. e) *R/R_0_* plotted as a function of time under static uniaxial stretching to 290% for ECLME on EC7‐CNT. f) Stability of *R/R_0_* as a function of time during 20000 stretch‐release cycles for ECLME on EC7‐CNT. g) Photographs of the display under tensile. h) *R/R_0_* as a function of time for ECLME on EC7‐CNT with and without encapsulation when relative humidity increases from 0% to 85%. i) *R/R_0_* as a function of time when ECLME on EC7‐CNT is placed underwater. j) Photographs of the encapsulated underwater display. Scale bars 30 mm.

A complete structure, fabrication, and working principle of self‐healing and stretchable ECLME‐based 3 by 5 pixel‐driven LED‐display is shown in **Figure** **4**a; Figures S40–S42, Supporting Information. The performance and functionality of ECLME‐based interconnections and the display were further evaluated under dynamic and static tensile strains, temperature and humidity, and underwater (Figure 4b–j).

The electrical stability of ECLME‐based interconnections for the display and soft electronics integration were first investigated by measuring *R/R_0_* as a function of temperature from ‐40 to 180 °C (Figure 4b), relative humidity from 0 to 85 RH% (Figure 4c) and with 85 RH%/85 °C test.

ECLME‐based interconnections were extremely stable at highly humid conditions with relatively constant temperatures (in the range of ≈22.1–25.9 °C) as *R/R_0_* were 1.021 and 0.995 at 85 RH% with and without the encapsulation, respectively. We hypothesized that small swelling of the encapsulation layer at the high humid conditions may induce a localized mechanical strain to the printed ECLME layer, and therefore the resistance fluctuated a bit. For encapsulated and non‐encapsulated ECLME‐based interconnections, *R/R_0_* first decreased to 0.847 and 0.691, respectively, as temperature decreased to ‐40 °C due to cooling induced contraction. Upon heating the thermal stress builds up gradually, and the calculated temperature coefficient of resistances (TCRs) were ≈1.79·10^−3^ °C^−1^, and 2.79·10^−3^ °C^−1^, respectively. TRCs for ECLMEs are within the range of bulk metals. Without encapsulation, the LMMP regions have more freedom to expand and contract within the elastomer network resulting in higher temperature‐dependency of the resistance. Upon cooling to room temperature, the *R/R_0_* immediately recovered to values of 1.067 and 1.275, respectively, before further recovery.

The achieved TRCs suggest that the effect of the temperature‐dependent *γ* can be neglected without deformation (*γ(T)≈ γ_0_− k(T−T_0_)/V_m_^2/3^*, where V_m_ is molar volume, *T_0_* is a room temperature, and k is Eötvös’ coefficient).^[^ ^59^ ^]^ However, γ of gallium‐based liquid metals is known to increase by ≈3.87–8.0 mN m^−1^ K^−1^ up to ≈230 K with the temperature decreasing.^[^ ^53^, ^60^ ^]^ Thus, we hypothesized that the temperature‐dependency of *γ* would affect the dynamic reorganization of the conductive pathways of ECLMEs under strain at different temperatures, but it would require further investigation in the future.

To study further the stability of ECLME‐based interconnection, we first applied a dynamic stretching at 0.4% s^−1^ strain rate to *ε* = 290%. The initial stretch was followed by a static hold for > 65000 s (i.e., > 18 h) at *ε* = 290%. After the initial stretch, *R/R_0_* decreased to 0.741 at *ε* = 290%, before increasing to 0.887 after >11 h at *ε* = 290%. After >12.6 h at *ε* = 290%, we observed a gradual increase of *R/R_0_* from 0.887 to 1.433, and eventually to 1.880 after the conductor was at *ε* = 290% for ≈18 h. The gradual increase of *R/R_0_* related to air bubble breakage resulted in a slow crack propagation inside the EC7‐CNT substrate. We hypothesized that the crack propagation under the static strain was faster than the substrates’ ability to heal the microstructural damage, hence conductor eventually fractured.

Continuously dynamic stretch‐release cycling without a stress relaxation in between each cycle increases the likelihood of microstructural fatigue with the conductive pathways. Thus, we applied cyclic stretching at 0.4% s^−1^ strain rate between 20000 cycles at *ε* = 57–100% for the total duration of ≈55 h. As shown in Figure 4f *R/R_0_* initially decreases to 0.254 after 200 cycles due to the dynamic rearrangement of LMMP network under strain. *R/R_0_* was still less than 0.94 after the cyclic stability testing of 20000 cycles. The increase of *R/R_0_* after 89000 s was due to a small gradual increase of residual strain of the EC7‐CNT substrate that reduces the deformation that ECLME layer experiences for each consecutive cycle. Due to negative resistance change under smaller strain (< 200%), smaller deformation increases the resistance of ECLME. During stress‐relaxation after cyclic testing (at > 200000 s), the *R/R_0_* increases to 1.044 at *ε* = 0% (less than 4.5% change before further self‐healing). Following the cyclic testing, the unencapsulated sample (shown in Figure 4f) was stored for one year at room temperature and then remeasured over 1100 stretch‐release cycles (at *ε* = 100%). The sample maintained similar electro‐mechanical performance without any signs of aging‐related degradation of the conductive pathways (Figure S43, Supporting Information). Similarly, a stable response was observed for another photothermally activated sample aged for six months at room temperature which was subjected to 900 stretch‐release cycles at *ε* = 100% (Figure S43, Supporting Information). Thus, overall, the ECLME‐interconnection on EC7‐CNT substrates shows exceptionally good mechanical and electrical resilience, and stability in comparison to stretchable conductors with metallic nanofillers and liquid metal inclusions without self‐healing functionality.

After studying the stability of ECLME‐based interconnections, we evaluated the functionality of self‐healing, and stretchable pixel‐driven LED‐display under uniaxial stretching. The display is powered and controlled through an external IDE Arduino board (Figure S41, Supporting Information) which was interfaced with the display. The 3 × 5 LED matrix is formed by integrating 15 SMD‐LEDs into ECLME‐based interconnections. As illustrated, the surface‐mountable components are pressed against the surface of ECLME‐interconnection (Figure S40, Supporting Information) to form stable electrical and mechanical bonds. Upon pressing, the thin self‐healing elastomer coating on the surface undergoes a reversible rupture, and the localized deformation leads to the formation of electrical contact between the component electrode and liquid metal core due to the discontinuity and porosity of the surface oxides. Over time, autonomous self‐healing functionality reinforces the mechanical bond as the initial adhesion is established through polymer wetting and adhesive interactions activated by pressure. The good leaking resistance of ECLME prevents spreading and unintended formation of conductive pathways underneath the component that could otherwise short circuit the display.

Microcontroller‐based electronics allow controlling the displays in real‐time as shown in Figure 4g and Movies S4–S6, Supporting Information. Under uniaxial stretching, the encapsulated display works properly, and it could maintain the original function up *ε* = 207% (Figure 4g; Movies S6, Supporting Information). The thin encapsulation layer significantly enhances the adhesion of SMD‐LEDs to the ECLME interconnections, potentially due to the additional force applied to the component surface, which may strengthen the bond. However, further investigation into the pressure‐sensitive bonding mechanism specifically at the conductor surface‐component interface is needed to fully optimize the electro‐mechanical adhesion between the surface‐mountable components and ECLME interconnections. Improving this bonding strength is a key next step toward enhancing the performance of the stretchable, self‐healing display—particularly before advancing pixel density and device functionality.

The underwater environment poses a significant risk to the functionality of electronics as degradation can rapidly occur through water ingress by the development of leakage currents, capacitive or resistive changes, or formation of short circuits. Hence, we first evaluated the underwater stability of the ECLME‐based interconnection with and without encapsulation by measuring *R/R_0_* as a function of time after being fully immersed underwater (Figure 4i). With and without encapsulation, *R/R_0_* gradually increased up to ≈1.31 and 3.29 after > 47 h and 23.8 h underwater immersions, respectively (Figure 4i). In comparison, ECLME‐based interconnections on Dragon Skin and VytaFlex substrate were significantly more unstable underwater as *R/R_0_* increased to 4.34–6.34 only after 24.7 h of underwater immersion (Figure S41, Supporting Information). This was likely due to the issues with the underwater adhesion between ECLME and the substrate without a compatible intermediate layer and encapsulation layer. Thus, the stability of printed ECLME in underwater conditions was also significantly influenced by the substrate material (Figure 4i and Figure S44, Supporting Information).

To further highlight the waterproofing functionality and robustness of the integration, the functionality of the display was evaluated in underwater conditions (in deionized water) (Figure 4j; Figures S45–S46, Movies S7 and S8, Supporting Information). Interestingly, without structural design considerations for the underwater adhesion, the bonding at the material interfaces failed in ≈30 min after the display was completely immersed underwater (Figure S44, Supporting Information). With the addition of EC7‐CNT adhesives to the SMD‐LEDs (as illustrated in FigureS40, Supporting Information), the display was functioning properly even after a long‐term underwater immersion (> 2 months) without any signs of degradation (Figure 4j; Movie S7, Supporting Information). Although we observed a small increase of resistance in ECLME‐interconnection when kept underwater (Figure 4i), the brightness of SMD‐LEDs increased over time (Figure 4j). We hypothesized that the refractive index of water could change over time, which would increase the perceived brightness of the display due to changes in light propagation and scattering. To the best of our knowledge, this is the first proof‐of‐concept of a stretchable and self‐healing pixel‐driven display that is highly stable underwater for the longer term (Table S6, Supporting Information). In future work, we aim to improve the initial proof‐of‐concept design by focusing on understanding the pressure‐sensitive adhesive properties, improving the pixel density, and enhancing the functionality of the display by integrating control electronics and battery into the same self‐healing substrate.

To further highlight the self‐healing functionality, we also fabricated a simplistic ECLME‐based battery‐integrated LED device (Figures S47–S50, Supporting Information). 3 V coin‐cell lithium battery and three SMD‐LEDs were integrated into a multilayered structure consisting of ECLME‐based interconnections and electrodes. The distinct structures were self‐bonded together via self‐healing, and a completely functional device was formed (Figure S38, Supporting Information). As shown in (Movie S9, Supporting Information), under extremely localized deformations (near the component placement), the device can fracture due to crack propagation. Upon rupture by stretch, we found no leaking of LMMPs.

As shown in (Figure S50, Supporting Information), self‐healing functionality also can allow on‐demand replacement of broken soft electronic parts in hybrid electronic devices via self‐healing. For example, we demonstrated that a small area can be replaced from the device (e.g., switching blue to green SMD‐LEDs) by completely removing the original part and adding a new replacement part to restore the functionality of the device via self‐healing. After the initial alignment, the device was electrically completely functional before further self‐healing to restore the original mechanical integrity under stretch.

## 3 Conclusion

In summary, we present an electrically conductive liquid metal elastomer (ECLME) composite ink featuring exceptional electro‐mechanical properties, autonomous self‐healing, and inherent pressure‐sensitive adhesive behavior for direct bonding of surface‐mountable components onto conductor surfaces. ECLME enables high resolution patterning (< 50 µm) via printed electronics manufacturing methods while also streamlining hybrid electronics fabrication and enhancing durability. Photothermal activation induces the formation of a stable conductive network, embedding heterogeneous liquid metal microparticle‐rich/functionalized multiwalled carbon nanotube regions within a dynamic 3D supramolecular elastomer matrix with reversible oxygen‐boron and hydrogen bonding. As a result, ECLME exhibits high conductivity (> 20000 S·cm^−1^ under strain), ultralow strain sensitivity (*R/R_0_* < 3.95 up to 500%), and an elastic working range exceeding >700%. Furthermore, ECLME‐based interconnections maintain stable performance across a wide temperature range, under high humidity and underwater conditions, and during both static and dynamic stretching. To demonstrate, we fabricated a highly stretchable, self‐healing 3 × 5 pixel display with waterproof functionality using an easily scalable blade coating and lamination process that leverages autonomous self‐healing for self‐bonding. ECLME presents a significant advancement for multifunctional, printable, self‐healing, stretchable soft electronics, enabling the realization of electrical wiring, interconnections, electrodes, RF transmission lines, tunable resonators, and frequency‐selective surfaces.

## 4 Experimental Section

### Materials

Gallium and Indium (with purities of 99.99% and 99.995%, respectively), were purchased from Smart‐elements GmbH. MWCNT‐COOH was purchased from Cheap tubes Inc. Hydroxyl terminated poly(dimethylsiloxane) (PDMS‐OH) with kinematic viscosity of 18 000 to 22 000 cSt, hydroxypropyl cellulose (HPC) with molecular weight of ≈100 000, isopropanol (≥ 99.5%), and TritonTM X‐100 (laboratory grade) were purchased from Sigma‐Aldrich. Boron oxide nanoparticles (B_2_O_3_ NPs, *d_50_* = 80 nm) were purchased from SkySpring Nanomaterials Inc. Dimethylvinyl‐terminated dimethylsiloxane‐based elastomer was purchased from Sil‐mid Limited. Smooth‐On Dragon Skin 10 Medium, VytaFlex 20, and EcoFlex 00–20 elastomers were purchased from FormX. All materials were used as received.

### Synthesis of Liquid Metal Microparticles

Bulk liquid metal was formed from a mixture of gallium (Ga) and indium (In) at Ga to In weight ratio of 75.5:24.5 (eGa_75.5_In_24.5_). The bulk metals were mixed by heating the bulk metals at 120–150 °C for at least 10 min.

23.90 grams of eGa_75.5_In_24.5_ and 2.5 grams of IPA were used to form liquid metal microparticles (LMMPs) with probe ultrasonication (Hielscher UP100H). The probe ultrasonication parameters were the following; power of 100 W, frequency of 30 kHz, 0.5 cycle, and 100% amplitude. The probe ultrasonication was performed for ≈10 min at room temperature using a water bath without active cooling.

LMMPs with MWCNT‐COOH (denoted as LMMPs‐CNT) were prepared by ultrasonication. 16.08 to 23.90 grams of eGa_75.5_In_24.5_, 2.5 grams of IPA, and 0.02 to 0.29 grams of MWCNT‐COOH. The components were added to a glass vial and ultrasonicated for 10 min. LMMPs with both MWCNT‐COOH and HPC (denoted as LMMPs‐HPC) were prepared from two mixtures. First, 3.90 grams of deionized water (DIW), 1 gram of HPC, and 0.1 gram of MWCNT‐COOH were added to a glass vial and mixed. Then, 1 gram of the DIW/20 wt.% HPC/2 wt.% MWCNT‐COOH mixture was added to 16.08 to 23.90 grams of eGa_75.5_In_24.5_ and 2.5 grams of IPA. Finally, the mixture was ultrasonicated for 10 min at room temperature using a water bath without active cooling.

### Synthesis of Electrically Conductive Liquid Metal Elastomers (ECLME)

EC7 was used as the elastomer matrix composition^[^ ^18^ ^]^ for the preparation of electrically conductive liquid metal elastomers (ECLMEs). Typically, 0.017 grams of B_2_O_3_ NPs were mixed with 0.250 grams of the polymer base of dimethylvinyl‐terminated dimethylsiloxane. Then, 1.683 grams of PDMS‐OH was added to the prepared LMMPs dispersion and further mixed. A mixture of PDMS‐OH/LMMPs was added to the mixture of B_2_O_3_ NPs/polymer base and further mixed with mortar and pestle. In the case that the surfactant was used, 0.116 grams of TritonTM X‐100 was added to the mixture of PDMS‐OH/LMMPs/B_2_O_3_ NPs/polymer base. Finally, 0.050 grams of the cross‐linker of dimethylvinyl‐terminated dimethylsiloxane was added, and the mixture was further mixed to form the ECLME ink.

### Fabrication of Functional ECLME Structures

The ECLME inks were printed with blade coating onto substrates through shadow masks (100 µm PET film) designed with AutoCAD and cut according to the design with the laser (LPKF ProtoLaser U3). The printed ECLME structures were then cured in an oven at 70 °C overnight. Finally, ECLME film surface was photothermally activated with the laser (LPKF ProtoLaser U3) using the hatching tool. After adjusting the laser focus, the resistance of the ECLME was measured before and after photothermal activation. The process was repeated until optimal photothermal activation parameters were determined based on resistance measurements and surface optical micrograph imaging (e.g., power of 0.8 W, frequency of 50 kHz, and hatching mark speed of 400 mm s^−1^).

### Material Characterization

Particle sizes and distributions were analyzed with dynamic light scattering measurements using Zetasizer Nano ZS (Malvern Panalytical Ltd., UK). Fourier‐transform infrared spectroscopies were performed with ALPHA II Bruker ATR‐FTIR from wavenumbers 4000 to 400 cm^−1^. Optical micrographs were taken with an Olympus BX51 optical microscope equipped with a ColorView digital microscope camera. Field Emission Scanning Electron Microscopy (FESEM) was done with Zeiss ULTRA Plus (Carl Zeiss SMT AG, Germany) equipped with an energy dispersive X‐ray spectroscopy (EDS) system under an acceleration voltage of 15 kV. X‐ray photoelectron spectroscopy (XPS) analysis was done with ESCALAB 250Xi (Thermo Fisher Scientific) by using Avantage software. Thermogravimetric analysis (TGA) and differential scanning calorimetry (DSC) were conducted with NETZSCH STA 449F3 Jupiter for elastomers over a temperature range of 30–800 °C in a nitrogen atmosphere with a heating rate of 10 °C min^−1^. Atomic Force Microscope (AFM) using tapping mode was done with Bruker Multimode 8 equipped with Nanoscope V controller by using TAP150A Si probes (a nominal cantilever with a spring constant of 5 N m^−1^, and a resonant frequency of 150 kHz).

Tensile properties were measured with Stable Microsystem Texture Analyzer TA750 equipped with 50N‐load cell and pneumatic clamps from pristine and self‐healed samples. For self‐healing analysis, the pristine specimens were bisected with a razor perpendicularly to the direction of uniaxial stretching. The cut‐surfaces were manually aligned and left to self‐heal for 1–72 h in ambient conditions before the measurements. Electrical current was measured at 1 V with a Keithley 2604B source meter by using a four‐wire, two‐probe setup. The resistance as a function of time was plotted from the electrical current using the LabView program. The electrical conductivity with and without tensile strain was calculated as a reciprocal of the resistivity (*σ_dc_ = ρ^−1^ = R^−1^A^−1^l*, where *R* was the resistance, *A* was the film cross‐sectional area, and *l* is the distance between the probes). The thickness for ECLME printed layers was measured with the cross‐section FESEM images for different compositions. The printed ECLME structures were stretched with TA750 at strain rates of ≈0.1 to 6% s^−1^. The electrical measurement was synchronized with the stretching of the sample. For the calculation of conductivity under strain, the relative change of resistance was taken into account, and measurement contact resistance was neglected. The change in length was taken only into consideration due to non‐homogenous strain distribution (i.e., cross‐section area fixed to that of zero strain). Resistances as a function of temperature and humidity, and for 85 RH%/85 °C test were measured with Keithley 2604B source meter with the LabView program in environmental chamber (LabEvent L, Weiss Technik, France).

### Fabrication of ECLME Demonstrators

ECLME‐based LED‐display was fabricated first by vertically blade coating of ECLME_65_‐CNT_0.75_ (ECLME) interconnections using a shadow mask onto an EC7‐CNT_0.1_ self‐healing substrate and cured overnight at 70 °C for a 24 h. The printed ECLME interconnections were vertically photothermally activated with a laser. Small connectors were made by printing ECLME onto EC7 substrate. The small ECLME/EC7 connectors were used to connect the two parts of the ECLME interconnections (Figure S31, Supporting Information). To form the display with a 3 × 5 matrix, 15 blue clear LEDs (50120BS75000, Würth Elektronik) were used with a common anode. The LEDs were placed with tweezers in their predesigned locations and pushed gently to their places. To improve the bonding of the components to the ECLME interconnections in the underwater display, small EC7 were added to the edges of the LEDs (Figure S31, Supporting Information). The copper wires were attached to the ECLME interconnections to connect the display to the electronics. The parts of the copper wires that attached to the ECLME interconnections were covered with additional EC7 pieces to improve the bonding before encapsulation with Dragon Skin. Finally, the display was encapsulated with a thin layer of EC7.

The interconnectors and electrodes of the ECLME‐based battery integrated device demonstrator were fabricated in a similar way to the LED‐display (as shown in Figures S36 and S37, Supporting Information). The CR2032 battery was placed onto the lower ECLME electrode then the upper electrode was used to connect the battery to the ECLME interconnection. Three LEDs were placed with tweezers in their predesigned locations and pushed gently to their places. Finally, the device was encapsulated with a thin layer of EC7.

### Statistical Analysis

Continuous variable were expressed as mean ± STD (n ≥ 3; Figure 2a,b; Figures S14, S15, S23, S24, S36, and S38, and Tables S1 and S2, Supporting Information). The data was not pre‐processed, and statistical tests or any software was not used for the analysis of significance.

## Conflict of Interest

Authors A.A., M.N., J.H., J.J., H.J. and J.T. have issued patent applications (EPO 24197471.6, 24197480.7 and 24210058.4) related to processes, materials, and devices described in this article.

## Supporting information



Supporting Information

Supplemental Movie 1

Supplemental Movie 2

Supplemental Movie 3

Supplemental Movie 4

Supplemental Movie 5

Supplemental Movie 6

Supplemental Movie 7

Supplemental Movie 8

Supplemental Movie 9

## Data Availability

The data that support the findings of this study are available in the supplementary material of this article.
