# Supplementary material for: Stable, Highly Conductive, and Strain‐Insensitive Supramolecular Elastomer Composite for Printable Self‐Healing Soft Electronics
Source: Adv Sci (Weinh). 2025 Jun 25;12(33):e05011. doi: 10.1002/advs.202505011 (PMC12412568; doi:10.1002/advs.202505011)
Supplement: Supplementary file 1 — Supporting Information [file ADVS-12-e05011-s010.pdf]

ADVANCED SCIENCE
Open Access

Supporting Information

for *Adv. Sci.*, DOI 10.1002/advs.202505011

Stable, Highly Conductive, and Strain-Insensitive Supramolecular Elastomer Composite for Printable Self-Healing Soft Electronics

Ahmed Albeltagi, Tiia Tyystälä, Mikko Nelo, Tuomo Siponkoski, Aldeliane M. da Silva, Mari Rocham, Jari Hannu, Heli Jantunen, Jari Juuti and Jarkko Tolvanen*

Supporting Information

Stable, highly conductive and strain-insensitive supramolecular elastomer composite for printable self-healing soft electronics

Ahmed Albeltagi, Tiia Tyystälä, Mikko Nelo, Tuomo Siponkoski, Aldeliane Maria Da Silva, Mari Rocham, Jari Hannu, Heli Jantunen, Jari Juuti, and Jarkko Tolvanen*

Table of Contents

Figure S1. Dynamic Light Scattering (DLS) measurement. 4
Figure S2. Stability of LMMP dispersions after ultrasonication. 5
Figure S3. Field Emission Scanning Electron Microscopy (FESEM) images.......................... 6
Figure S4. FESEM images of films cross-section. 7
Figure S5. Optical micrographs of pristine ECLMEs.. 8
Figure S6. Optical micrographs of ECLMEs after cut.. 9
Figure S7. FESEM images of printed ECLMEs on substrates. 10
Figure S8. FESEM and Energy Dispersive Spectroscopy (EDS) images. 11
Figure S9. Photographs of printed ECLMEs after cutting.. 12
Figure S10. Micrographs of printed ECLMEs before and after cut.. 13
Figure S11. Photographs of self-healed ECLME patterns.. 14
Figure S12. Photographs of ECLME patterns under areal strain. 15
Figure S13. Schematic illustration of photothermal activation. 16
Figure S14. Resistance ratio (*R/R_0*) plotted as a function of time.. 17
Figure S15. *R/R_0* plotted as a function of time for different substrates. 18
Figure S16. Resistance stability after aging…………………………………………………..19
Figure S17. Thermogravimetric Analysis (TGA) and Differential Scanning Calorimetry (DSC).. 20
Figure S18. Atomic force microscopy (AFM) images. 21
Figure S19. Topography and phase AFM images. 22
Figure S20. Fourier-transform infrared spectroscopy (FTIR).. 23
Figure S21. FTIR for ECLMEs. 24
Figure S22. FTIR for ECLME-HPCs.. 25
Figure S23. Schematic illustration of the printing and lasering direction. 26
Figure S24. Electrical conductivities measured as a function of CNT loading.. 27
Figure S25. Electrical conductivity as a function of liquid metal loading.. 28
Figure S26. Resistance ratio as a function of strain on DS substrate. 29
Figure S27. Conductivity ratio as a function of strain on DS substrate. 30
Figure S28. Electro-mechanical properties as a function of strain on DS substrate with higher CNT loading in ECLME. 31
Figure S29. Electro-mechanical properties of ECLME on elastomer substrate with self-healing intermediate layer. 32
Figure S30. Photographs of ECLME and ECLME-HPC on DS substrate............................. 33
Figure S31. Electro-mechanical properties of ECLME-HPC on DS substrate. 34
Figure S32. Electro-mechanical properties of ECLME-HPC on EC7 substrate.. 35
Figure S33. Mechanical properties of conventional elastomers.. 36
Figure S34. Tensile properties of EC7-CNT with varied CNT loading. 37
Figure S35. Tensile properties of EC7 with varied B_2O_3 content.. 38
Figure S36. Tensile properties of EC7-CNT. 39
Figure S37. Mechanical properties for EC7-CNT. 40
Figure S38. Mechanical self-healing for EC7 and EC7-CNT substrates. 41
Figure S39. Self-healing efficiency for EC7 and EC7-CNTs. 42
Figure S40. Schematic illustration of the display fabrication.. 43
Figure S41. Schematic illustration on interconnections and bonding of components. 44
Figure S42. Schematic illustration of the electronics for controlling the display. 45
Figure S43. Electro-mechanical stability after aging………………………………………..46
Figure S44. Underwater stability of ECLME on conventional elastomers. 47
Figure S45. Photographs of self-healing display underwater. 48
Figure S46. Photographs of the self-healing display underwater.. 49

Figure S47. Schematic illustration of ECLME-based battery-integrated LED demonstrator structure. 50
Figure S48. Schematic illustration of the fabrication process of battery-integrated device.. .. 51
Figure S49. Photographs of the battery-integrated LED demonstrator. 52
Figure S50. Photographs of LED replacement. 53
Table S1. Comparison table for the state-of-the-art liquid metal-based stretchable conductors.. 54
Table S2. Comparison table for the state-of-the-art autonomous self-healing conductors.. 55
Table S3. Tensile and self-healing properties for EC7 and EC7-CNTs. 56
Table S4. Comparison table for the state-of-the-art of self-healing elastomers. 57
Table S5. Tensile properties for ECLME. 59
Table S6. Comparison table for the stretchable displays.. 60
Movie S1. 62
Movie S2. 62
Movie S3. 62
Movie S4. 62
Movie S5. 62
Movie S6. 62
Movie S7. 62
Movie S8. 62
Movie S9. 62
References 63

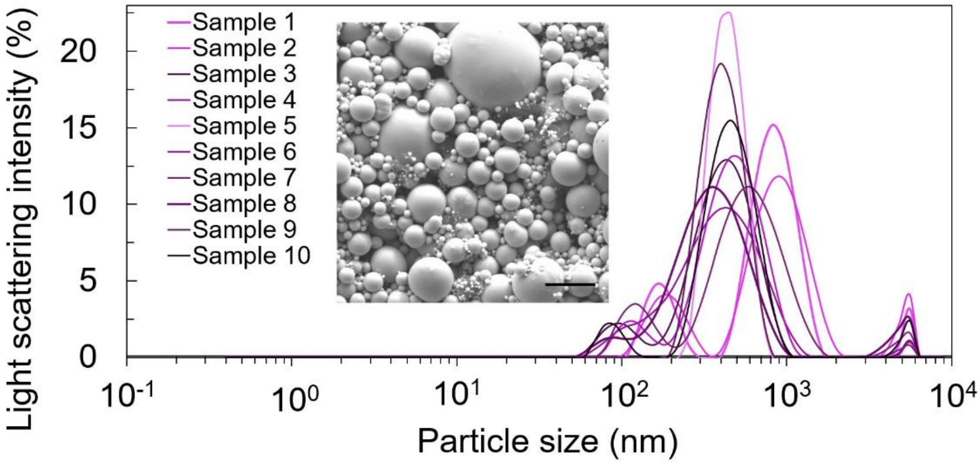

Figure S1. Dynamic Light Scattering (DLS) measurement. DLS particle size distribution for liquid metal microparticle (LMMP) suspensions prepared with probe ultrasonication (10 minutes). The suspensions were diluted with a solvent before the measurement. FESEM inset shows LMMP suspension printed on glass substrate. Scale bar 10 µm.

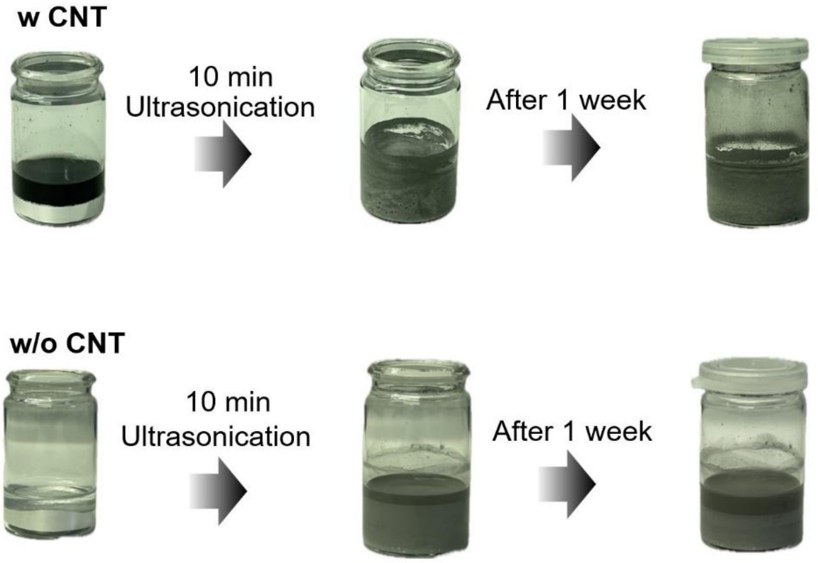

Figure S2. Stability of LMMP dispersions after ultrasonication. The dispersion consists of 23.92 grams eutectic gallium (75.5 wt.%) and indium (24.5 wt.%) (i.e., $eGa_{75.5}In_{24.5}$) mixed with 0.091 grams of carboxylic acid functionalized multiwalled carbon nanotubes, and 2.5 grams of isopropanol alcohol (IPA). In the second LMMP dispersion, the CNTs are left out.

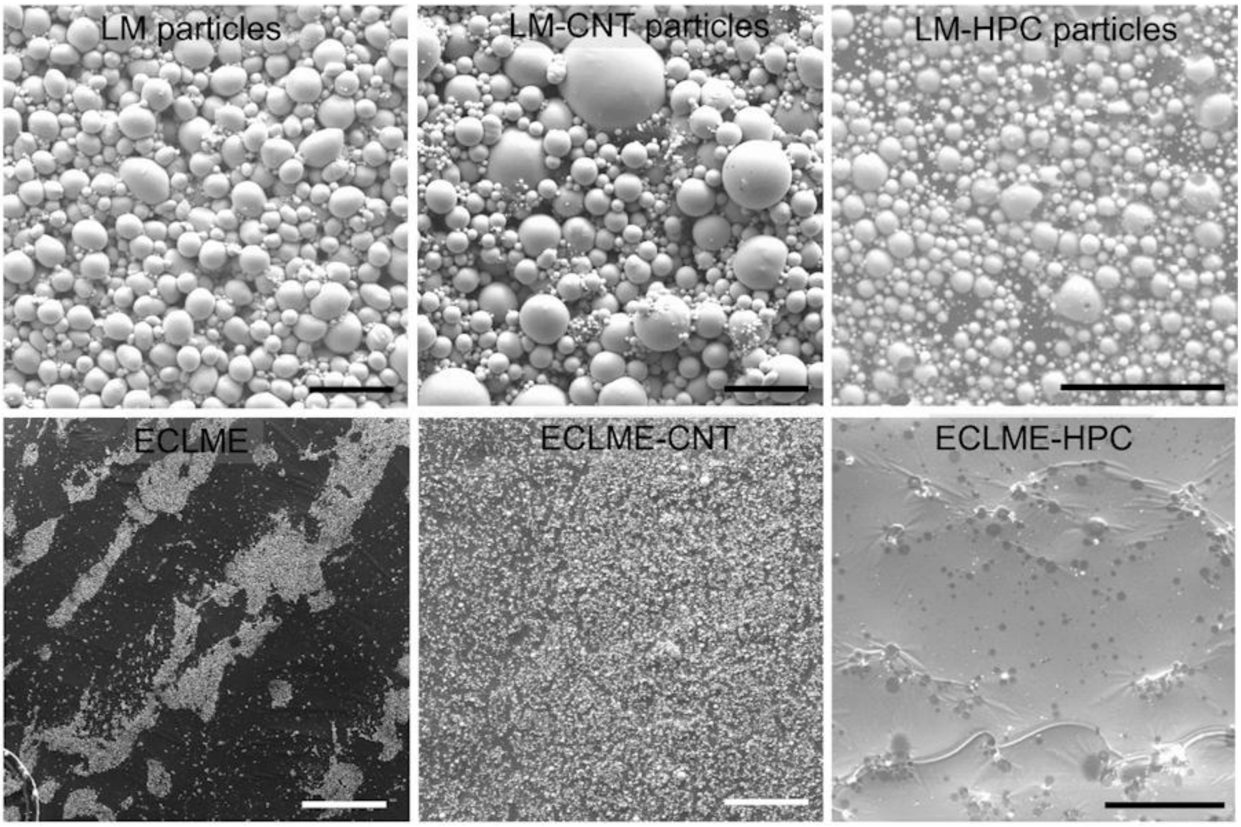

Figure S3. Field Emission Scanning Electron Microscopy (FESEM) images. FESEM images of printed LMMPs with and without CNTs, and with hydroxypropyl cellulose (HPC) to glass substrate. Scale bars 10 µm (top row). FESEM images of electrically conductive liquid metal elastomer (ECLME) films with and without CNTs, and with HPC. Scale bars 100 µm (bottom row).

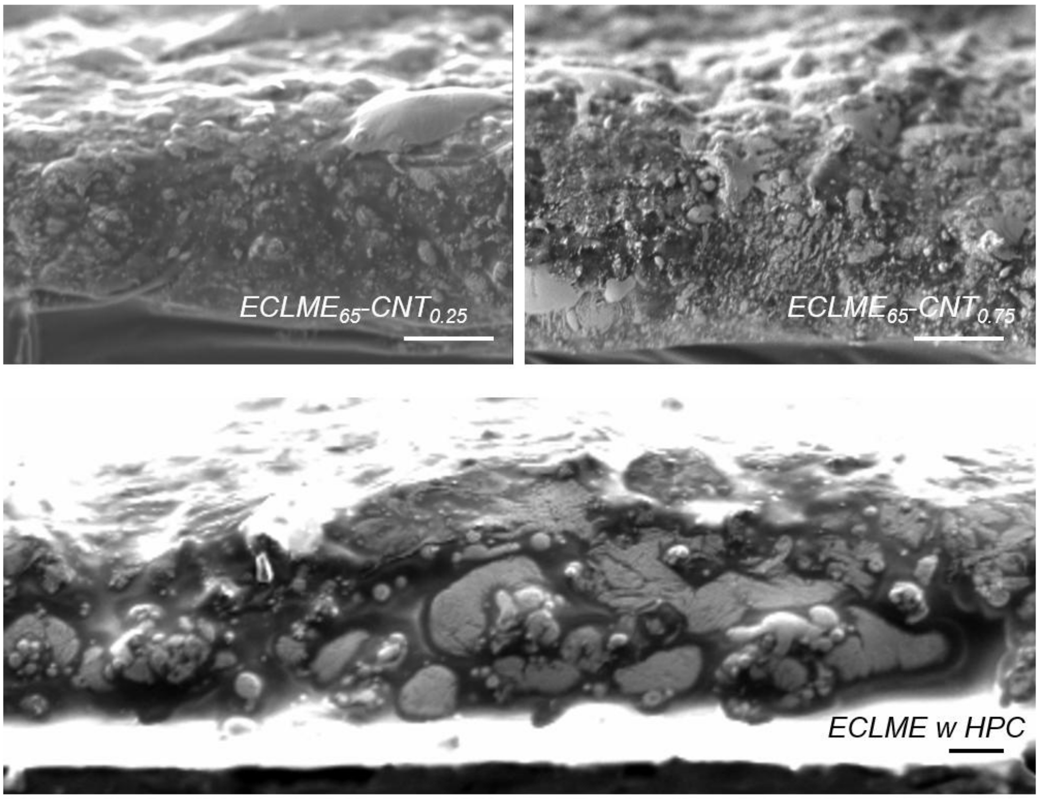

Figure S4. FESEM images of films cross-section. Cross-sectional FESEM images of ECLME films with 65 vol.% of $eGa_{75.5}In_{24.5}$ and 0.25 or 0.75 vol.% of CNTs, and ECLME with HPC. Scale bars 12.5 µm.

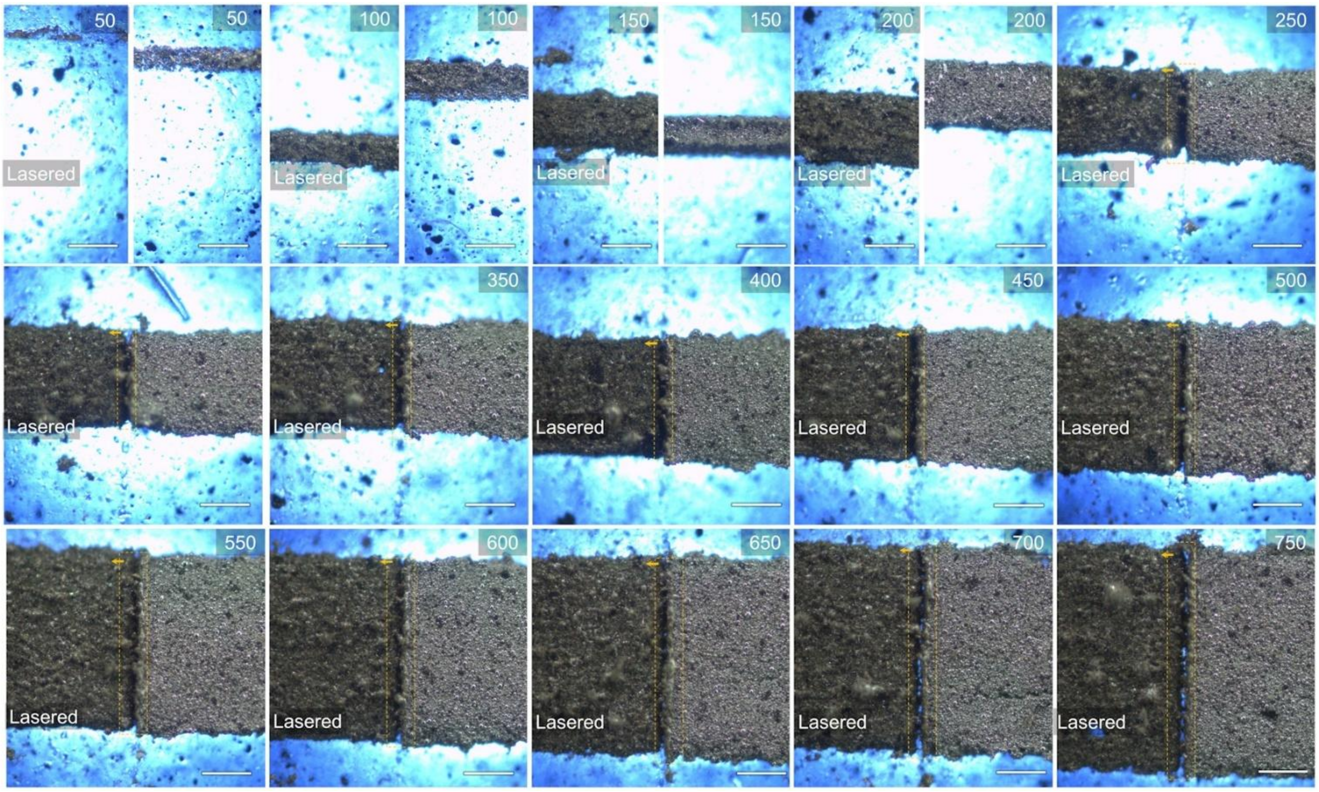

Figure S5. Optical micrographs of pristine ECLMEs. Optical micrographs of printed ECLMEs at different line widths (50 - 750 µm) with and without photothermal activation by the laser. The orange dashed area shows the starting point of the laser beam on the film surface. Scale bars 150 µm.

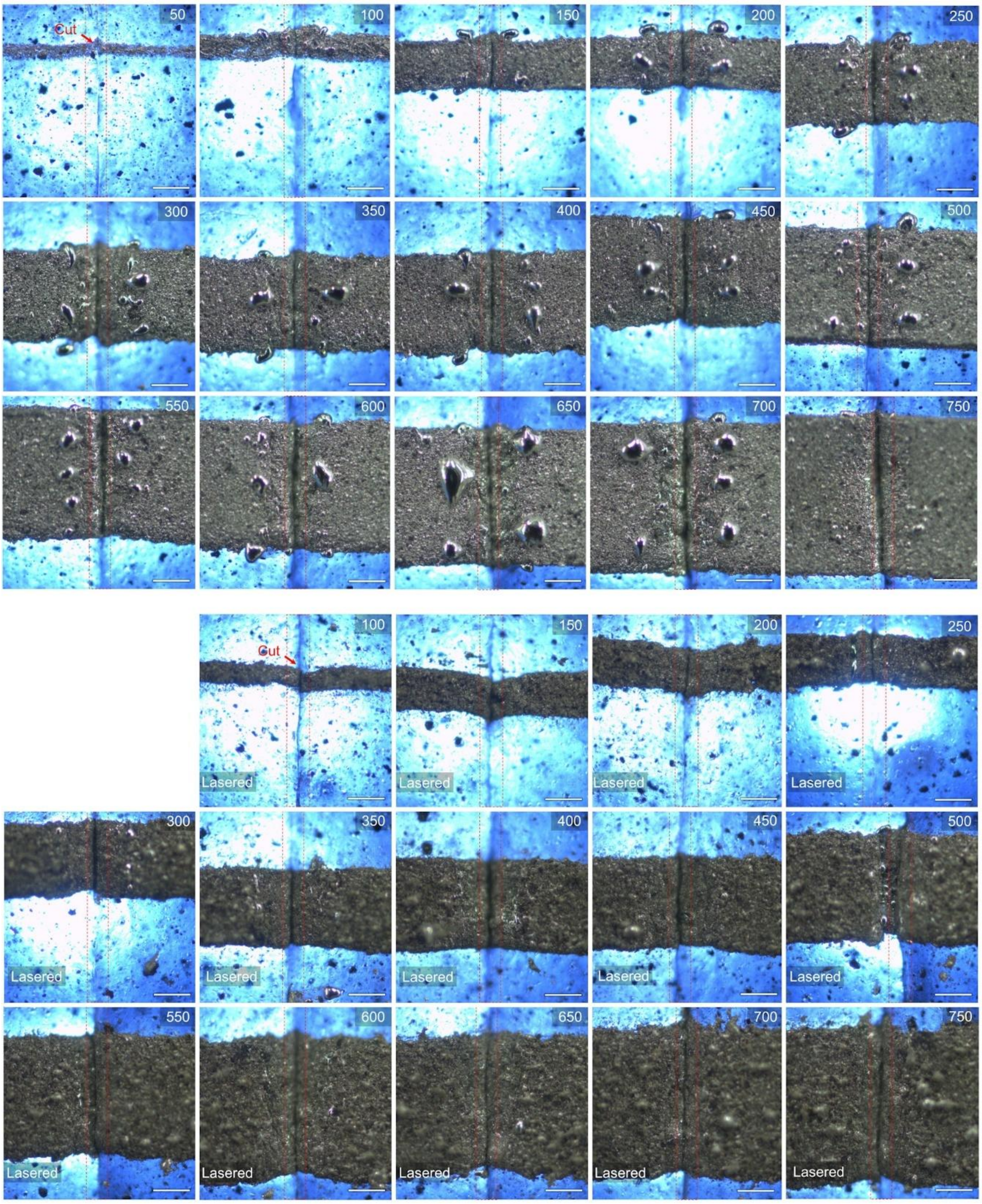

Figure S6. Optical micrographs of ECLMEs after cut. Optical micrographs of printed and photothermally activated ECLMEs at different line widths (50 - 750 µm) after completely bisected with razor. Scale bars 150 µm.

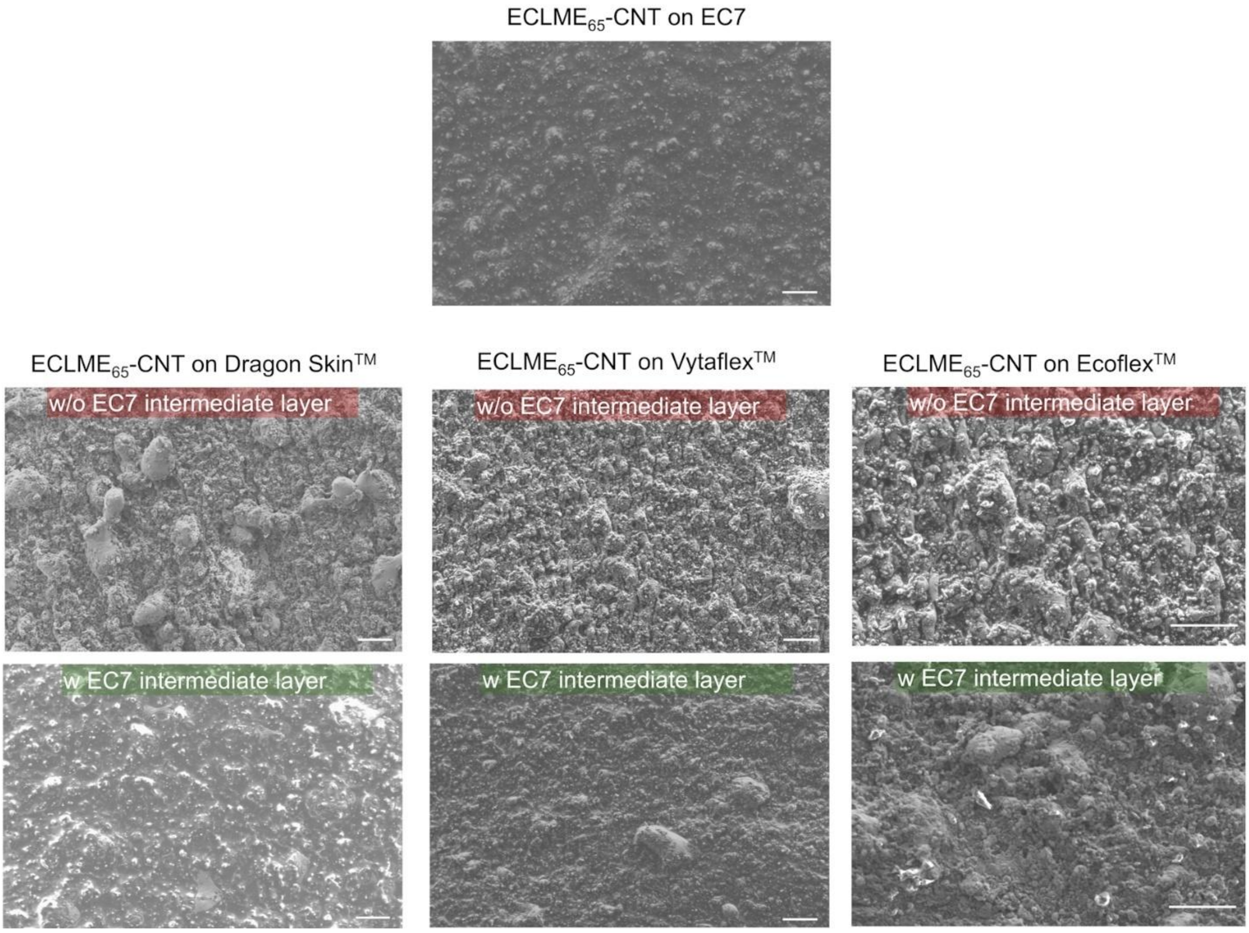

Figure S7. FESEM images of printed ECLMEs on substrates. ECLMEs with 65 vol.% $eGa_{75.5}In_{24.5}$ and 0.75 vol.% CNT on self-healing substrate (denoted as EC7), Dragon Skin®, VytaFlex®, and EcoFlex® elastomers with and without EC7 intermediate layer. In this case, the intermediate layer means the second substrate layer positioned between the first substrate and printed ECLME layer. Scale bars 100 µm.

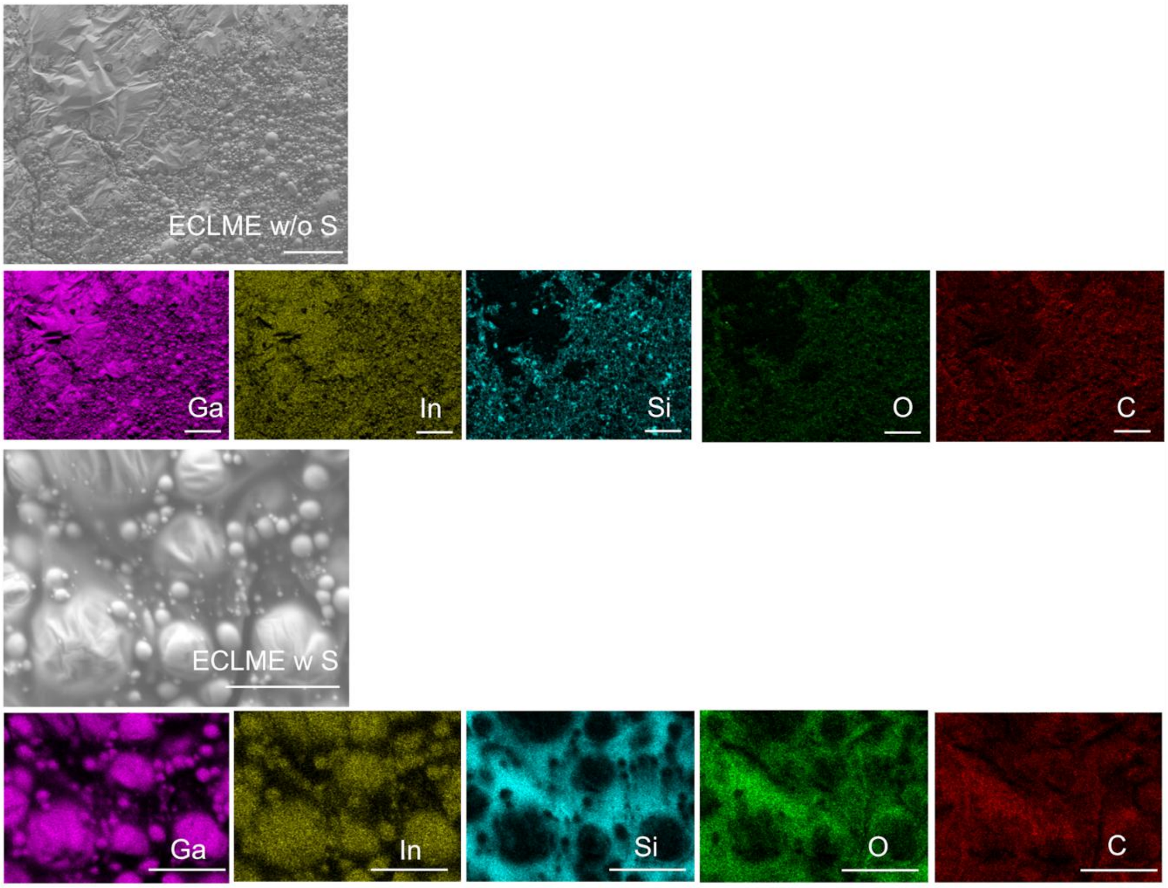

Figure S8. FESEM and Energy Dispersive Spectroscopy (EDS) images. FESEM and EDS images of ECLME films prepared on EC7 substrate without and with surfactant (Triton® X-100). Scale bars 100 µm (ECLME w/o S) and 10 µm (ECLME w S), respectively.

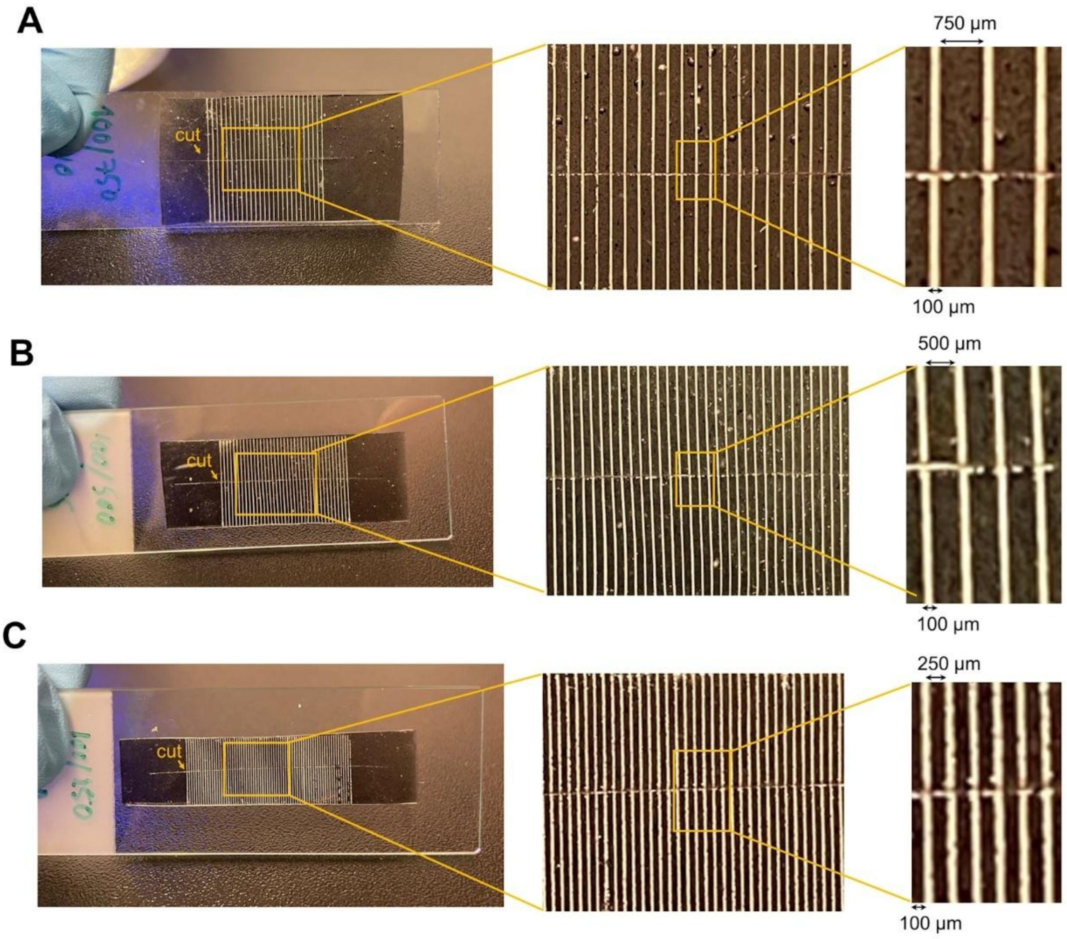

Figure S9. Photographs of printed ECLMEs after cutting. Samples were completely bisected (horizontally along the length of the lines) and then left for self-healing without cut-surface alignment. Line widths of 100 µm with gaps of (A) 750 µm, (B), 500 µm, and (C) 250 µm.

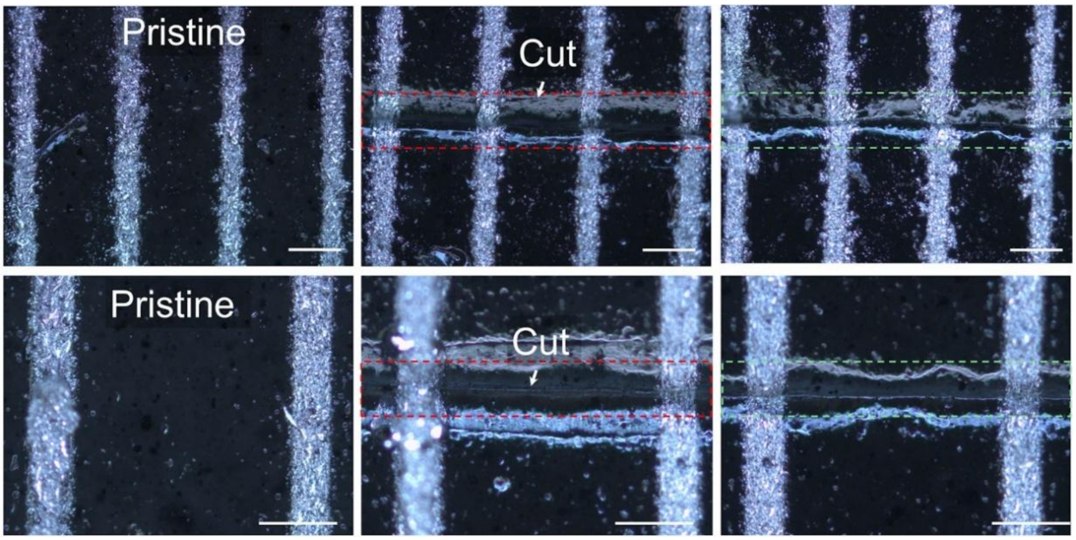

Figure S10. Micrographs of printed ECLMEs before and after cut. The samples were completely bisected and left for self-healing. Scale bars 200 µm.

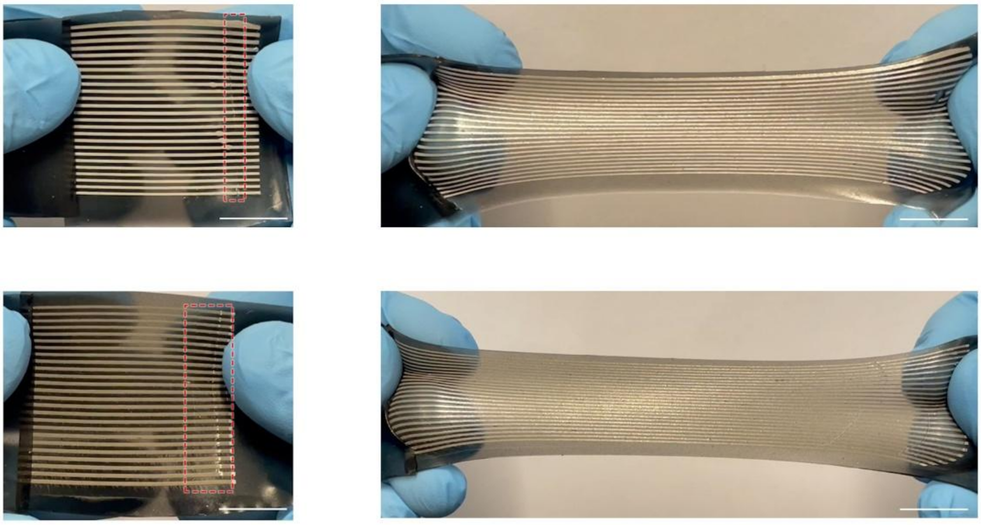

Figure S11. Photographs of self-healed ECLME patterns. Photographs of self-healed samples on EC7-CNT substrates that were completely bisected. The cut surfaces were aligned and left to self-heal in room temperature before stretching the samples. Scale bars 20 mm.

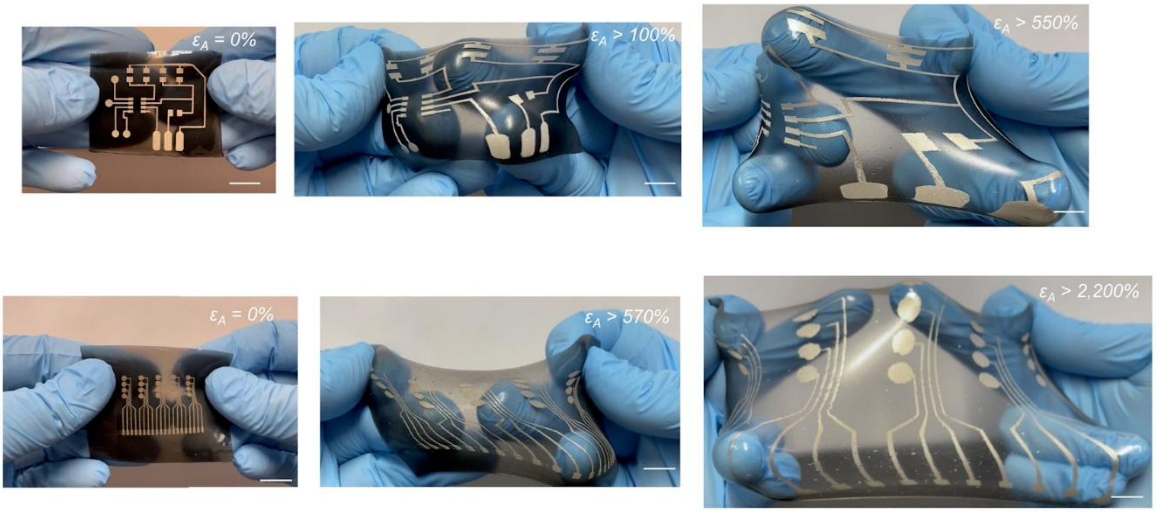

Figure S12. Photographs of ECLME patterns under areal strain. Self-healing printed circuit board and electrode under large areal strains (ε_A). The samples are made by printing ECLME-based ink on EC7-CNT substrate. Scale bars 10 mm.

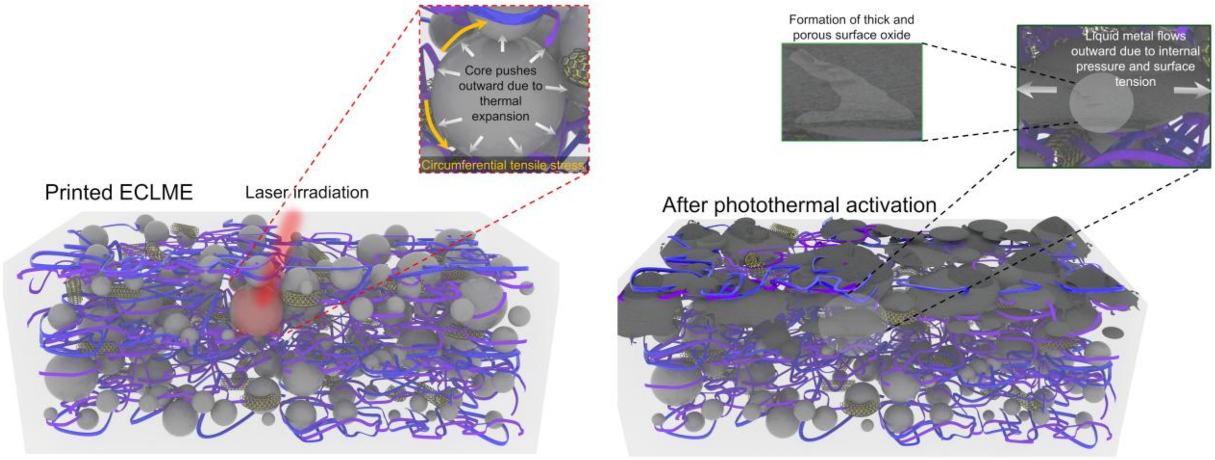

Figure S13. Schematic illustration of photothermal activation. Laser irradiation causes localized heating resulting in a rapid thermal expansion of the liquid metal core. As the core pushes outward, circumferential tensile strain develops in the shell. The liquid metal microparticles rupture once the stress exceeds the fracture strength of the oxide skin. As a result, the liquid metal flows outward due to internal pressure and surface tension. With the exposure to atmospheric oxygen, this results in a formation of the secondary oxide skin that is thicker and more porous than the original one.

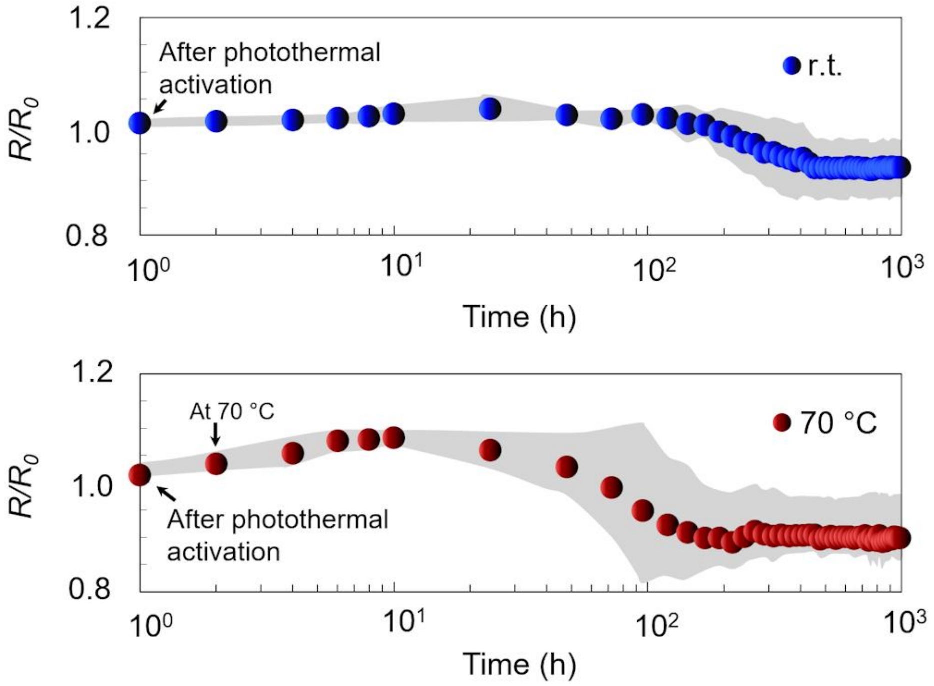

Figure S14. Resistance ratio (*R/R_0*) plotted as a function of time. *R/R_0* plotted as a function of time for photothermally activated ECLME films (65 vol.% $eGa_{75.5}In_{24.5}$ and 0.75 vol.% CNT) in room temperature (denoted as r.t.) and at temperature of 70 °C. The individual data points correspond to average *R/R_0* with shaded standard deviations ($n \geq 3$).

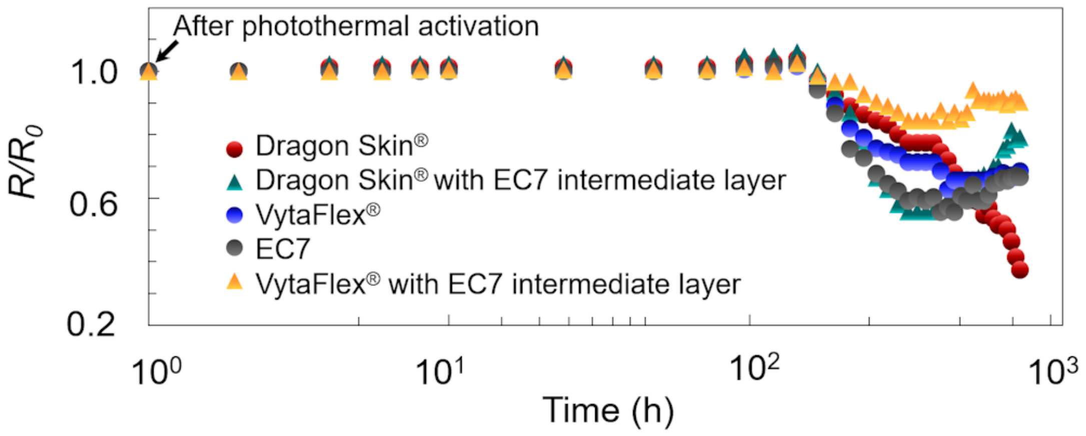

Figure S15. *R/R₀* plotted as a function of time for different substrates. Photothermally activated ECLME films (65 vol.% $eGa_{75.5}In_{24.5}$ and 0.75 vol.% CNT) in room temperature (denoted as r.t.) on different substrates. The individual data points correspond to an average R/R_0 (n ≥ 3).

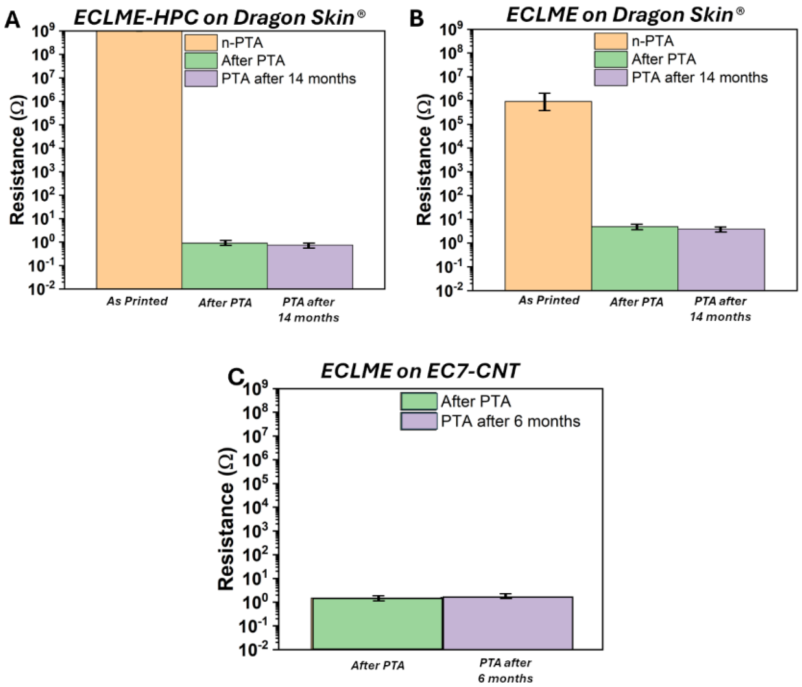

Fig. S16. Resistance stability after aging. Resistance of the ECLMEs measured before (n-PTA) and after photothermal activation (PTA), and after aging at room temperature (r.t.) for 6–14 months following PTA: a) ECLME-HPCs on Dragon Skin® elastomer, b) ECLMEs on Dragon Skin® elastomer, and c) ECLMEs on EC7-CNT substrate.

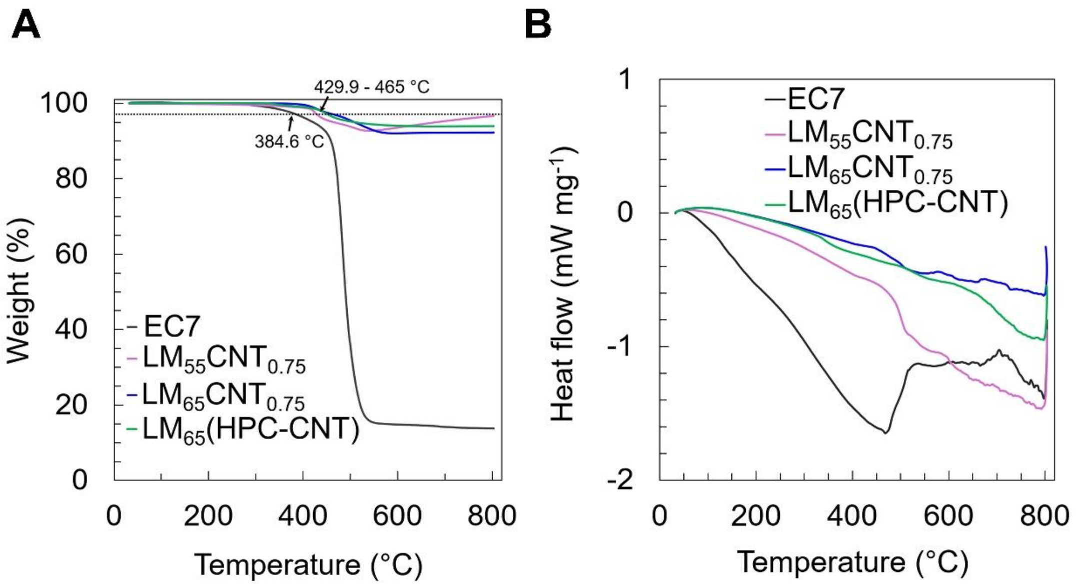

Figure S17. Thermogravimetric Analysis (TGA) and Differential Scanning Calorimetry (DSC). TGA and DSC measured for EC7 and ECLME films over temperature range of 30 – 800 °C in nitrogen atmosphere with heating rate of 10 °C min^{-1}. ECLME films were prepared with 55 and 65 vol.% $eGa_{75.5}In_{24.5}$ and 0.75 vol.% ~~of~~ CNT; and 65 vol.% $eGa_{75.5}In_{24.5}$ with CNT and HPC. The data was collected on the second heating process after eliminating the thermal history.

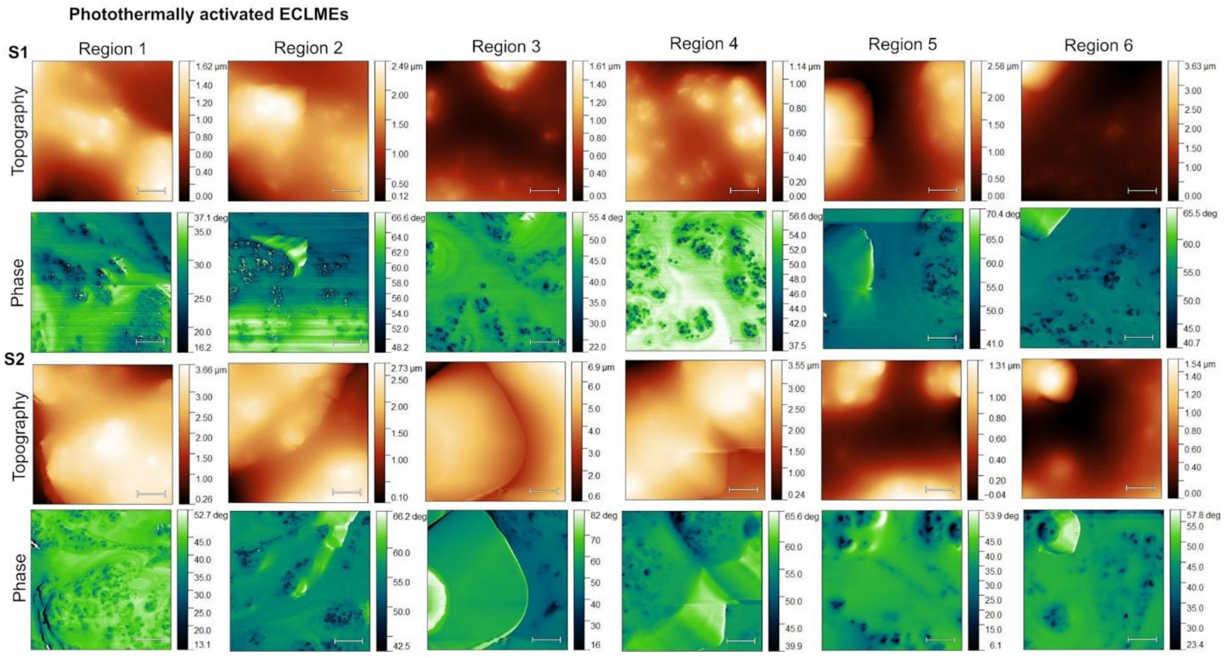

Figure S18. Atomic force microscopy (AFM) images. Topography and phase AFM images of photothermally activated ECLMEs in six distinct regions for two printed layers (denoted as S1 and S2). Scale bars 1 µm.

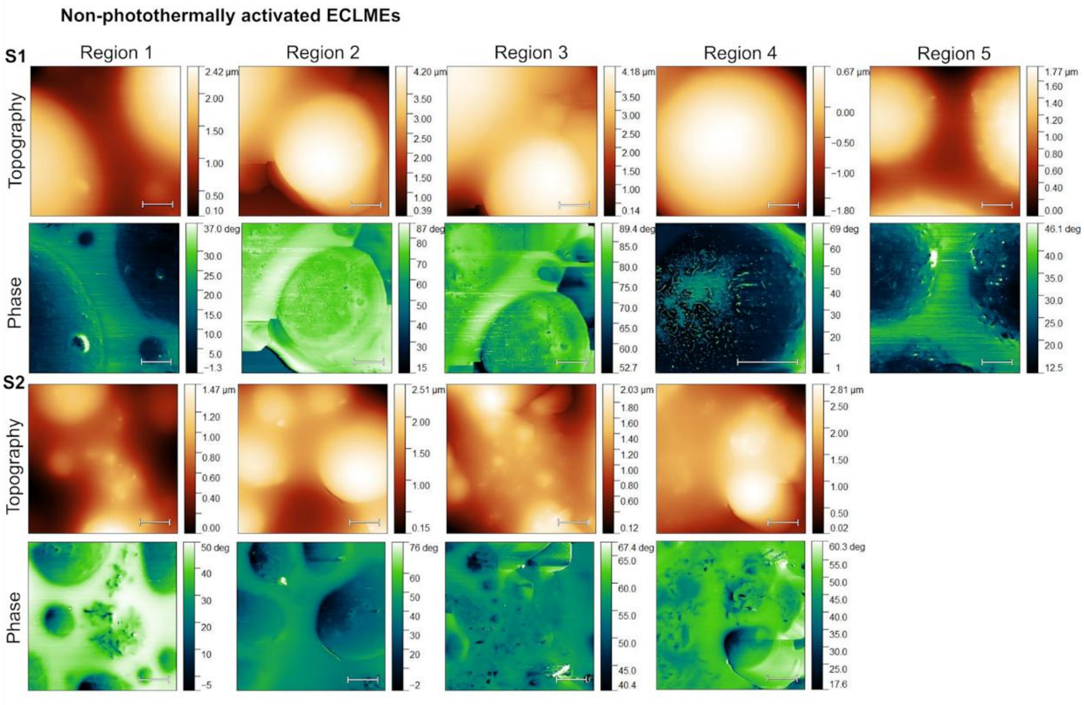

Figure S19. Topography and phase AFM images. Non-photothermally activated ECLMEs in four to five distinct regions for two printed layers (denoted as S1 and S2). Scale bars 1 µm.

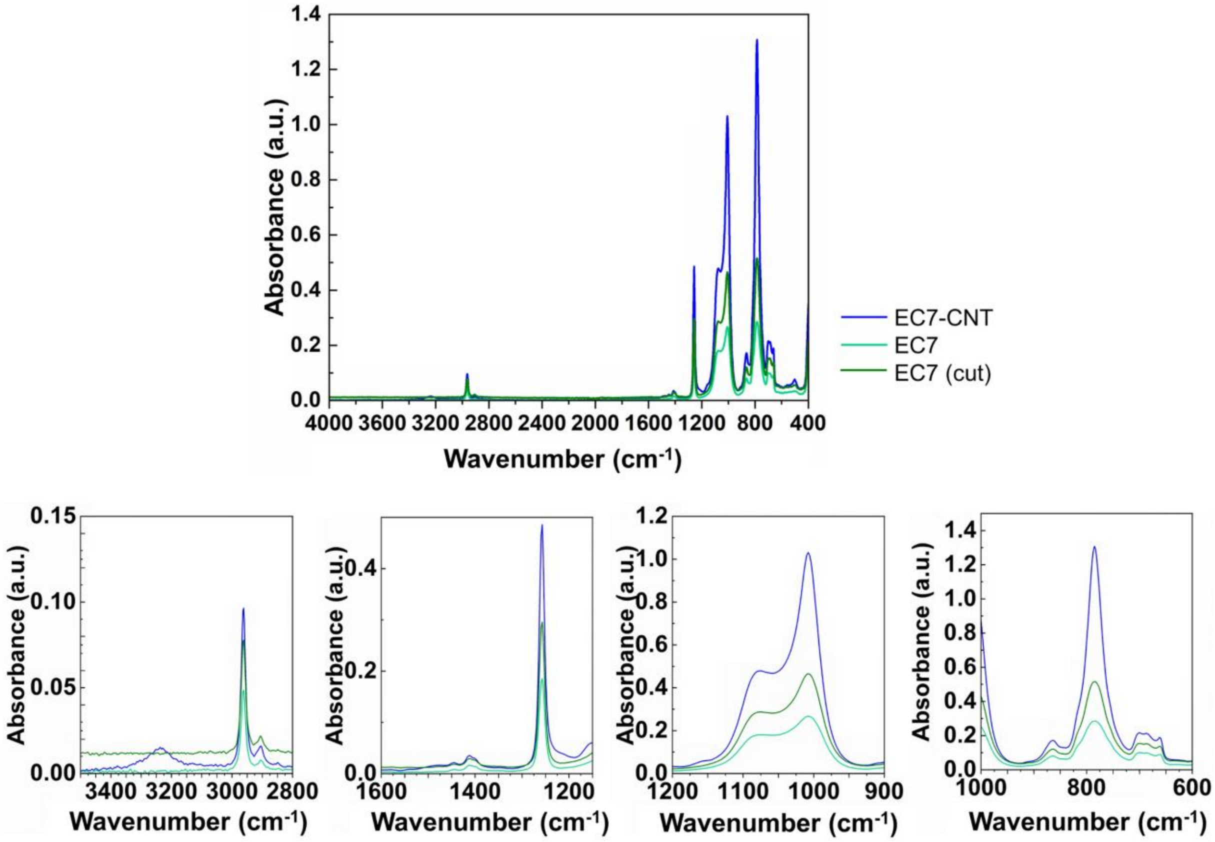

Figure S20. Fourier-transform infrared spectroscopy (FTIR). FTIR for self-healing elastomer substrates (EC7 and EC7-CNT) when applying force to the samples.

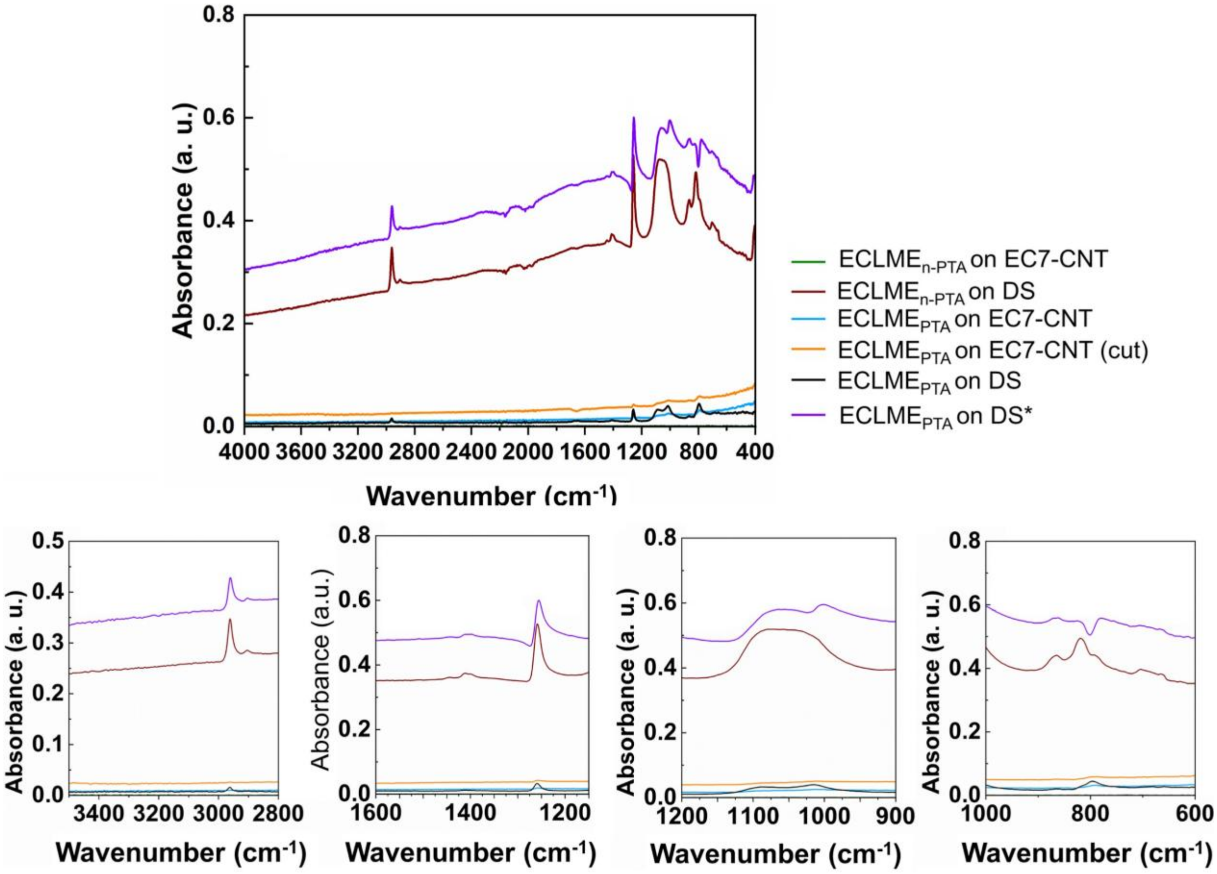

Figure S21. FTIR for ECLMEs. FTIR without application of force for non-photothermally activated ($ECLME_{n\text{-}PTA}$) and photothermally activated ($ECLME_{PTA}$) ECLMEs on EC7-CNT and Dragon Skin® elastomer substrates. DS* indicates a sample in which a force was applied to the sample during the measurement. Please note that the FTIR curve for $ECLME_{n\text{-}PTA}$ on EC7-CNT was nearly zero.

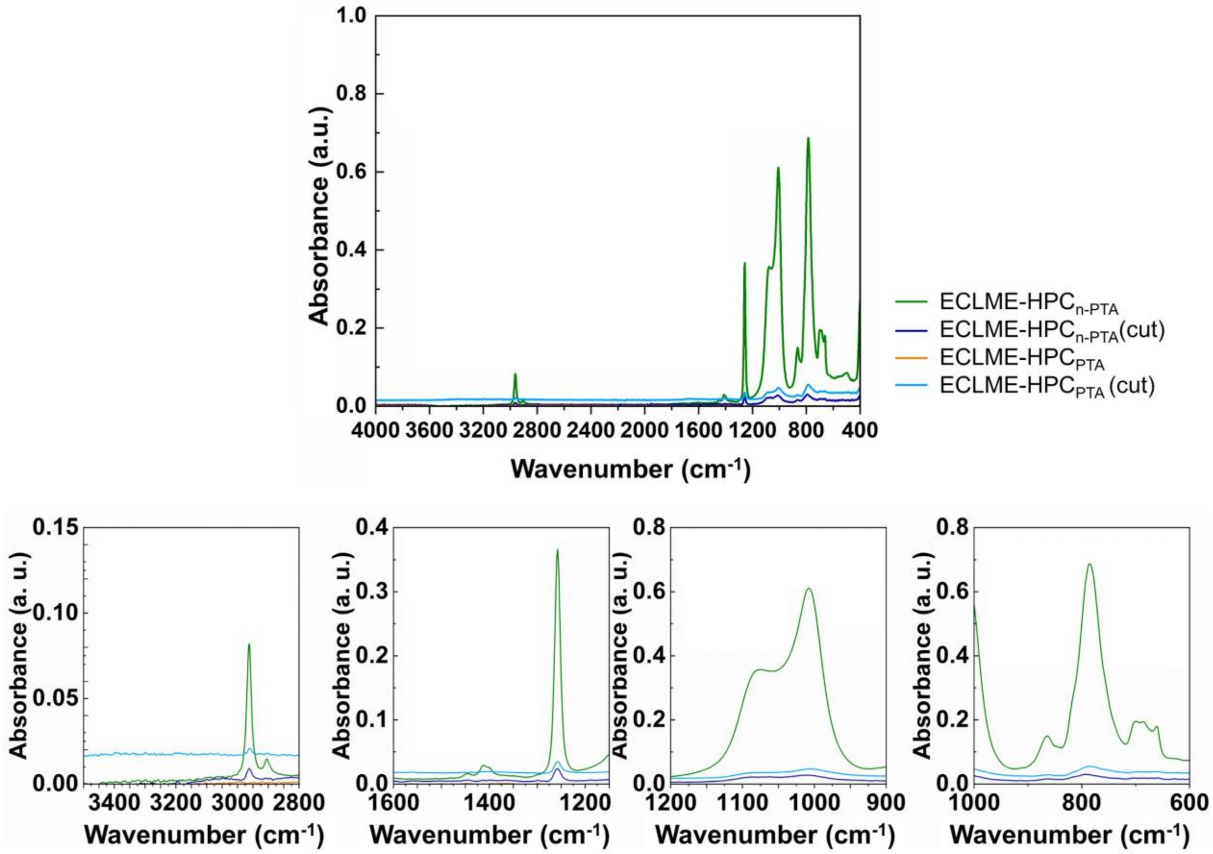

Figure S22. FTIR for ECLME-HPCs. FTIR without application of force for non-photothermally activated (ECLME-$HPC_{n\text{-}PTA}$) and photothermally activated (ECLME-HPC_{PTA}) ECLMEs with HPC on EC7-CNT substrate. Please note that the FTIR curve for ECLME-HPC_{PTA} was nearly zero.

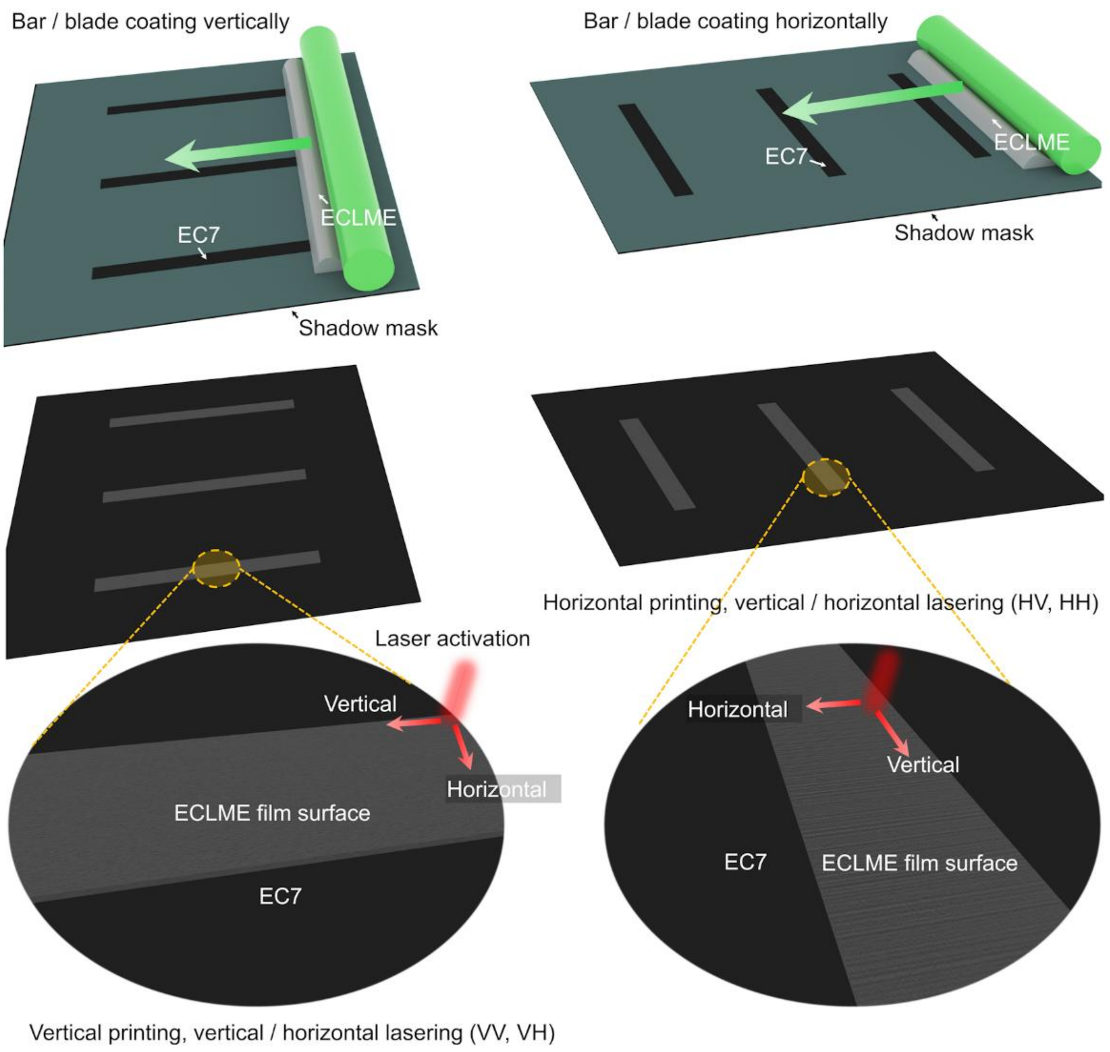

Figure S23. Schematic illustration of the printing and lasering direction. VV, VH, HV, and HH denotes vertically printed and lasered, vertically printed and horizontally lasered, horizontally printed and vertically lasered, and horizontally printed and lasered films, respectively. In this case, the vertical and horizontal mean along the length and width of the trace (as illustrated).

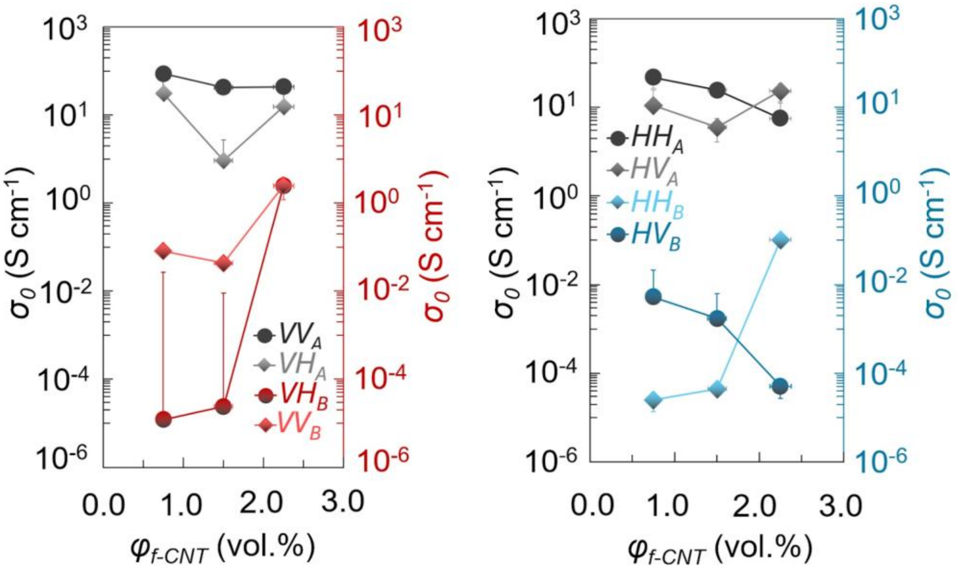

Figure S24. Electrical conductivities measured as a function of CNT loading. Electrical conductivities (σ_0) measured as a function of CNT loading ($\varphi_{f\text{-}CNT}$) before (denoted as subscript "B") and after photothermal activation (denoted as subscript "A") the film surface. All ECLME films with varied CNT loading had 65 vol.% $eGa_{75.5}In_{24.5}$. VV, VH, HH, HV denote the printing and lasering direction of ECLME films (V = vertical, and H = horizontal).

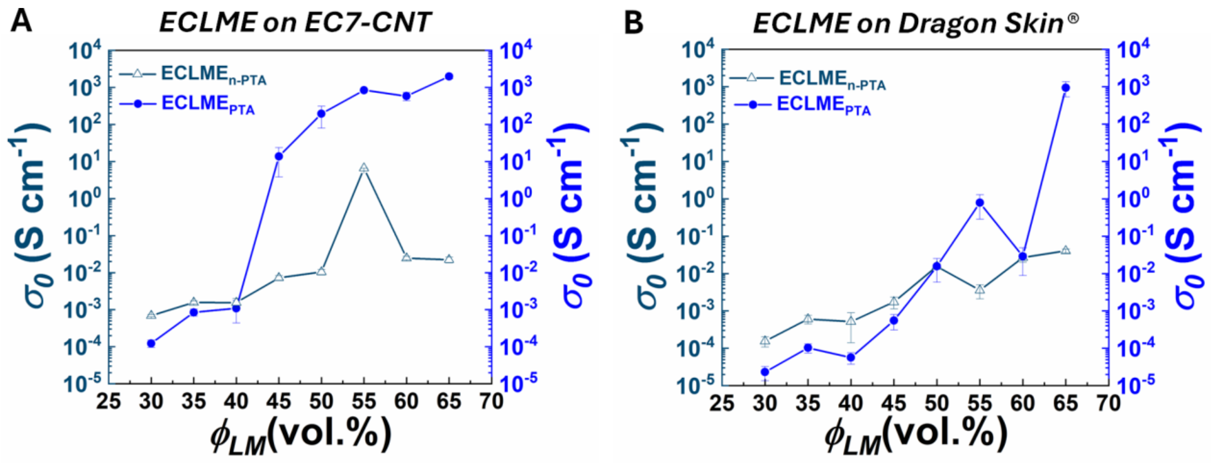

Figure S25. Electrical conductivity as a function of liquid metal loading. Electrical conductivities (σ_0) for ECLMEs before and after photothermal activation on (a) EC7-CNT and (b) Dragon Skin® substrates measured as a function of the $eGa_{75.5}In_{24.5}$ loading from 30 vol.% to 65 vol.% (CNT loading was fixed to 0.75 vol.%). Data plotted as mean ± STD ($n \geq 3$).

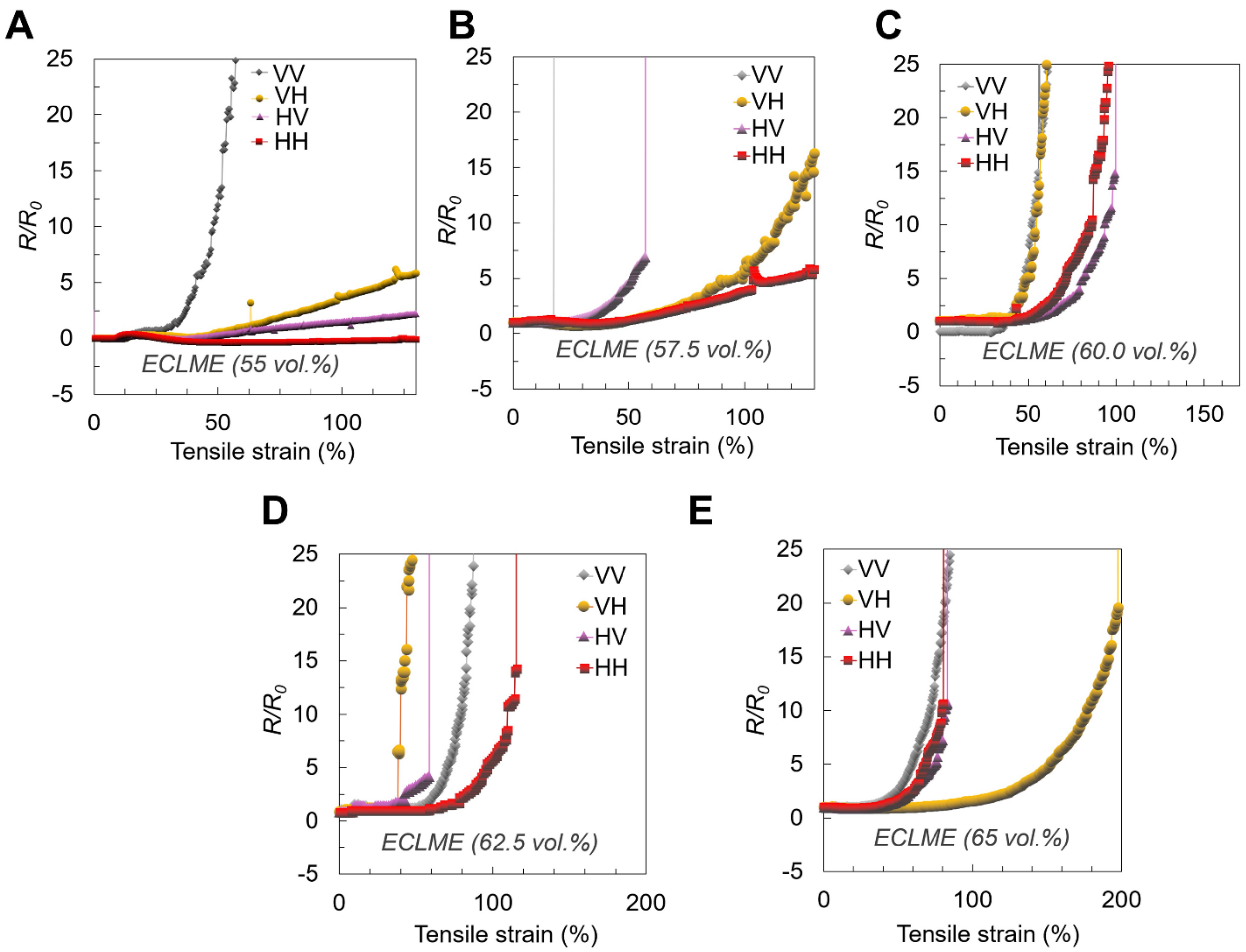

Figure S26. Resistance ratio as a function of strain on DS substrate. a-e) *R/R₀* plotted as a function of tensile strain for printed and photothermally activated ECLME layers on Dragon Skin® elastomer substrate. The filler loading of $eGa_{75.5}In_{24.5}$ varied from 55 vol.% to 65 vol.% (CNT loading was fixed to 0.75 vol.%). VV, VH, HV, and HH denote the combined printing and lasering direction (V = vertical, and H = horizontal). In this case, vertical and horizontal mean along the length and width of the trace, respectively.

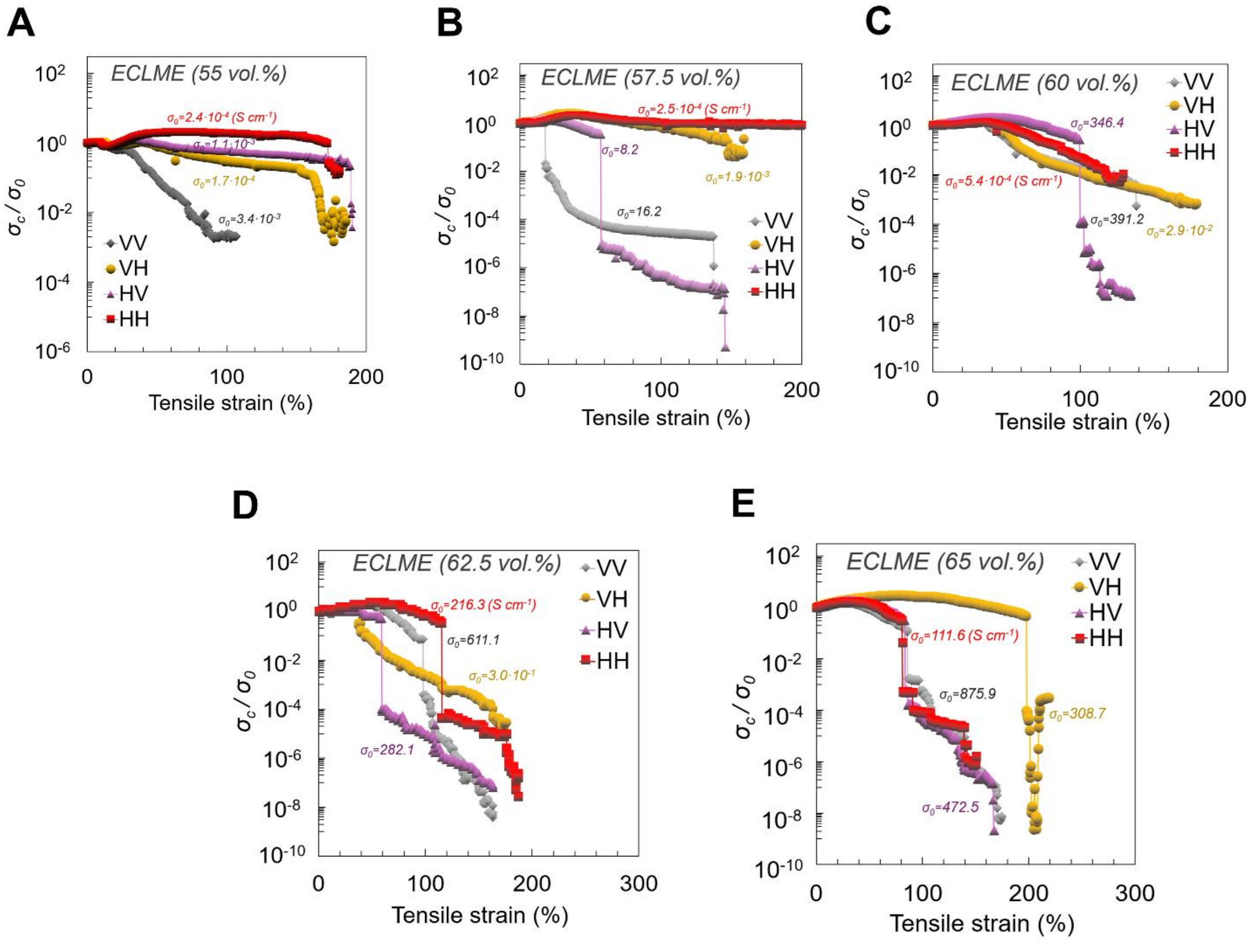

Figure S27. Conductivity ratio as a function of strain on DS substrate. a-e) σ_c/σ_0 plotted as a function of tensile strain for printed and photothermally activated ECLME layers on Dragon Skin® elastomer substrate. The filler loading of $eGa_{75.5}In_{24.5}$ varied from 55 vol.% to 65 vol.% (CNT loading was fixed to 0.75 vol.%). σ_c/σ_0 was expressed as the conductivity under specific tensile strain (σ_c) divided with the initial zero strain conductivity (σ_0). All σ_0 values were expressed as Siemens per centimeter ($S{\cdot}cm^{-1}$). The cross-sectional area decrease was not taken into consideration due to plausible non-homogenous stress distribution. VV, VH, HV, and HH denote the combined printing and lasering direction (V = vertical, and H = horizontal). In this case, vertical and horizontal mean along the length and width of the trace, respectively

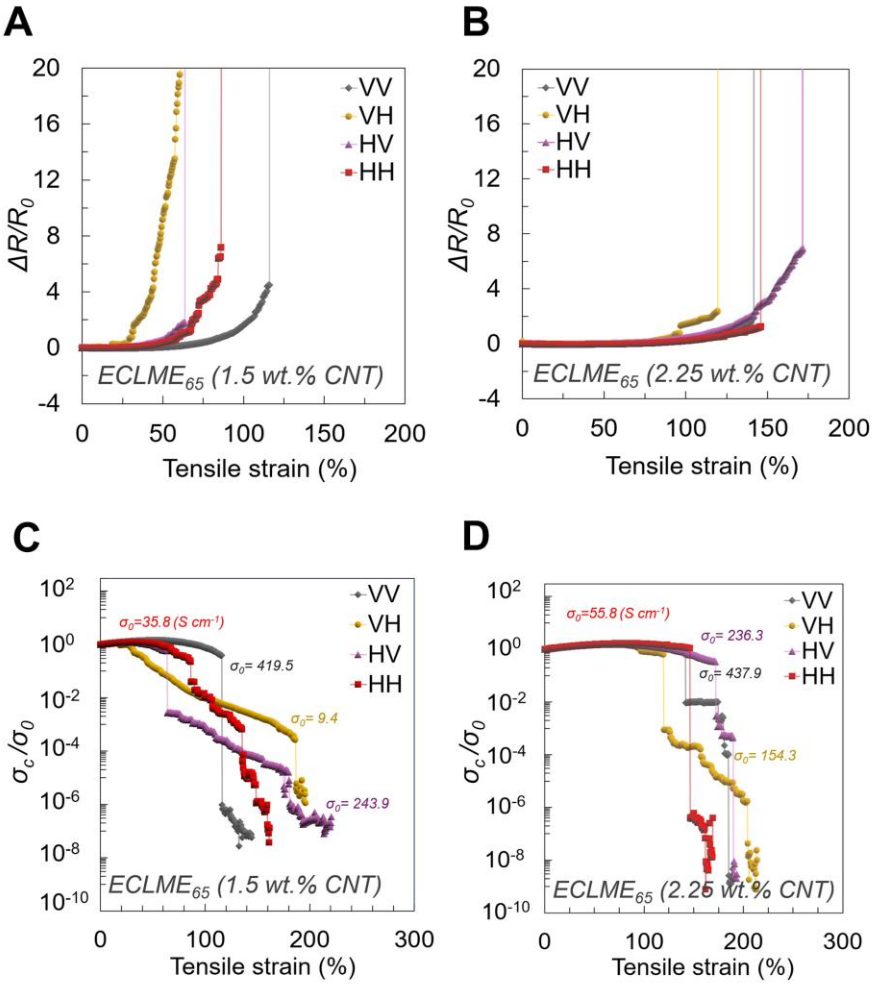

Figure S28. Electro-mechanical properties as a function of strain on DS substrate with higher CNT loading in ECLME. a-b) $\Delta R/R_0$ and c-d) σ_c/σ_0 plotted as a function of tensile for printed and lasered ECLME layers on Dragon Skin® elastomer substrate. The filler loading of $eGa_{75.5}In_{24.5}$ was fixed to 65 vol.% while CNT loading varied from 1.5 vol.% to 2.25 vol.%. All σ_0 values were expressed as Siemens per centimeter ($S{\cdot}cm^{-1}$). The cross-sectional area decrease was not taken into consideration due to plausible non-homogenous stress distribution. VV, VH, HV, and HH denote the combined printing and lasering direction (V = vertical, and H = horizontal). In this case, vertical and horizontal mean along the length and width of the trace, respectively

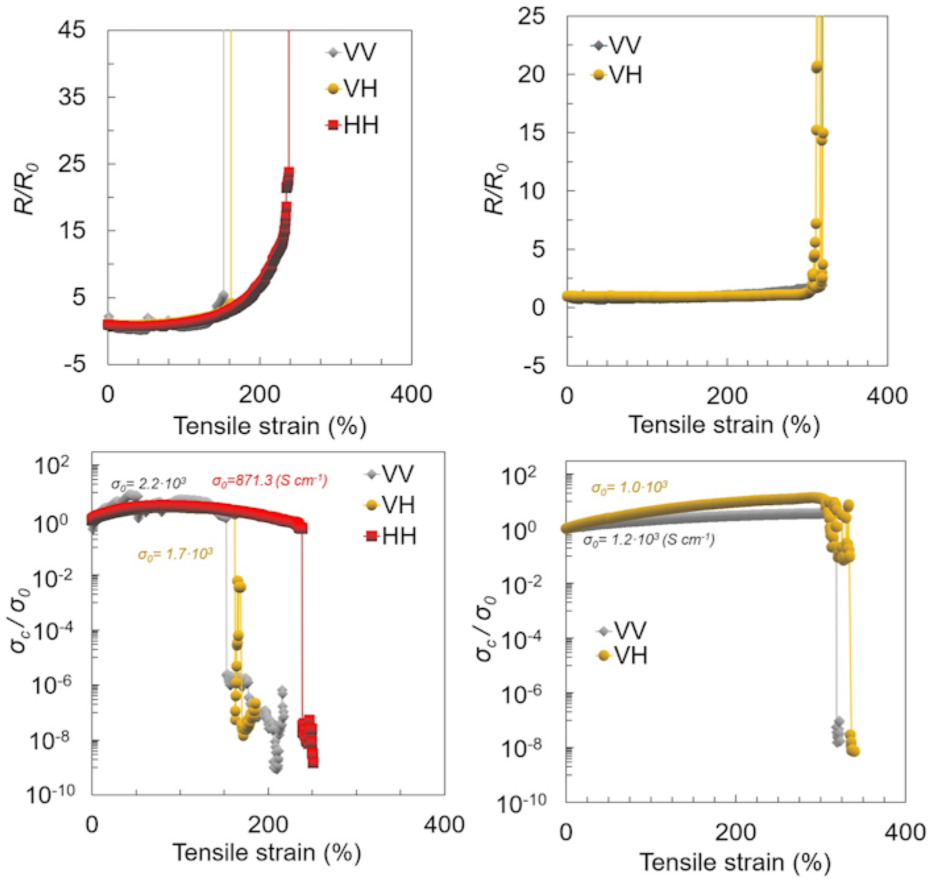

Figure S29. Electro-mechanical properties of ECLME on elastomer substrate with self-healing intermediate layer. R/R_0 and σ_c/σ_0 plotted as a function of tensile for printed and photothermally activated ECLME layers on Dragon Skin®/EC7-CNT (left) and VytaFlex®/EC7-CNT elastomer substrates (right). All σ_0 values expressed as Siemens per centimeter ($S{\cdot}cm^{-1}$). The cross-sectional area decrease was not taken into consideration due to plausible non-homogenous stress distribution. VV, VH, HV, and HH denote the combined printing and lasering direction (V = vertical, and H = horizontal). In this case, vertical and horizontal mean along the length and width of the trace, respectively

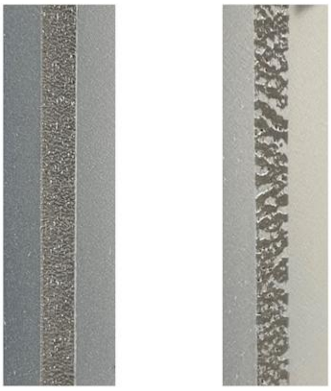

Figure S30. Photographs of ECLME and ECLME-HPC on DS substrate. ECLME with CNT (left) and ECLME with HPC (right) on Dragon Skin® elastomer under larger tensile strain. ECLME-CNT forms tiny microcracks which results in a significant increase of resistance. ECLME-HPC forms larger microcracks, but the LMMP regions can flow and reconnect the conductive pathways over the cracks.

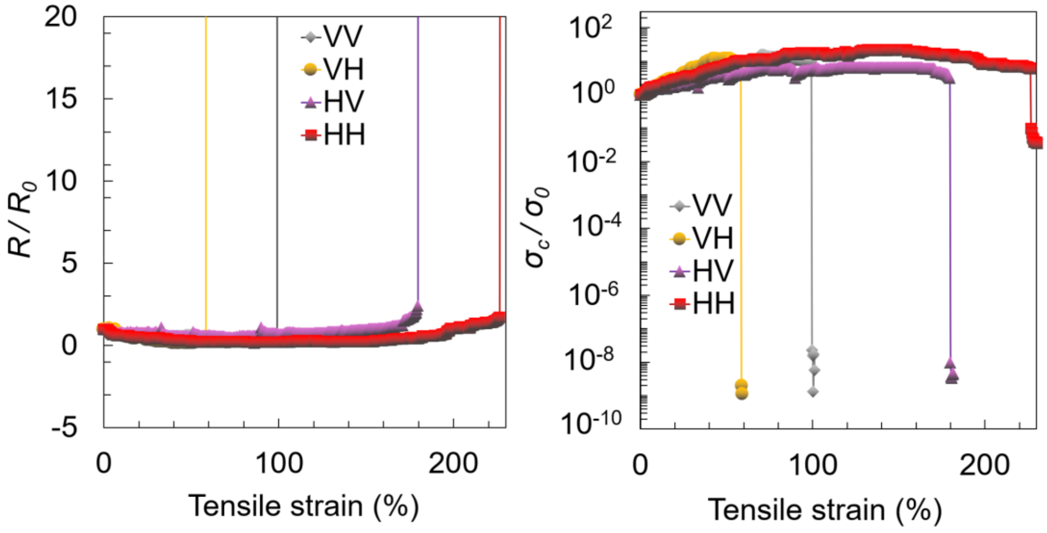

Figure S31. Electro-mechanical properties of ECLME-HPC on DS substrate. R/R_0 and σ_c/σ_0 plotted as a function of tensile for printed and photothermally activated ECLME-HPC layers on Dragon Skin® elastomer substrates. The cross-sectional area decrease was not taken into consideration due to plausible non-homogenous stress distribution. VV, VH, HV, and HH denote the combined printing and lasering direction (V = vertical, and H = horizontal). In this case, vertical and horizontal mean along the length and width of the trace, respectively

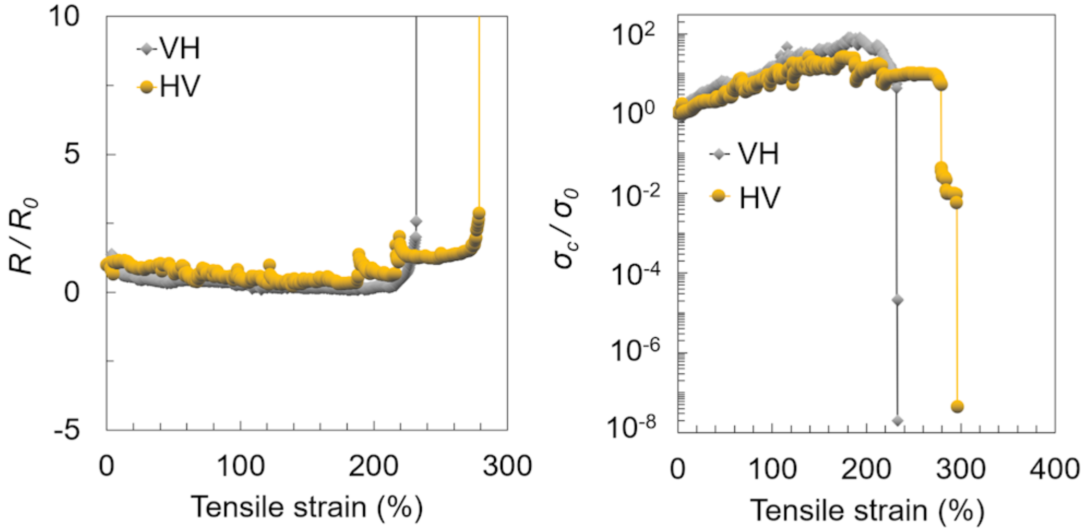

Figure S32. Electro-mechanical properties of ECLME-HPC on EC7 substrate. R/R_0 and σ_c/σ_0 plotted as a function of tensile for printed and lasered ECLME-HPC layers on EC7 elastomer substrates. The cross-sectional area decrease was not taken into consideration due to plausible non-homogenous stress distribution. VV, VH, HV, and HH denote the combined printing and lasering direction (V = vertical, and H = horizontal). In this case, vertical and horizontal mean along the length and width of the trace, respectively.

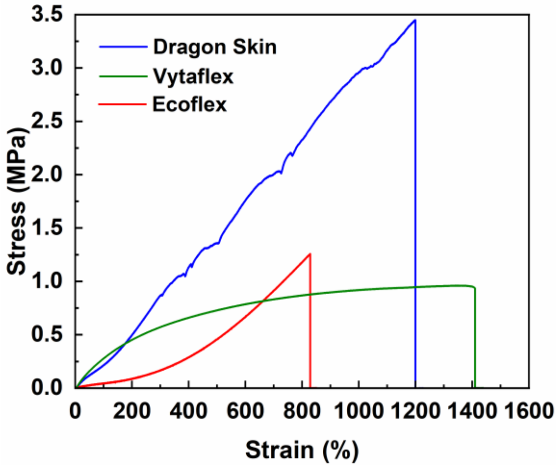

Figure S33. Mechanical properties of conventional elastomers. Tensile stress-strain curves measured for Dragon Skin®, VytaFlex®, and EcoFlex® at strain rate of approx. 0.65 % s^{-1}.

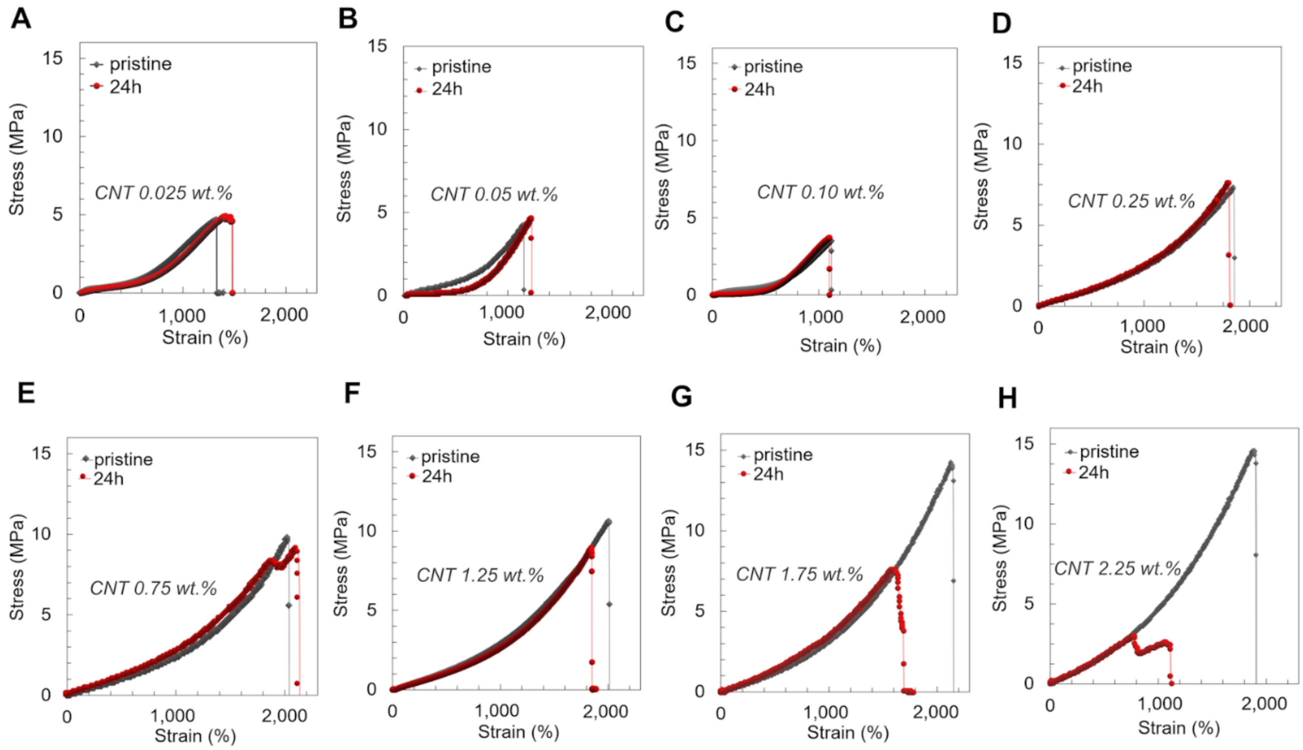

Figure S34. Tensile properties of EC7-CNT with varied CNT loading. a-h) The stress-strain curves for pristine and healed EC7 elastomer substrates with varied CNT loading from 0.025 to 2.25 wt.%. The samples were completely bisected after which the cut surfaces were aligned and allowed to self-heal for 24 hours before the measurements.

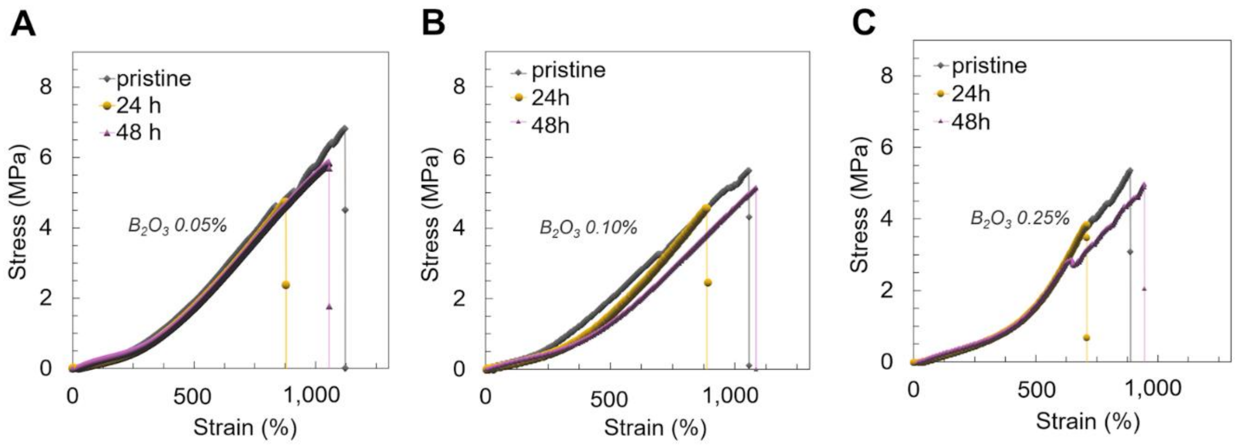

Figure S35. Tensile properties of EC7 with varied B_2O_3 content. a-c) Stress-strain curves for EC7 elastomer substrates with varied boron oxide nanoparticle (B_2O_3 NP) loading level (0.05 to 0.25 wt.%). The samples were completely bisected after which the cut-surfaces were aligned and allowed to self-heal for 24 and 48 hours before the measurements.

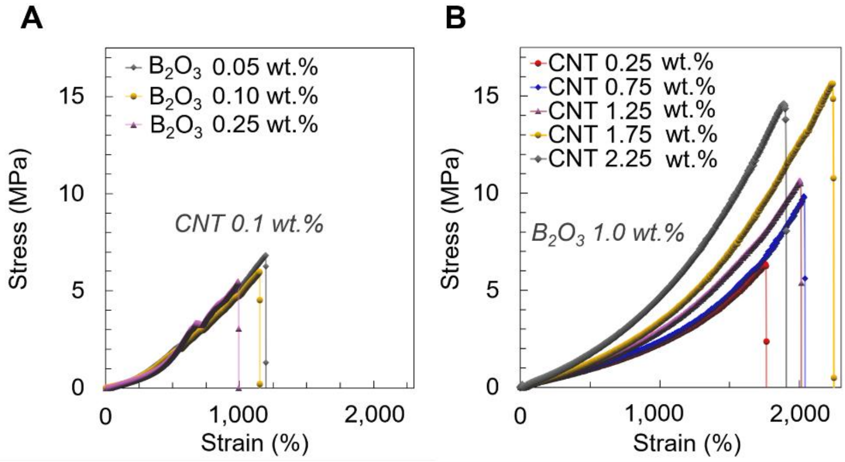

Figure S36. Tensile properties of EC7-CNT. Stress-strain curves for EC7 elastomer substrates with a) fixed CNT loading (0.1 wt.%) when the amount of B_2O_3 NPs were varied from 0.05 to 0.25 wt.%, and b) with fixed B_2O_3 NP loading (1.0 wt.%) when the amount of CNT was varied from 0.25 to 2.25 wt.%.

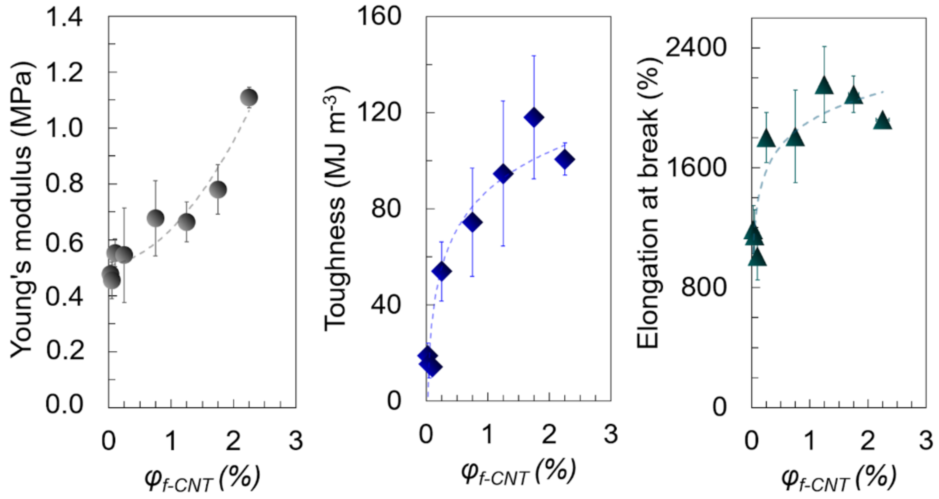

Figure S37. Mechanical properties for EC7-CNT. Median values for Young's modulus (in MPa), toughness (in MJ m^{-3}), and elongation at break (in %) plotted as a function of MWCNT-COOH volume loading (denoted as $\varphi_{\text{f-CNT}}$(%)) for EC7-CNT elastomer substrate. Error bars show the standard deviation (n ≥ 3).

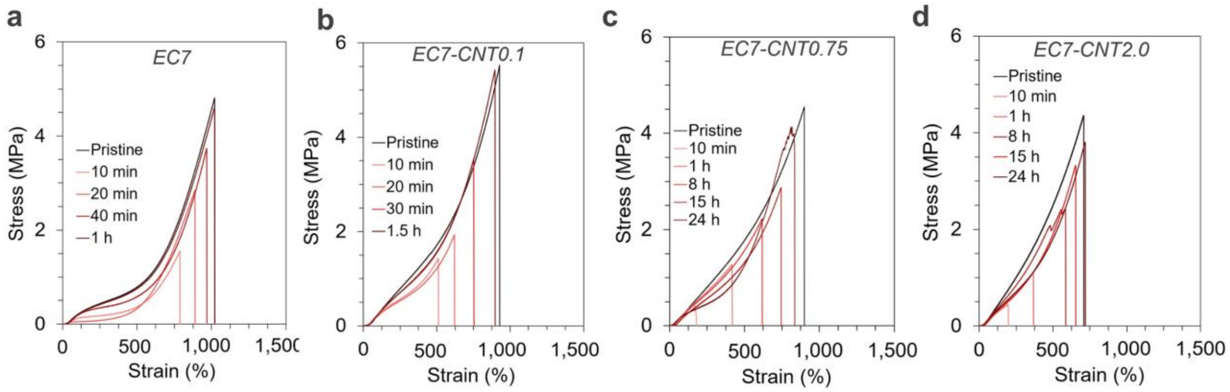

Figure S38. Mechanical self-healing for EC7 and EC7-CNT substrates. Stress-strain curves for pristine and self-healed EC7, EC7-CNT0.1, EC7-CNT0.75, and EC7-CNT2.0 substrates (without and with 0.1, 0.75, and 2.0 wt.% CNT). The self-healing time after the initial cut surface alignment varied from 10 minutes to 24 hours (depending on the composition).

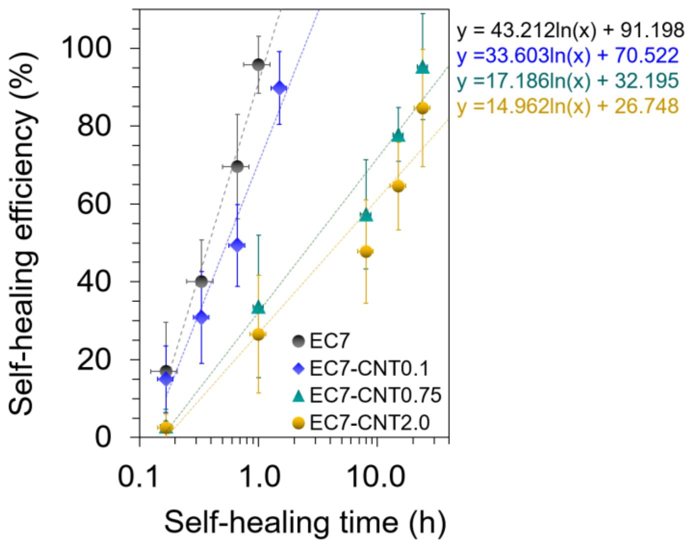

Figure S39. Self-healing efficiency for EC7 and EC7-CNTs. Self-healing efficiency (i.e., recovery of toughness (MJ m^{-3})) plotted as a function of self-healing time (in hours) for EC7, EC7-CNT0.1, EC7-CNT0.75 and EC7-CNT2.0 substrates (without and with 0.1, 0.75, and 2.0 wt.% CNT). Data plotted as mean ± STD ($n \geq 3$).

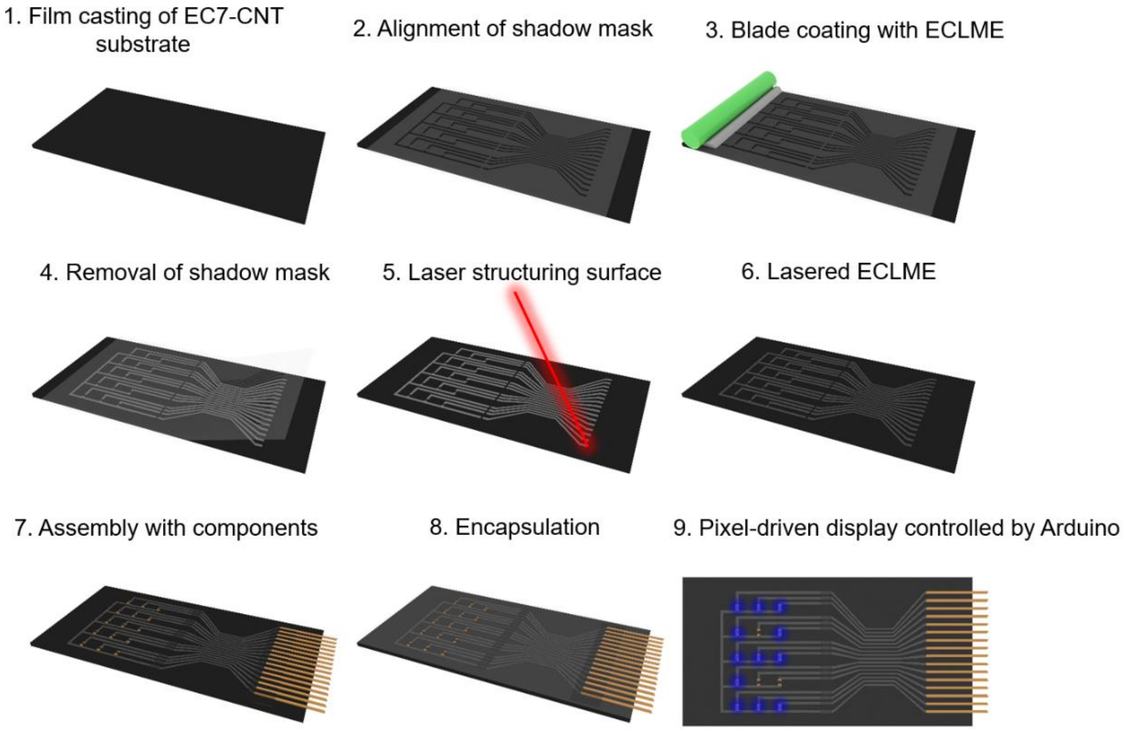

Figure S40. Schematic illustration of the display fabrication. The stretchable and self-healing display fabrication process consists of film casting, patterning with blade coating using a shadow mask, laser structuring patterns for photothermal activation, assembly and bonding of the surface-mountable components to the ECLME interconnections via built-in self-healing functionality and pressure-activated adhesive properties.

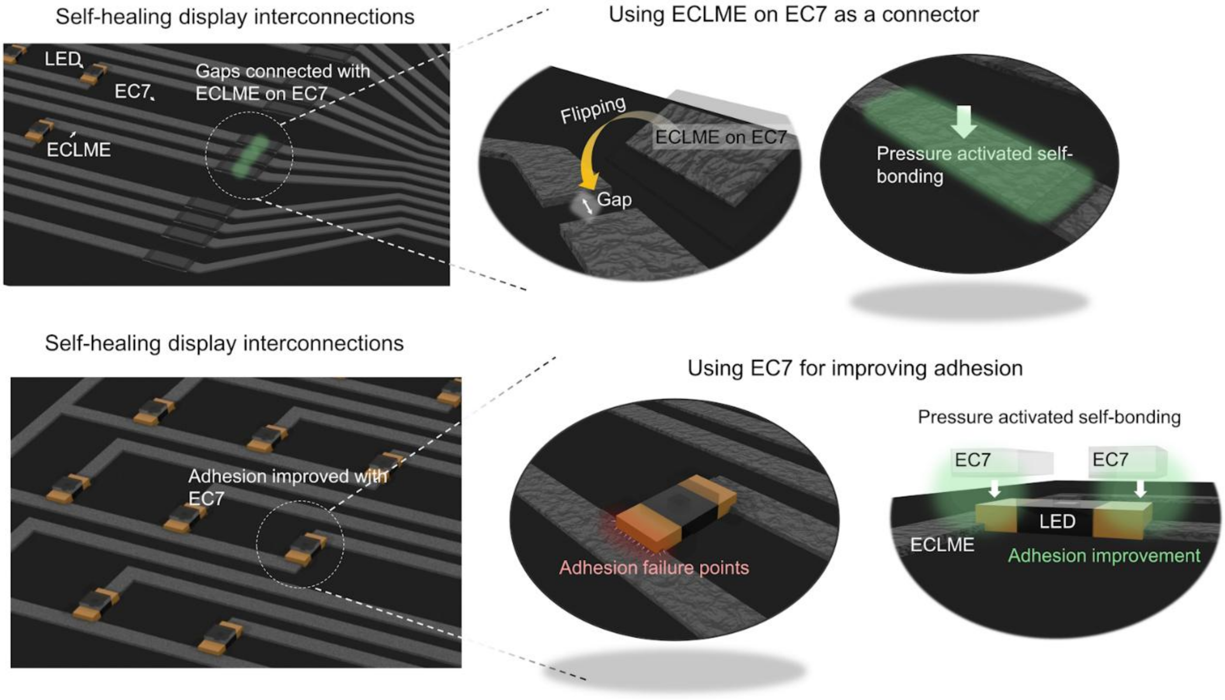

Figure S41. Schematic illustration on interconnections and bonding of components. The illustration shows how electro-mechanical contact was formed between two distinct parts of the interconnections. The underwater adhesion could be improved with EC7, or EC7-CNT, by placing it between surface-mountable light emitting diodes and ECLME interconnection in the self-healing display used for underwater demonstration.

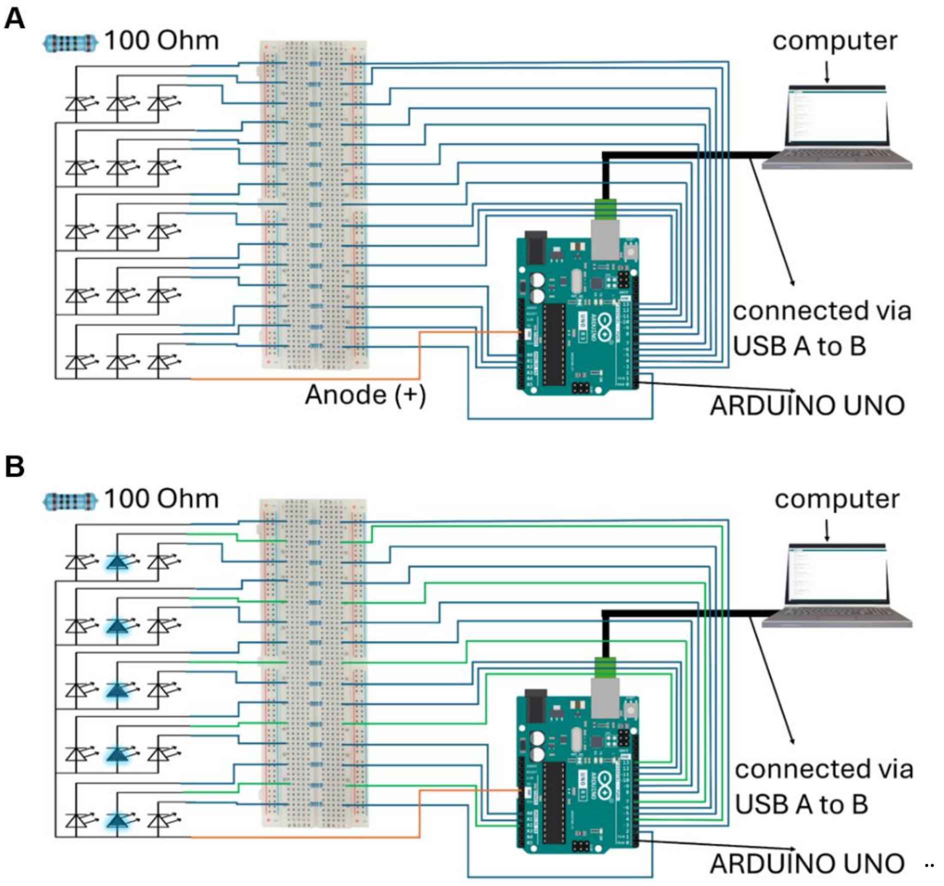

Figure S42. **Schematic illustration of the electronics for controlling the display.** The self-healing display was electrically connected to a breadboard with 100 Ω resistors that were further connected to Arduino UNO microcontroller. Computer was connected to the microcontroller via USB A to B cable.

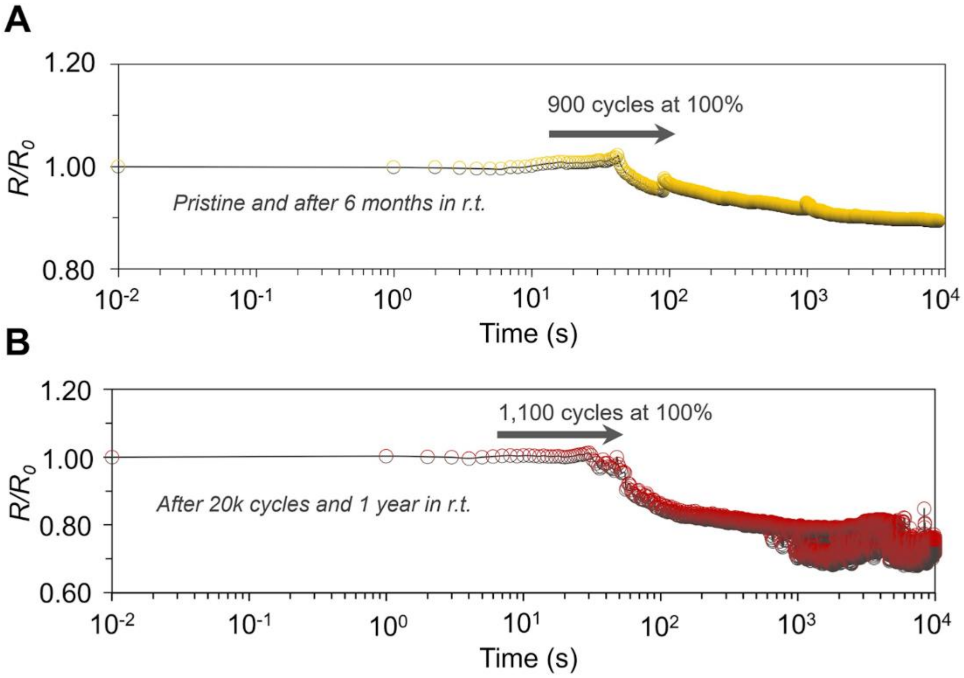

Fig. S43. Electro-mechanical stability after aging. R/R_0 measured as a function of time for 900 – 1,100 stretch-release cycles at ε = 100% for ECLME interconnections on EC7-CNT substrates. a) Pristine ECLME interconnection on EC7-CNT elastomer after aging in room temperature (r.t.) for 6 months after the printing and photothermal activation. b) The cycled ECLME interconnection on EC7-CNT (shown in Fig. 4f), which was originally subjected to 20,000 stretch-release cycles, was remeasured after aging for 1 year at r.t.

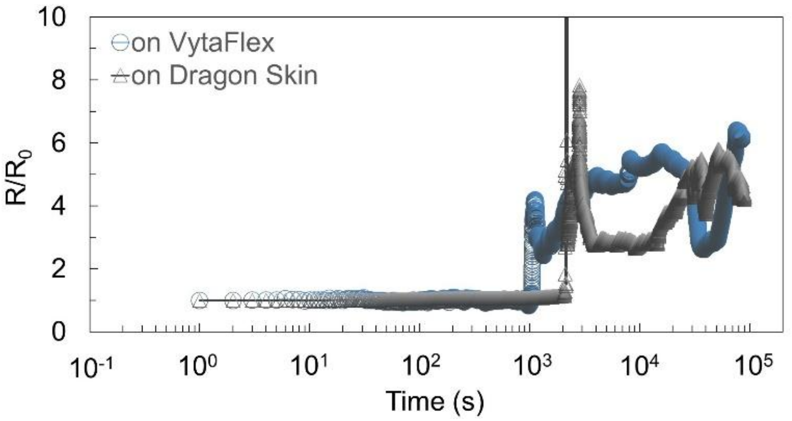

Figure S44. Underwater stability of ECLME on conventional elastomers. R/R_0 measured as a function time for underwater immersion of ECLME printed onto VytaFlex® and Dragon Skin® elastomer substrates.

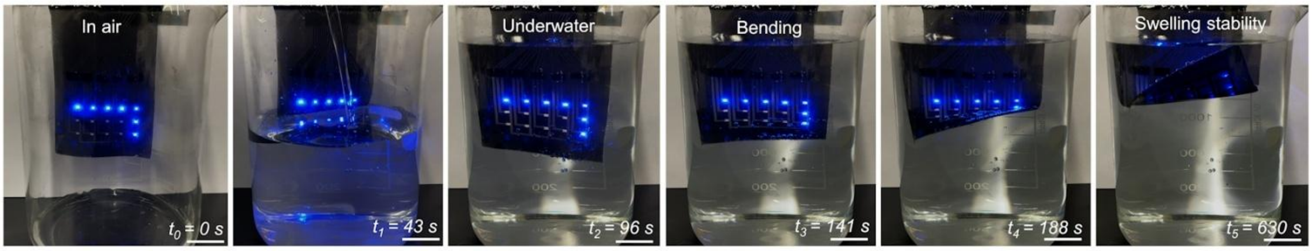

Figure S45. Photographs of self-healing display underwater. Initially, the freestanding display was tested by placing it under water. Due to anisotropic swelling behaviour of the material structure, the display started to bend (at t_3 = *141* seconds) after complete underwater immersion. Despite the underwater bending, the display was still completely functional (t_5 = 630 s). Scale bars 20 mm.

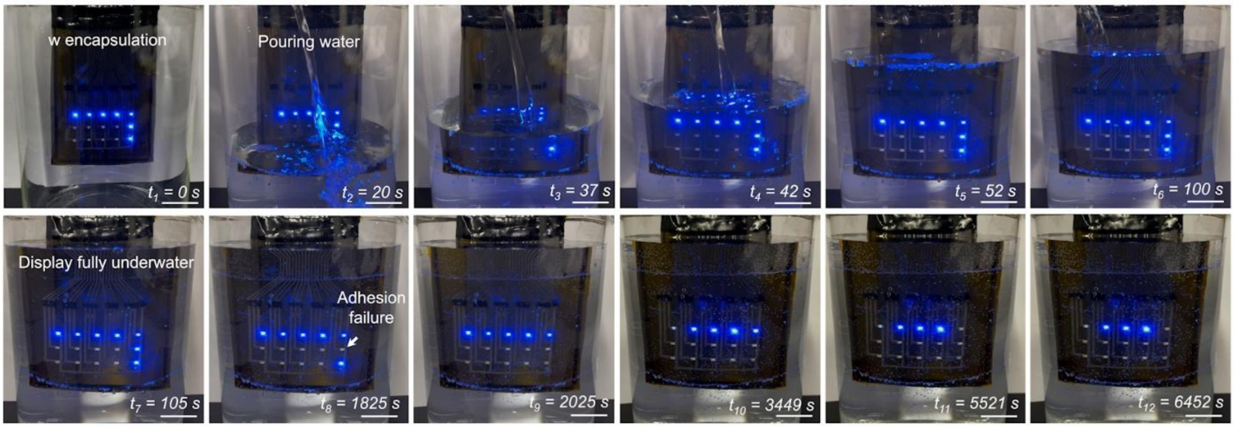

Figure S46. Photographs of the self-healing display underwater. A supporting wooden frame was attached to the edges of substrate with Kapton tape to prevent underwater bending of the display. Without additional EC7 adhesives (illustrated in Figure S40), the display lost its original functionality (at t_8 = 1825 s) as a single LED stopped working. Three additional LEDs stopped working at t_9 = 2025 s, t_{10} = 3449 s, and t_{11} = 5521 s. During longer underwater immersion, we found that the display completely stopped working due to improper underwater adhesion at the ECLME-interconnection/SMD LED interfaces. Scale bars 20 mm.

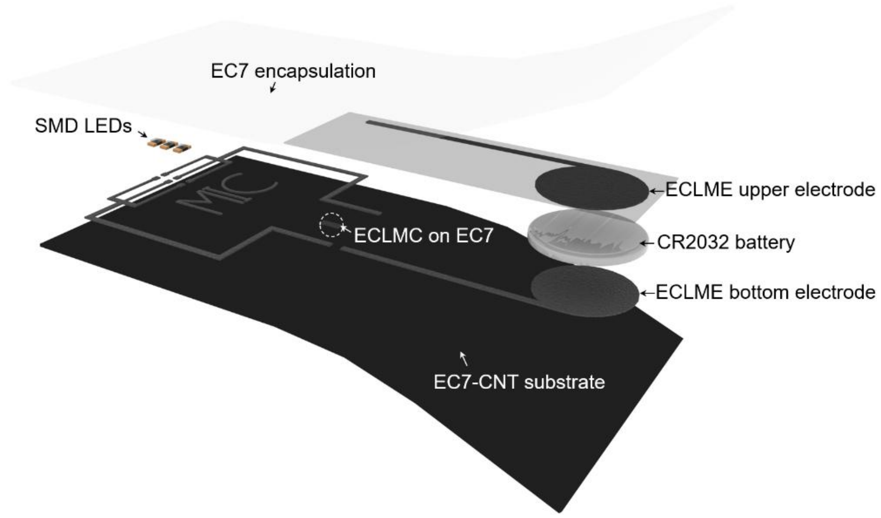

Figure S47. Schematic illustration of ECLME-based battery-integrated LED demonstrator structure. The structure consists of substrate with printed ECLMEs to which surface-mountable components and CR2032 battery were placed. The electrical connections to the integrated battery are made by manually aligning the second ECLME electrode structure to connect the upper battery electrode to the printed ECLME on the substrate. The electro-mechanical connection between the layers was automatically formed via self-healing when the surface was placed in physical contact.

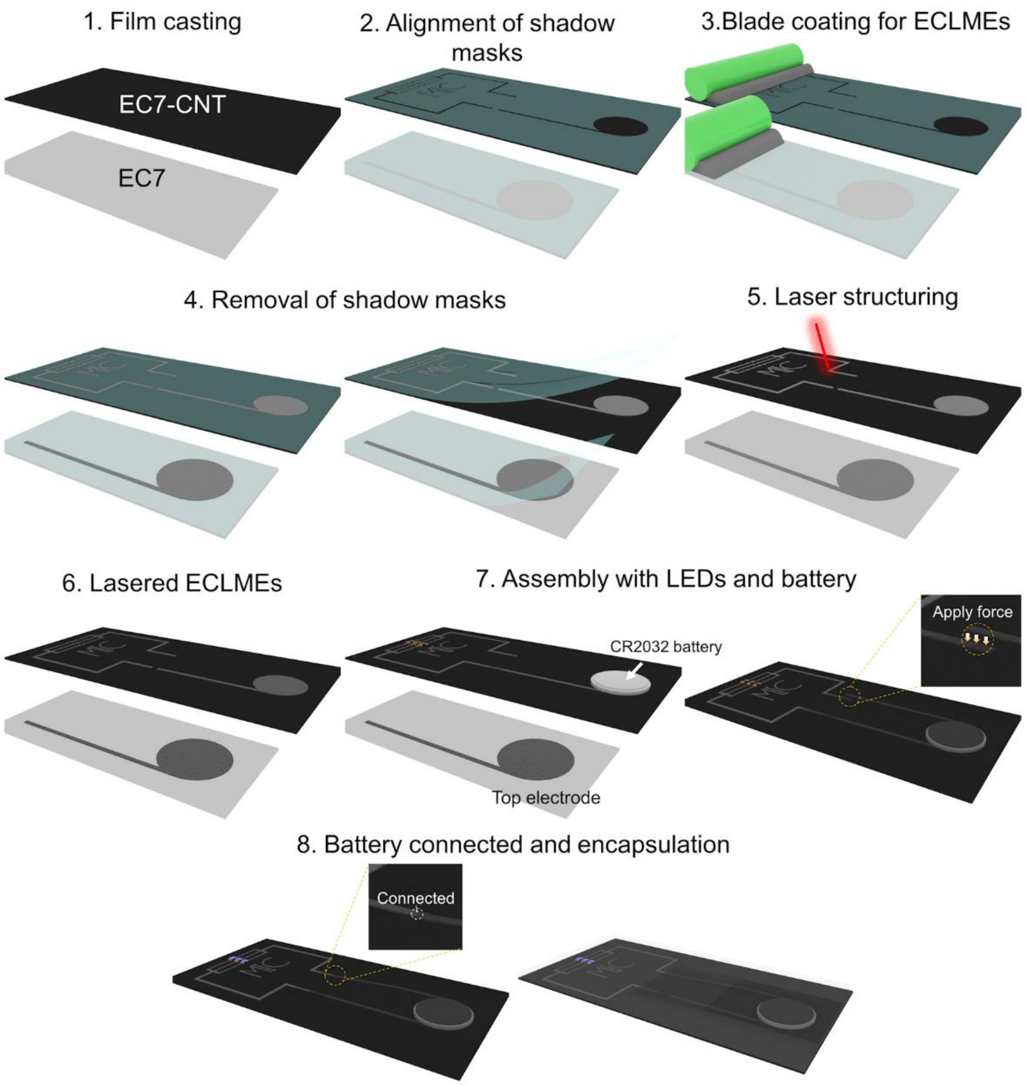

Figure S48. Schematic illustration of the fabrication process of battery-integrated device. The fabrication process for ECLME-based battery-integrated LED demonstrator structure consists of film casting, blade coating with shadow mask, lasering film surfaces, and assembly of the device by using common CR2032 battery, SMD LEDs, and upper ECLME electrode.

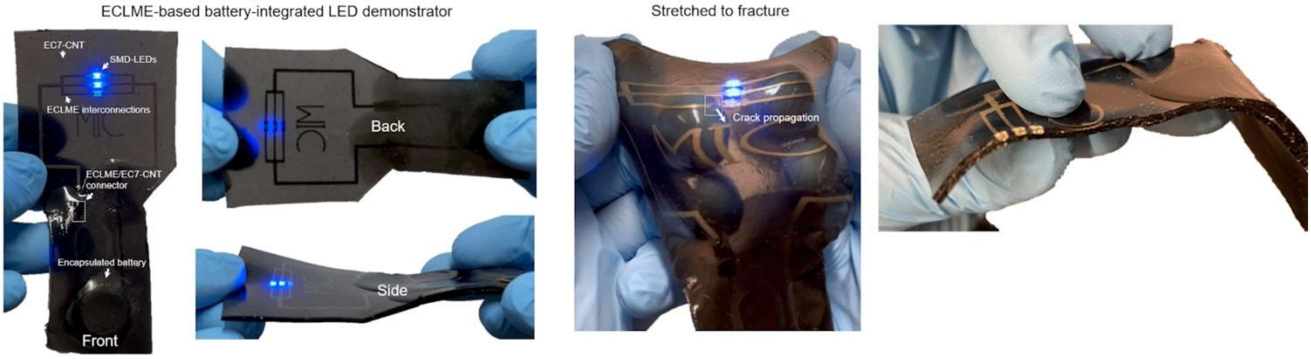

Figure S49. Photographs of the battery-integrated LED demonstrator. Photographs of the device from front, back, and side profile. The broken device was stretched to break by both stretching and pushing the substrate upward with fingers. The self-healing material structure fractures at the edge of the LED. After the fracture, no leaking of LMMPs were observed.

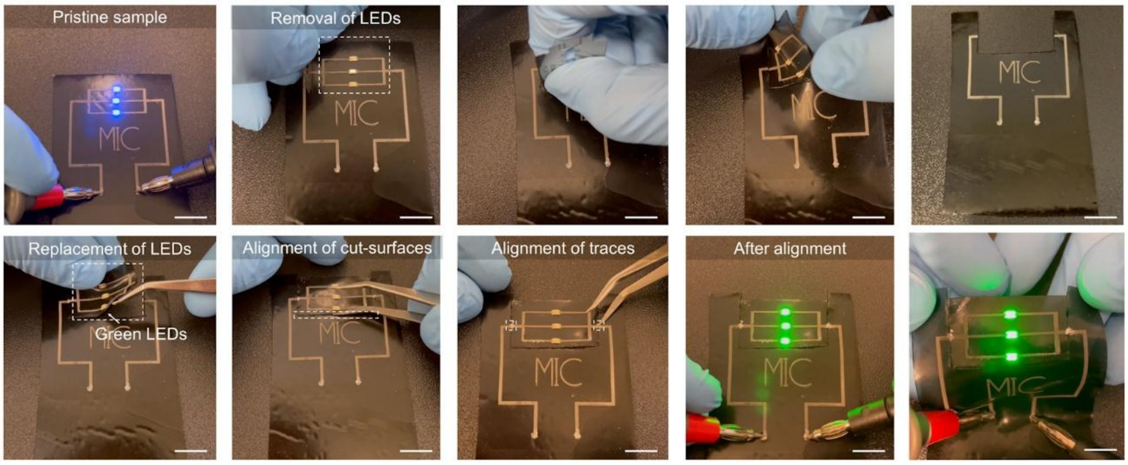

Figure S50. Photographs of LED replacement. A surface mountable blue LEDs from ECLME-based interconnection structure were replaced with green LEDs after removal of a piece. The electro-mechanical connection was reformed after the alignment via built-in self-healing of the materials. Scale bars 10 mm.

Table S1. Comparison table for the state-of-the-art liquid metal-based stretchable conductors. Criteria that are fully met are highlighted in green, while partially and incompletely met criteria are indicated in orange and red, respectively. Please note that the colour coding is only used for visual clarity.

Conductive material	Maximum σ_c under ε ($S{\cdot}cm^{-1}$)	R/R_0 at working range	Layer thickness	Resolution	Self-healing functionality	Build-in adhesive properties	Processing method	Demo(s)	Ref.
eGaInPs (~1100 nm) mixed with pp-TPU particles to form ink	22,535	~ 2.7 at ε = 1,000%	~30 µm	~ 200 µm; stencil printing with masks	Scratch healing	No; distinct adhesive material used	Ultrasonication for eGaInPs and physical mixing with TPU to prepare the ink	Non-self-healing display controlled with pressure sensors; thermochromic heaters	[S1]
LMMP/AgNW ink	2,620	~20 at ε ~ *400%*	~ 20 µm	~ 42.3 µm	No	No; commercial adhesive used	Ultrasonication for LMMPs; vacuum filtration, laser sintering and etching for the ink	Temperature or pressure sensor component bonded to interconnection	[S2]
PVDF-HFP-TFE mixed with LM dispersion	435	< 3.5 at ε ≈ 380 – 530%	N/A	N/A	No	N/A	Wet spinning for the solution to prepare microfibers	Triboelectric fibers for stretch sensor	[S3]
Biphasic eGaIn (~500 nm) ink	20,600	< 1.0 at ε < 500%	~ 50 µm	~25 µm; spraying coating with laser-cut masks	No	Yes	Sonication, spray-coating onto silicon wafer before heating at 900 °C for 30 min.	Small scale sensing circuit with surface mountable components	[S4]
Bulk eGaIn	18,000	< 1.5 at ε < 1,800%	N/A	~ 500 µm	No	No	Bulk LM stencil printed onto electrospun SBS mat, and activated with stretching	LED-array, and ECG electrode	[S5]
LM-SIS microcapsule /copper layer	18,000	~ 10 at ε < 550%	~ 25 µm	~ 50 µm	Crack healing by rupture of microcapsules	No	LM mixed with SIS screen printed, and copper layer deposited on top	Non-self-healing, bendable 5 by 5 display; electrode patch with heaters	[S6]
eGaIn/Ag flakes/SIS ink	8,210	< 6 at ε < 400%	~ 60 µm	> 200 µm	Scratch healing	No; vapor exposure used for the bonding	Planetary mixing and extrusion printing for the ink	Non-self-healing wireless and wearable temperature patch with LED display	[S7]
eGaIn/TPU slurry	21,300	< 4 at ε < 300%	~ 20 µm	~ 100 µm	No	No	Sonication, and electrospinning of the slurry. Activated with pressure-stamping	Small timer circuit with single LED	[S8]
eGaIn-PU	12,000	< 2 at ε < 500%	~ 80 µm	N/A	No	No	Drop casting to aluminum foil, and laser cutting	ECG electrodes	[S9]
AgNPs/Cu/LM layer	41,500	< 8 at ε < 300%	N/A	~ 15 - 100 µm	No	No	Shadow mask patterning for growing AgNPs, deposition of Cu, and coating LM / photolithography	Stretchable LED matrix	[S10]
PUA + Ag + + liquid metal	6,250	~2 at ε < 1500%	~200 µm	N/A	96% recovery of electrical conductivity	No	Ultrasonication for eGaInPs and physical mixing with PUA and Ag flakes to prepare the ink, then drop cast and UV cured	Stretchable conductor for triboelectric nanogenerators	[S11]
eGaIn/CNT/elastomer ink	> 20,000	< 3.95 at ε < 500%	~25 µm	~40 µm	Autonomous self-healing after completely bisected	Yes	Ultrasonication, blending, and printing for the ink. Photothermal activation	Stretchable, self-healing, and waterproof LED-display	**This work**

Table S2. Comparison table for the state-of-the-art autonomous self-healing conductors. Criteria that are fully met are highlighted in green, while partially and incompletely met criteria are indicated in orange, and red, respectively. Please note that the colour coding is only used for visual clarity.

Material	Tensile properties			Self-healing properties		Curing Temp. (℃)	Electrical conductivity	Applications	Ref.
	σ_b (MPa)	U_T (MJ m^{-3})	ε_b (%)	η (%)	conditions				
PEDOT:PSS/PIL elastomers.	0.0795	4.85	104.41	~80 for conductivity	24 h in r.t.	70 °C for 24 h	~0.006 Sm^{-1}	Electronic devices and sensors Human motion detection and stress-sensing devices	[S12]
PTBB-Zn elastomer	1.73	4.28	400	93.1 for conductivity	60 °C for 24 h	vacuum oven for 12 h at 60 °C	N/A	Real-time monitoring of hand gestures Hand rehabilitation training	[S13]
ACPM-type conductive elastome	15.7	125	2400	99 for conductivity	24 h in r.t.	Using ultraviolet light curing setup	0.028 Sm^{-1}	Human motion detection Health monitoring	[S14]
P(BMA-co-MEA) Liquid-Free ICEs	1.32	1.08	1913	~100 for conductivity	1 h in r.t.	UV light (365 nm, 36 W) irradiation for 1 h	135 Sm^{-1}	Wearable sensors for detecting human body movements Self-healing soft ionotronic devices for mechanical breakdown	[15]
Ionic PEGgel (IHP)	1.18	7.16	1400	68 for conductivity	After 1 h in r.t.	30 s under 5w cm^{-2} UV light (366 nm)	0.04 S m^{-1}	Flexible sensors for strain or temperature sensing Skin electrodes for recording electrocardiograms	[S16]
PDES elastomer	0.275	3.15	1464	55 for conductivity	Within 12 h in r.t.	UV light source for 15 s	3.2 S m^{-1}	Detecting human activities with high sensitivity. wearable devices	[S17]
PAAN hydrogels	0.9	7.85	2590	92 for conductivity	24 h in r.t.	t 60 °C for 6 hours	3.35 S m^{-1}	human physiological monitoring electronics, flexible touch screens, and artificial electronic skin	[S18]
SA/PVA-Fe^{3+} elastomers	38.6	N/A	1565	53.4 for conductivity	In r.t.	50 °C	1481.5 S m^{-1}*	Health care applications Electrocardiography and safety monitoring	[S19]
ECLME ink on EC7-CNT elastomer	> 10	> 95 - 120	> 2090 – 2150	≥ 100 for electrical conductivity, and electro-mechanical property	After 24 in r.t.	50 - 70°C	> 2,000,000 S m^{-1} under strain	Electrical wiring, interconnections, electrodes and conductive structures for soft self-healing and strain-insensitive electronics	**This work**

σ_b, U_T, ε_b, and η denote stress at break, toughness, elongation at break, and self-healing efficiency, respectively.

Table S3. Tensile and self-healing properties for EC7 and EC7-CNTs. Uniaxial tensile properties of self-healing films (used as the substrate) with varied carboxylic acid-functionalized multiwalled carbon nanotube and boron oxide nanoparticle filler loading levels. The tensile and self-healing properties were measured at r.t.

Composition	Young's modulus, *E* (MPa)	Elongation at break, ε_b (%)	Toughness, U_T (MJ m^{-3})	Self-healing efficiencies U_T%		
				24h (%)	48h (%)	72h (%)
EC7(B.05)-CNT0.1	0.540 ± 0.057	1137 ± 88	21.34 ± 8.39	55.4 ± 12.7	88.7 ± 13.8	-
EC7(B.10)-CNT0.1	0.581 ± 0.134	990 ± 175	21.38 ± 7.11	54.4 ± 7.6	82.9 ± 11.5	-
EC7(B.25)-CNT0.1	0.471 ± 0.087	934 ± 145	17.14 ± 9.76	52.3 ± 3.2	104.2 ± 30.8	-
EC7-CNT0.025	0.477 ± 0.032	1187 ± 161	18.89 ± 5.22	94.0 ± 37.6	-	-
EC7CNT0.05	0.455 ± 0.066	1147 ± 171	15.41 ± 5.86	96.1 ± 27.7	-	-
EC7-CNT0.10	0.553 ± 0.049	1010 ± 158	14.23 ± 3.51	99.6 ± 22.0	-	-
EC7-CNT0.25	0.545 ± 0.169	1802 ± 167	53.93 ± 12.29	100.7 ± 16.5	-	-
EC7-CNT0.75	0.677 ± 0.134	1810 ± 309	74.31 ± 22.52	97.0 ± 8.5	-	-
EC7-CNT1.25	0.664 ± 0.071	2156 ± 252	94.58 ± 30.20	89.4 ± 0.9	-	-
EC7-CNT1.75	0.780 ± 0.088	2091 ± 122	118.02 ± 25.63	41.4 ± 0.4	N/A	N/A
EC7-CNT2.25	1.019 ± 0.037	1920 ± 36	100.61 ± 6.73	17.4 ± 1.1	N/A	N/A

EC7 denotes an elastomer composition 7 (i.e., C7; please see the Supplementary Information in 10.1002/advs.202103235 (Page 8, Table 2) for more information)

The amount of boron oxide nanoparticles (B_2O_3) denoted as B.05, etc. (corresponding to 0.05, 0.10, and 0.25 wt.% of B_2O_3 NPs). The amount of carbon nanotubes (CNTs) was fixed to 0.10 wt.%.

The amount of CNTs denoted as CNT0.025, etc. (corresponding to 0.025 – 2.25 wt.% of CNTs). The amount of B_2O_3 NPs was fixed to 1.0 wt.%

Values expressed as a mean +/- STD ($N \geq 3$).

Table S4. Comparison table for the state-of-the-art of self-healing elastomers. Criteria that are fully met are highlighted in green, while partially and incompletely met criteria are indicated in orange, and red, respectively. Please note that the colour coding is only used for visual clarity.

Material	Mechanical properties			Self-healing properties		Curing Temp. (℃)	Electrical properties	Applications	Ref.
	σ_b (MPa)	U_T (MJ m^{-3})	ε_b (%)	η (%)	conditions				
PDMS-DAP@Fe	2.81	32	1431	95	70 ℃ for 12 h	80	Conductive when sprayed with AgNPs	Substrate for flexible electronic devices. self-healable materials.	[S20]
H-PDMS-Py_m Elastomers	7.46	79.89	2174	96	110 ℃ for 1h	N/A	insulator	Smart flexible sensors or devices. Healable PDMS-based substrates for long-term applications.	[S21]
PDMS-g-UPy	0.483	N/A	1513	76	60°C for 2 h	N/A	insulator		[S22]
Fe−Py−PDMS/PDMS−FBA elastomer	1.1	7.2	882	~96	70 °C for 12 h	80 °C	insulator	various practical applications, such as remote anti-icing/deicing materials.	[S23]
$PDMS_3$-$IPDI_4$-SS-F-M	2.4	13.6	988	92	65°C for 24 h	60°C	Conducing when depositing a conductive filler onto the surface	Flexible electronic devices Sustainable flexible support materials	[S24]
PDMS-TFB	N/A	N/A	120	98.3%	room temperature for 1 h	In vacuum for 12 h	insulator	Anticorrosion coating and adhesive layer use in adhesives and encapsulants. protect conductors and circuits	[S25]
PDMS−TDI−Al	2.6	14.7	1700	90	24 h of healing under ambient conditions	80 °C for 12 h	Conducing when depositing a conductive filler onto the surface	Electronic skin Soft robotics Electrical devices	[S26]
supramolecular polyurethane elastomers (SPUEs) PU–BN_{11}	10.5	182.2	3120	31.1	Room temperature for 20 h	80°C for 48h	Conducing when depositing a conductive filler onto the surface	soft protective armor and wearable flexible electronics that can heal after routine damage.	[S27]
TA-WS2/PU composites	52.3	282.7	1020.8	80–100	at room temperature for 12 h	Room temperature	insulator	flexible functional devices	[S28]
DxAyWPU	33.04	90.66	954.79	90.1	80 °C for 6 h	Room temperature	Conducing when depositing a conductive filler onto the surface	wearable flexible strain sensors	[S29]
$PU_{3.8\text{-}80/20}$	34.1	127.3	2014	83	Room temperature for 48 h	Room temperature	insulator	protective film on the surface of woodware and metalware	[S30]
PDM elastomers	29.0	121.8	1806	~100%	Room temperature for 24 h	80°C	insulator	self-healing macromolecules in puncture-resistant tire sealants, tough and resilient sportswear materials, and in durable protective coatings	[S31]
DMG-PUEs	34.5	53.9	538	~ 100%	70 °C for 24 h	80 °C	insulator	Flexible displays Solar cells	[S32]

PAU-HBD elastomers	21.82	87.1	1601	58	underwater	60 ℃ vacuum oven for 48 h, followed by hot-pressing at 120 °C for 3 min to obtain a thin elastomer film	insulator	Ocean explorations Underwater operations	[S33]
EC7-CNT	> 10	> 95 – 120	> 2090 – 2150(elastic region)	>90 – 100 after 1-48 hours	Room temperatur e	Room temperature, 0.5 – 48 hours	Conductive when blended PEDOT: PSS or LMMP/CNT	Substrate for self-healing soft electronics, and base material for various functional materials	**This work**

σ_b,U_T, ε_b, and η denote the stress at break, toughness, elongation at break, and self-healing efficiency (calculated from toughness), respectively.

Table S5. Tensile properties for ECLME. Uniaxial tensile properties of non-photothermally activated freestanding films of ECLME measured at r.t.

Composition	Young's modulus, E	Elongation at break, ε_b	Toughness, U_T
	(MPa)	(%)	(MJ m^{-3})
EC7-LM65-CNT0.75	1.099 ± 0.100	296 ± 33	3.466 ± 0.823

Table S6. Comparison table for the stretchable displays. Criteria that are fully met are highlighted in green, while partially and incompletely met criteria are indicated in orange, and red, respectively. Please note that the colour coding is only used for visual clarity.

Display type	Electrode structure	Control method	Pixel density	Physical size *(mm x mm)*	Deformability	Self-healing functionality	Operational stability	Underwater stability	Integration method	Reference
Micro-LED display	Au/graphene electrodes, and commercial elastomer	Individual pixels can be controlled with external circuit board (Raspberry Pi 4B) and Python program	7 x 7	22 x 22	100% uniaxial stretching	No	Interconnections stable for 50 stretch-release cycles. Pixel brightness uniformity 1.1% (< 530 cd m^{-2}) under strain	No	Transfer patterning functional structures onto pre-stretched elastomer	[S34]
LED display		Individual pixels can be controlled with external circuit board	6 x 6	N/A	50% uniaxial stretching, and twisting	No	Forward voltage difference of LED matrix stable for 6,000 cycles at 30% strain	No	Commercial conductive adhesive (H20E, EPO-TEK), and optical adhesive (N0A61, Norland Products) used	[S35]
LED array	Solution processed liquid metal	LED array was powered by a HYELEC DC power supply	3 x 4	N/A	525% area strain	No	The current is decreasing as the strain increasing as a result of increasing the resistance	No	LED chips (LTW-216TS5, 1206, Lite-On Inc.) were positioned on the contact pads and gently pressed with a tweezer for reliable electrical connections	[S36]
SOLED display module	Ag (30 nm)/MoO3 (5 nm)/NPB (65 nm)/Bebq2:Ir(piq)3 (70 nm, 8 wt%)/Liq (1 nm)/Al (100 nm)	the OLEDs were voltage-driven by a source meter (Keithley 2400, Keithley Inc.), which also measured the current	3 x 3	N/A	95% strain	No	100,000 stretch-release cycles at 50% strain	Up to one month	OLED fabrication by thermal evaporation on PET substrate	[S37]
stretchable display	PVP/EGaIn	Microcontrollers mounted on the display	12 x 12	N/A	220% strain	No	Interconnection stable for 2,000 cycles under 200% strain. Ratio of surviving LEDs 100% for 500 cycles at 100% strain	No	older paste (Sn42Bi58, Ausbond, China) on copper contacts on e-vests	[S38]
stretchable LED ldisplay	liquid metal nanoparticle ink	The LEDs were controlled individually by an external microcontroller (Arduino Uno).	5 x 5	N/A	Diagonal stretching	No	Interconnection stable for 1,500 cycles at 100% strain	No	33 LEDs (Kingbright, 0603) were pressed onto the VHB substrate at the designated positions and held for 3 min to encourage adhesion.	[S39]
stretchable LED display	LM electrode (EGaIn/Ni)	Through a driving board connected to the display through FPC connector	32 x 32	64 x 64	100% uniaxial strain 50% 3-axis strain	No	12 000 cycles and more than 7200 h of reliability	underwater for several minutes	mini-LEDs were assembled directly onto the pixel using a standard pick-and-place SMD mounter	[S40]

									(LM900, FRITSCH) after being transferred on a stretchable substrate.	
LED Array	eGaInPs in TPU	The LED array was powered by a voltage of ≈2.9 V.	4 x 5	N/A	50% strain	No	Interconnection stable for 12,000 cycles under 50% strain (less than 1% change of resistance)	No	LEDs placed on the conductor, and pressed for 3 minutes, and encapsulated with lamination	[S41]
printed self-healing stretchable electrode integrated with LED light i	LM/Ag FDs/SIS	Individual pixels cannot be controlled	1 x 3	N/A	Not indicated	Yes. Self-healing of the 1x3 LED device shown under stretching.	Interconnection stable for 1000 cycles at 100% strain	No	LEDs placed on the conductor, and encapsulated	[S42]
Micro-LEDs Display	LM interconnects		4 x 4	9 x 9	Biaxial, 24%	No	Biaxial stretching for 100 000 cycles at 24% strain for 16 interconnections	No	SAC solder with lift-off technique, and flux material spin coated.	[S43]
LED display	ECLME ink	Individual pixels can be controlled with external circuit board (Arduino)	3 x 5	54 x 54	> 200% uniaxial stretching	Autonomously self-healing electrode structure and substrate. Self-healing of the display shown under stretching.	Interconnection stable for 20,000 cycles at 100% strain with < 4% change of resistance	Underwater stability up to 4 months	Direct bonding to ECLME without need for any adhesives	**This work**

Captions to Supplementary Movies:

Movie S1.
Bisecting the display with the razor and aligning the cut-surfaces for self-healing. Video speed x1.5.

Movie S2.
The display after self-healing for 24 hours at room temperature. Video speed x1.5.

Movie S3.
Stretching the display after self-healing for 24 hours at room temperature. Video speed x1.5.

Movie S4.
The display switching through numbers in dark and ambient lighting. Video speed x1.

Movie S5.
Stretching of the display. Video speed x1.

Movie S6.
Large stretching of the display. Video speed x1.

Movie S7.
Underwater bending of the display. Video speed x15.

Movie S8.
The display after underwater immersion for 1 hour. Video speed x85.

Movie S9.
Breakage of ECLME-based battery-integrated LED demonstrator. Video speed x1.

References

[S1] Chen, S., Fan, S., Qi, J., Qiao, Z., Wu, Z., Yeo, J. C., Lim, C. T., Ultrahigh Strain-Insensitive Integrated Hybrid Electronics Using Highly Stretchable Bilayer Liquid Metal Based Conductor, *Adv. Mat.,* **35**, 2208569 (2023)

[S2] Kim, M., Hong, S., Park, J. J., Jung, Y., Choi, S. H., Cho, C., Ha, I., Won, P., Majidi, C., Ko, S. H., A Gradient Stiffness-Programmed Circuit Board by Spatially Controlled Phase-Transition of Supercooled Hydrogel for Stretchable Electronics Integration, *Adv. Mat.*, 36, 2313344 (2024)

[S3] Zheng, L., Zhu, M., Li, Z., Sun, S., Wu, P., Conductance-stable liquid metal sheath-core microfibers for stretchy smart fabrics and self-powered sensing, *Sci. Adv*., **7**, 22 (2021)

[S4] Liu, S., Shah, D.S. & Kramer-Bottiglio, R. Highly stretchable multilayer electronic circuits using biphasic gallium-indium. *Nat. Mater.* **20**, 851–858 (2021)

[S5] Ma, Z., Huang, Q., Xu, Q. *et al.* Permeable superelastic liquid-metal fibre mat enables biocompatible and monolithic stretchable electronics. *Nat. Mater.* **20**, 859–868 (2021)

[S6] Li, Y., Fan, T., Zhang, J., Kong, D., Ultrasensitive and ultrastretchable electrically self-healing conductors, *Proceedings of the National Academy of Sciences,* **120**, 23 (2023)

[S7] Lopes, P.A., Santos, B.C., de Almeida, A.T. *et al.* Reversible polymer-gel transition for ultra-stretchable chip-integrated circuits through self-soldering and self-coating and self-healing. *Nat Commun* **12**, 4666 (2021)

[S8] Zheng, S., Wang, X., Li, W. *et al.* Pressure-stamped stretchable electronics using a nanofibre membrane containing semi-embedded liquid metal particles. *Nat Electron* **7**, 576–585 (2024)

[S9] Xu, Y. et al., Porous liquid metal–elastomer composites with high leakage resistance and antimicrobial property for skin-interfaced bioelectronics, *Sci. Adv.,* **9**, 1 (2023)

[S10] Zhu, H., Wang, S., Zhang, M. *et al.* Fully solution processed liquid metal features as highly conductive and ultrastretchable conductors. *npj Flex Electron* **5**, 25 (2021)

[S11] Parida, K., Thangavel, G., Cai, G. *et al.* Extremely stretchable and self-healing conductor based on thermoplastic elastomer for all-three-dimensional printed triboelectric nanogenerator. *Nat Commun* **10**, 2158 (2019). https://doi.org/10.1038/s41467-019-10061-y

[S12] H.-W. Lin, C.-T. Wu, C.-W. Chang, T.-Y. Lo, H.-R. Chen, and J.-T. Chen, "Enhancing Conductivity and Self-Healing in PEDOT:PSS/Poly(ionic liquid) Elastomers for Sensor Applications," ACS Appl Polym Mater, vol. 6, no. 23, pp. 14740–14748, Dec. 2024, doi: 10.1021/acsapm.4c02949.

[S13] Z. Ma, Z. Liu, J. Zou, H.-Y. Mi, Y. Liu, and X. Jing, "Self-healable and Robust Silicone Elastomer for Ultrasensitive Flexible Sensors," ACS Sustain Chem Eng, vol. 11, no. 28, pp. 10496–10508, Jul. 2023, doi: 10.1021/acssuschemeng.3c02168.

[S14] X. Wang, L. Weng, X. Zhang, Z. Wu, L. Guan, and X. Li, "A Self‐Healing Conductive Elastomer Based on a Polymerizable Deep Eutectic Solvent," Small, vol. 20, no. 11, Mar. 2024, doi: 10.1002/smll.202304828.

[S15] C. Luo et al., "A Fully Self‐Healing and Highly Stretchable Liquid‐Free Ionic Conductive Elastomer for Soft Ionotronics," Adv Funct Mater, vol. 33, no. 49, Dec. 2023, doi: 10.1002/adfm.202304486.

[S16] Z. Wang et al., "Tough, Self-Healing, and Conductive Elastomer—Ionic PEGgel," ACS Appl Mater Interfaces, vol. 14, no. 44, pp. 50152–50162, Nov. 2022, doi: 10.1021/acsami.2c14394.

[S17] D. Du, J. Zhou, D. Shi, W. Dong, and M. Chen, "Cross-Linked, Transient Ionic Conductive Elastomer with Extreme Stretchability, Healability, and Degradability for Detecting Human Motions," ACS Appl Polym Mater, vol. 4, no. 7, pp. 4972–4979, Jul. 2022, doi: 10.1021/acsapm.2c00527.

[S18] G. Su et al., "Balancing the mechanical, electronic, and self-healing properties in conductive self-healing hydrogel for wearable sensor applications," Mater Horiz, vol. 8, no. 6, pp. 1795–1804, 2021, doi: 10.1039/D1MH00085C.

[S19] X. Xu, Z. Li, Y. Huang, Z. Zhang, and S. Xiong, "Tailoring high mechanical performances of self‐healable poly(vinyl alcohol)‐based elastomers via judicious grafting of side chains," Polym Int, vol. 72, no. 7, pp. 648–654, Jul. 2023, doi: 10.1002/pi.6515.

[S20] J. Fan, J. Huang, Z. Gong, L. Cao, and Y. Chen, "Toward Robust, Tough, Self-Healable Supramolecular Elastomers for Potential Application in Flexible Substrates," ACS Appl Mater Interfaces, vol. 13, no. 1, pp. 1135–1144, Jan. 2021, doi: 10.1021/acsami.0c15552.

[S21] W. Liu et al., "Healable, Recyclable, and High-Stretchable Polydimethylsiloxane Elastomer Based on Synergistic Effects of Multiple Supramolecular Interactions," Macromol Mater Eng, vol. 307, no. 10, Oct. 2022, doi: 10.1002/mame.202200310.

[S22] Y. Lang, W. Zheng, W. Wang, and Z. Li, "A super-stretched self-healing silicone elastomer based on high molecular weight polydimethylsiloxanes through intermolecular quadruple hydrogen bonding," Polym Adv Technol, vol. 34, no. 9, pp. 2823–2830, Sep. 2023, doi: 10.1002/pat.6107.

[S23] J. Fan, W. Wu, X. Zeng, J. Zhang, H. Zhang, and H. He, "Dual Reversible Network Nanoarchitectonics for Ultrafast Light-Controlled Healable and Tough Polydimethylsiloxane-Based Composite Elastomers," ACS Appl Mater Interfaces, vol. 15, no. 32, pp. 38996–39007, Aug. 2023, doi: 10.1021/acsami.3c08041.

[S24] F. Wang et al., "A hybrid reversible crosslinked polysiloxane elastomer with high toughness and recyclability," Journal of Polymer Science, vol. 62, no. 10, pp. 2147–2156, May 2024, doi: 10.1002/pol.20240019.

[S25] B. Zhang et al., “A Transparent, Highly Stretchable, Autonomous Self-Healing Poly(dimethyl siloxane) Elastomer,” Macromol Rapid Commun, vol. 38, no. 15, Aug. 2017, doi: 10.1002/marc.201700110.

[S26] X. Wu, J. Wang, J. Huang, and S. Yang, “Robust, Stretchable, and Self-Healable Supramolecular Elastomers Synergistically Cross-Linked by Hydrogen Bonds and Coordination Bonds,” ACS Appl Mater Interfaces, vol. 11, no. 7, pp. 7387–7396, Feb. 2019, doi: 10.1021/acsami.8b20303.

[S27] K. Song et al., “Synergy between dynamic covalent boronic ester and boron–nitrogen coordination: strategy for self-healing polyurethane elastomers at room temperature with unprecedented mechanical properties,” Mater Horiz, vol. 8, no. 1, pp. 216–223, 2021, doi: 10.1039/D0MH01142H.

[S28] Y. Wang, X. Huang, and X. Zhang, “Ultrarobust, tough and highly stretchable self-healing materials based on cartilage-inspired noncovalent assembly nanostructure,” Nat Commun, vol. 12, no. 1, p. 1291, Feb. 2021, doi: 10.1038/s41467-021-21577-7.

[S29] W. Zhang, M. Ren, M. Chen, and L. Wu, “Tough and self-healing waterborne polyurethane elastomers via hydrogen bonds and oxime–carbamate design for wearable flexible strain sensors,” RSC Adv, vol. 15, no. 8, pp. 6231–6240, 2025, doi: 10.1039/D4RA09084E.

[S30] L. Xia et al., “A room-temperature self-healing elastomer with ultra-high strength and toughness fabricated via optimized hierarchical hydrogen-bonding interactions,” J Mater Chem A Mater, vol. 10, no. 8, pp. 4344–4354, 2022, doi: 10.1039/D1TA08748G.

[S31] Y. Li et al., “A self-reinforcing and self-healing elastomer with high strength, unprecedented toughness and room-temperature reparability,” Mater Horiz, vol. 8, no. 1, pp. 267–275, 2021, doi: 10.1039/D0MH01447H.

[S32] X. Wang et al., “Self-Healing Polyurethane Elastomers with Superior Tensile Strength and Elastic Recovery Based on Dynamic Oxime-Carbamate and Hydrogen Bond Interactions,” Macromol Rapid Commun, vol. 45, no. 13, Jul. 2024, doi: 10.1002/marc.202400022.

[S33] X. Wu, M. Li, H. Li, H. Gao, Z. Wang, and Z. Wang, “Autonomous Underwater Self-Healable Adhesive Elastomers Enabled by Dynamical Hydrophobic Phase-Separated Microdomains,” Small, vol. 20, no. 35, Aug. 2024, doi: 10.1002/smll.202311131.

[S34] Lee, Yongjun, Beom Jin Kim, Luhing Hu, Juyeong Hong, and Jong-Hyun Ahn. Morphable 3D structure for stretchable display. Materials Today 53 (2022): 51-57. https://doi.org/10.1016/j.mattod.2022.01.017

[S35] Jung, D., Ju, H., Cho, S. et al. Multilayer stretchable electronics with designs enabling a compact lateral form. npj Flex Electron 8, 13 (2024). https://doi.org/10.1038/s41528-024-00299-y

[S36] Zhu, H., Wang, S., Zhang, M. et al. Fully solution processed liquid metal features as highly conductive and ultrastretchable conductors. npj Flex Electron 5, 25 (2021). https://doi.org/10.1038/s41528-021-00123-x

[S37] Nam, M., Chang, J., Kim, H. et al. Highly reliable and stretchable OLEDs based on facile patterning method: toward stretchable organic optoelectronic devices. npj Flex Electron 8, 17 (2024). https://doi.org/10.1038/s41528-024-00303-5

[S38] Ang, L., Wang, H., Ren, J. et al. Highly robust soft-rigid connections via mechanical interlocking for assembling ultra-stretchable displays. npj Flex Electron 8, 50 (2024). https://doi.org/10.1038/s41528-024-00337-9

[S39] Liu, S., Shah, D.S. & Kramer-Bottiglio, R. Highly stretchable multilayer electronic circuits using biphasic gallium-indium. Nat. Mater. 20, 851–858 (2021). https://doi.org/10.1038/s41563-021-00921-8

[S40] M. Miyakawa, H. Tsuji, T. Takei, T. Yamamoto, Y. Fujisaki, M. Nakata, Highly Stretchable LED Display Using Liquid Metal and Molybdenum-Barriered Multilayer Electrodes with Long-Term Reliability. Adv. Electron. Mater. 2025, 11, 2400676. https://doi.org/10.1002/aelm.202400676

[S41] S. Chen, S. Fan, J. Qi, Z. Xiong, Z. Qiao, Z. Wu, J. C. Yeo, C. T. Lim, Ultrahigh Strain-Insensitive Integrated Hybrid Electronics Using Highly Stretchable Bilayer Liquid Metal Based Conductor. Adv. Mater. 2023, 35, 2208569. https://doi.org/10.1002/adma.202208569

[S42] H. Zhan, B. Wen, B. Tian, K. Zheng, Q. Li, W. Wu, Printed Self-Healing Stretchable Electronics for Bio-signal Monitoring and Intelligent Packaging. Small 2024, 20, 2400740. https://doi.org/10.1002/smll.202400740

[S43] C. Park, J. Kim, H. Lee, J. Lee, M. M. Islam, H. Jeong, J. Jang, Special Section: Liquid Metals for Functional Materials. Adv. Mater. Technol. 2024, 9, 2301413. https://doi.org/10.1002/admt.202301413
